# Mutant Muscle LIM Protein C58G causes cardiomyopathy through protein depletion

**DOI:** 10.1016/j.yjmcc.2018.07.248

**Published:** 2018-08

**Authors:** Mehroz Ehsan, Matthew Kelly, Charlotte Hooper, Arash Yavari, Julia Beglov, Mohamed Bellahcene, Kirandeep Ghataorhe, Giulia Poloni, Anuj Goel, Theodosios Kyriakou, Karin Fleischanderl, Elisabeth Ehler, Eugene Makeyev, Stephan Lange, Houman Ashrafian, Charles Redwood, Benjamin Davies, Hugh Watkins, Katja Gehmlich

**Affiliations:** a Division of Cardiovascular Medicine, Radcliffe Department of Medicine, British Heart Foundation Centre of Research Excellence, University of Oxford, Oxford, UK; b Wellcome Centre for Human Genetics, University of Oxford, Oxford, UK; c Experimental Therapeutics, Radcliffe Department of Medicine, University of Oxford, UK; d Randall Centre for Cell and Molecular Biophysics, School of Cardiovascular Medicine and Sciences, King's College London BHF Centre of Research Excellence, London, UK; e Centre for Developmental Neurobiology, King's College London, London, UK; f School of Medicine, University of California, San Diego, La Jolla, CA 92093, USA; g Transgenic Core, Wellcome Centre for Human Genetics, University of Oxford, Oxford, UK

**Keywords:** Hypertrophic cardiomyopathy, MLP C58G mutation, Mouse knock-in model, *In vivo* phenotyping, RNAseq transcriptome analysis, Protein depletion, Proteasome, Sarcomere

## Abstract

*Cysteine and glycine rich protein 3 (CSRP3)* encodes Muscle LIM Protein (MLP), a well-established disease gene for Hypertrophic Cardiomyopathy (HCM). MLP, in contrast to the proteins encoded by the other recognised HCM disease genes, is non-sarcomeric, and has important signalling functions in cardiomyocytes. To gain insight into the disease mechanisms involved, we generated a knock-in mouse (KI) model, carrying the well documented HCM-causing *CSRP3* mutation C58G.

*In vivo* phenotyping of homozygous KI/KI mice revealed a robust cardiomyopathy phenotype with diastolic and systolic left ventricular dysfunction, which was supported by increased heart weight measurements. Transcriptome analysis by RNA-seq identified activation of pro-fibrotic signalling, induction of the fetal gene programme and activation of markers of hypertrophic signalling in these hearts. Further *ex vivo* analyses validated the activation of these pathways at transcript and protein level. Intriguingly, the abundance of MLP decreased in KI/KI mice by 80% and in KI/+ mice by 50%. Protein depletion was also observed in cellular studies for two further HCM-causing *CSRP3* mutations (L44P and S54R/E55G). We show that MLP depletion is caused by proteasome action. Moreover, MLP C58G interacts with Bag3 and results in a proteotoxic response in the homozygous knock-in mice, as shown by induction of Bag3 and associated heat shock proteins.

In conclusion, the newly generated mouse model provides insights into the underlying disease mechanisms of cardiomyopathy caused by mutations in the non-sarcomeric protein MLP. Furthermore, our cellular experiments suggest that protein depletion and proteasomal overload also play a role in other HCM-causing *CSPR3* mutations that we investigated, indicating that reduced levels of functional MLP may be a common mechanism for HCM-causing *CSPR3* mutations.

## Highlights

• We present a mouse model for non-sarcomeric hypertrophic cardiomyopathy (HCM).
• Homozygous Muscle LIM Protein (MLP) C58G mice have systolic and diastolic dysfunction.
• MLP C58G is depleted *via* proteasomal pathways.
• Protein depletion is also a hallmark of further HCM causing MLP mutations.
• MLP C58G interacts with Bag3 and causes a proteotoxic response.

## 1 Introduction

Muscle LIM protein (MLP) is predominantly expressed in striated muscle tissues [1]. It had originally been identified as a Z-disc protein [2], but was also subsequently detected in the cytoplasm and the nucleus [3, 4]. Importantly, we have shown that it is not an integral part of the cardiac sarcomere: MLP co-purifies with cytosolic, but not with sarcomeric proteins and can be extracted under mild conditions where sarcomeric structures are preserved [4]. In agreement with its multiple sub-cellular localisations, various binding partners have been identified in the Z-disc, at the membranes and in the nucleus (reviewed in [5]). MLP's function in the heart was initially suggested to be part of a Z-disc based stretch sensor [2], but more recent work has reclassified MLP's functions away from stretch sensing [4], to an alternative role of modulating protein kinase C alpha (PKCα) activity in the myocardium [6].

The crucial physiological role of MLP in cardiac integrity is highlighted by the fact that deletion of its encoding gene (cysteine and glycine rich protein 3, *Csrp3*) causes dilated cardiomyopathy (DCM) in mice [7]. Mutations in the human *CSRP3* gene have subsequently been associated with autosomal dominant DCM and hypertrophic cardiomyopathy (HCM) [[8], [9], [10], [11]]. A recent study has demonstrated an excess of *CSRP3* variants in HCM cases over control populations [12], providing confidence that *CSRP3* mutations do indeed contribute to HCM. For one of these mutations, *CSRP3* p.C58G, there is strikingly clear genetic evidence of a causative role, supported by co-segregation with a HCM phenotype in a large four-generation family and genome-wide linkage analysis [4]. At the molecular level, the mutation is thought to abolish the coordination of a zinc ion in the first LIM domain, known to be crucial for the stability of the domain. Hence, *CSRP3* is perhaps the best-documented example of a gene encoding a non-sarcomeric protein that is mutated in hereditary non-syndromic HCM.

Notably, the underlying pathogenic mechanisms of *CSRP3* mutations remain unknown. Analysis of myocardium from a patient harbouring the *CSRP3* p.C58G mutation showed a reduction in MLP level [4], but the interpretation of this finding from a single sample of a patient with advanced disease is potentially confounded by the fact that MLP expression is highly variable in the myocardium of non-failing controls and diseased tissue [6, 13, 14].

To address this challenge, we have generated a mouse model carrying the C58G mutation (*Csrp3* C58G knock-in, KI). This is the first reported knock-in mouse model mirroring the human genetic situation of a clearly pathogenic (non-syndromic) HCM variant in a non-sarcomeric protein and, hence, represents a valuable tool in addressing the disease mechanisms of non-sarcomeric HCM. Comprehensive phenotyping of these mice identified a robust cardiomyopathy phenotype in the homozygous setting. At the molecular level, unbiased transcriptome profiling combined with analyses of specific gene and protein candidates provided clear evidence of cardiac remodelling and activation of hypertrophic signalling pathways, including calcineurin signalling, in KI homozygotes.

Importantly, we detected a marked reduction in MLP levels. Using a combination of *in vitro* and *in vivo* approaches, we have linked this protein depletion with increased activity of the ubiquitin-proteasomal system (UPS). Moreover, our cellular experiments demonstrated that this protein depletion is a hallmark of multiple HCM-causing MLP mutations investigated, suggesting that UPS-mediated depletion of MLP is the unifying underlying driver for the development of non-sarcomeric HCM caused by mutations in *CSRP3*.

## 2 Results

### 2.1 Generation of *Csrp3* C58G knock-in mice

To generate a model system for human HCM caused by a mutation in *CSRP3*, the C58G mutation was introduced into exon 3 of the murine *Csrp3* gene by homologous recombination and subsequent removal of selection markers using FLPe/FRT recombination (Fig. S1A). Mice carrying the mutation (either heterozygous or homozygous) were viable, fertile and had a normal life span up to the observed 18 months of age. The mutation was detectable at both mRNA (Fig. S2A) and protein level (Fig. S2B, C). Unless stated otherwise, phenotyping was performed in young adult male mice (2–3 month of age).

### 2.2 Cardiac phenotype of heterozygous and homozygous *Csrp3* C58G KI mice

**Fig. 1:**
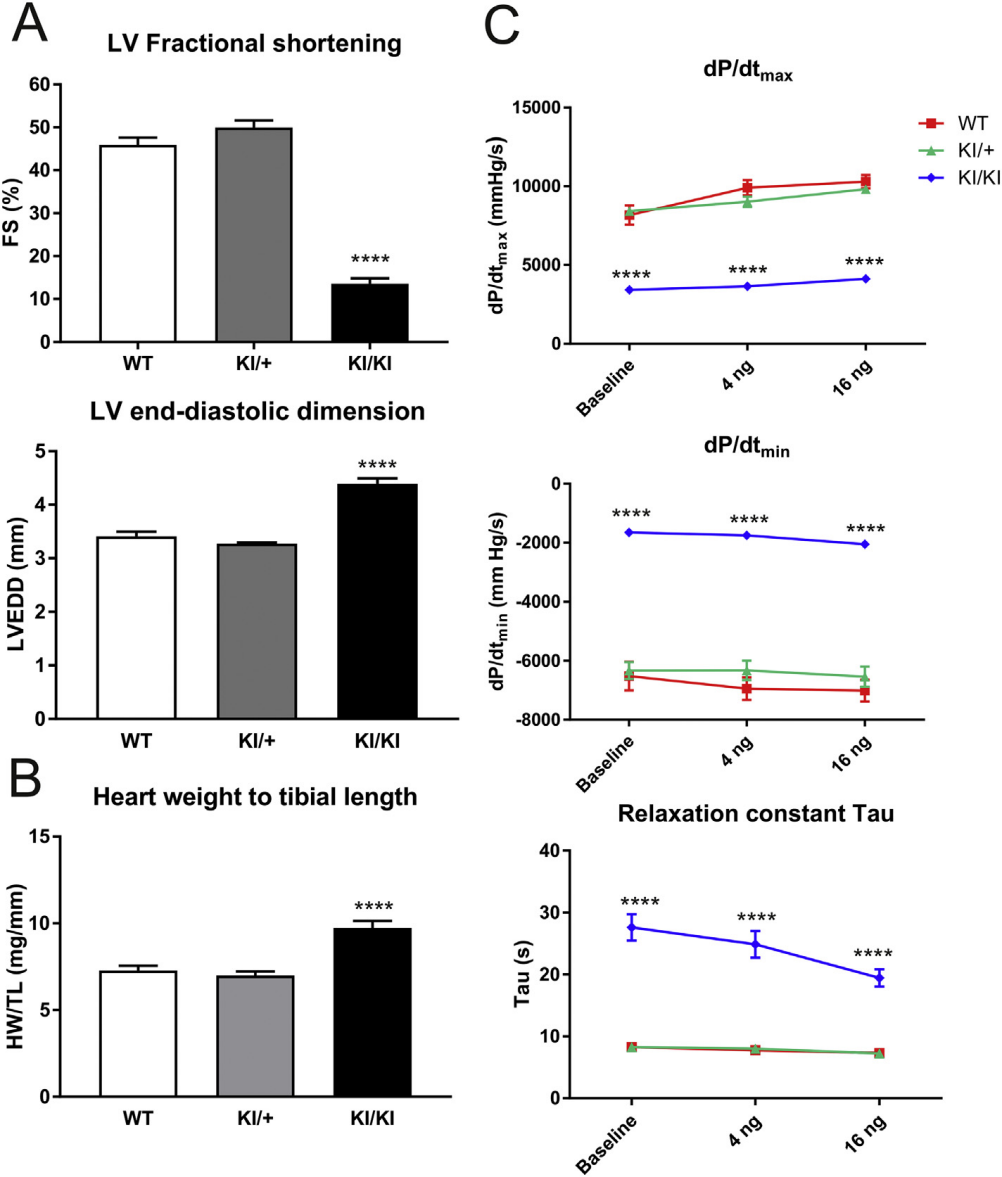
Cardiac phenotype of KI/+ and KI/KI mice in comparison to WT mice. A – Characterisation of cardiac dimensions and function by echocardiography: Fractional shortening and end-diastolic dimensions are shown. For cohort characteristics and a wider set of echocardiographic parameters please refer to Table S1. B – Heart weight is increased in KI/KI mice, values are normalised to tibial length. ****p < 0.0001 *versus* WT. C – Invasive haemodynamic assessment of LV performance of the three genotypes: dP/dt_max_, dP/dt_min_ and relaxation constant Tau are shown at baseline conditions, and under adrenergic stress (dobutamine infusion at 4 ng g^−1^ BW min^−1^ and 16 ng g^−1^ BW min^−1^). Where error bars are missing, they are smaller than the symbols. For cohort characteristics and a wider set of parameters refer to Table S2.

Male mice heterozygous or homozygous for the *Csrp3* C58G mutation (hereafter referred to as KI/+ and KI/KI, respectively) were compared to wildtype (WT) controls by transthoracic echocardiography (Fig. 1A). KI/+ mice had normal cardiac dimensions and function for all parameters investigated, except for an increase of anterior wall thickness at the 6 month timepoint (Table S1). In contrast, KI/KI mice had reduced systolic function, enlarged left ventricular (LV) dimensions and elevated calculated LV mass. In agreement with these *in vivo* measurements, heart weight (normalised to tibial length) was increased for KI/KI mice, but not for KI/+ mice (Fig. 1B).

Invasive LV haemodynamic measurements were performed in all three genotypes, both at baseline and upon adrenergic stimulation (dobutamine infusion at two different concentrations; Fig. 1C; Table S2). Once again, KI/+ showed no significant differences to WT mice under all conditions examined, whereas KI/KI mice had marked diastolic and systolic impairment, as evidenced by increased relaxation constant Tau and blunted dP/dt_max_ and dP/dt_min_, as well as markedly reduced contractile reserve in response to beta-adrenergic stimulation (Fig. 1C, Table S2).

As KI/+ mice did not display an overt phenotype at baseline, we examined the response of KI/+ mice to mechanical stress induced by increased LV afterload using transverse aortic constriction (TAC) surgery [15] in an attempt to unmask a subtle phenotype arising due to the C58G mutation. We observed robust LV hypertrophy at 2 weeks post-TAC in all banded animals (Fig. S3A). However, the macroscopic and molecular hypertrophic response to chronic pressure overload did not differ for KI/+ mice when compared to their WT littermates (Fig. S3, Table S3).

In summary, KI/+ mice did not display a cardiac phenotype at baseline or upon challenge by LV pressure overload. In contrast, KI/KI mice displayed a clear cardiomyopathy phenotype, with impaired systolic and diastolic function, increased cardiac mass and reduced contractile reserve.

### 2.3 Transcriptomic profiling of *Csrp3* C58G KI mice

To gain insights into the molecular changes underlying the cardiac phenotype observed in the presence of the MLP C58G mutation, we performed transcriptome profiling by RNA-seq on WT and KI/KI mouse hearts. 622 genes were found to be differentially expressed (Table S4).

**Fig. 2:**
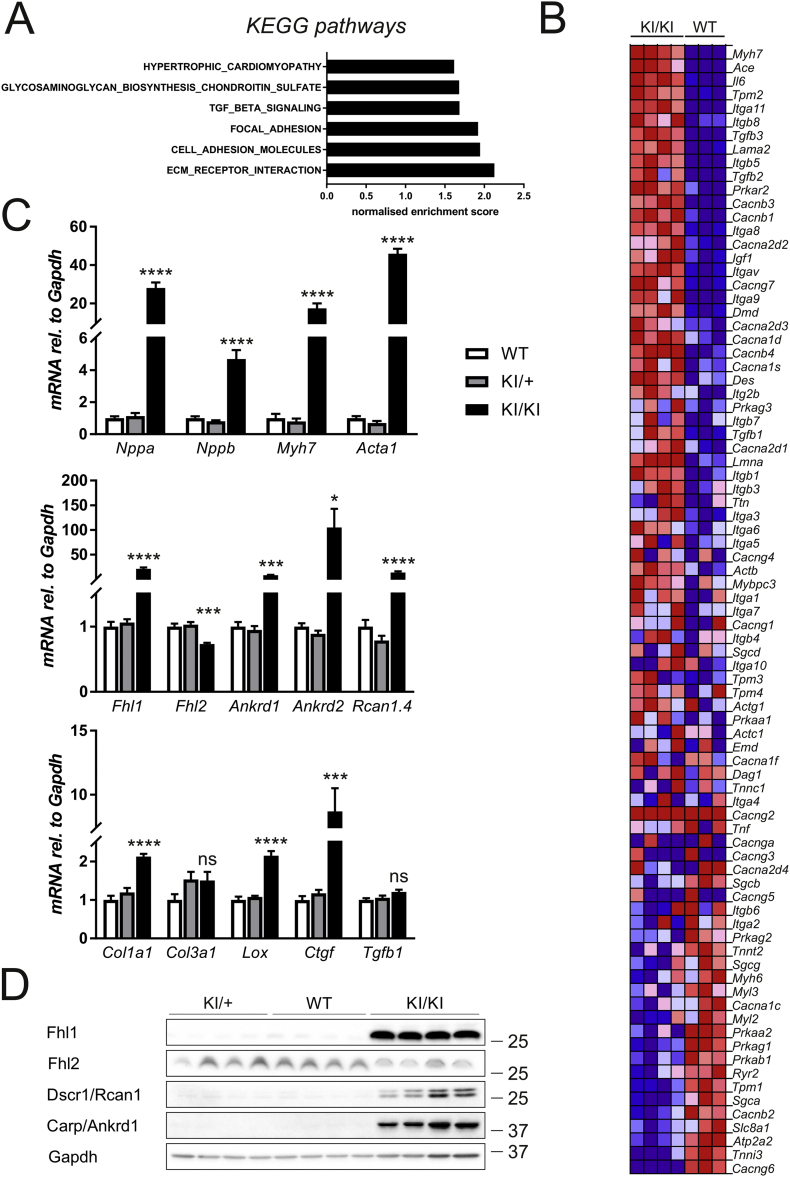
Molecular phenotype of KI/+ and KI/KI hearts. A, B – Transcriptome profiling by RNAseq: A – KEGG Pathway analysis: The top five statistically (p < 0.001) enriched GO terms for KI/KI hearts when compared to WT hearts are shown. B – Heat map shows genes in the KEGG HCM gene set with the strongest up-regulation in the KI/KI hearts at the top and the genes showing the strongest down-regulation at the bottom. Colours range from dark red to dark blue representing respectively the highest and lowest expression of a gene. C – Assessment of transcriptional changes by qPCR for genes related to the fetal gene programme (top), hypertrophic signalling (middle) and fibrosis (bottom). All measurements are normalised to *Gapdh*; significant changes are observed in the hearts of KI/KI mice; *p < 0.05, ***p < 0.001, ****p < 0.0001 *versus* WT, n = 6 per group. D – Changes in hypertrophic signalling proteins assessed by Western blotting: Fhl1, Carp (Ankrd1) and Dscr1 (Rcan1) are induced in KI/KI hearts, in line with the activation of hypertrophic signalling pathways. Anti-hypertrophic Fhl2 is down-regulated in these hearts (for quantification see Fig. S5B). Gapdh serves as loading control. The position of marker proteins is indicated (molecular weight in kDa). (For interpretation of the references to colour in this figure legend, the reader is referred to the web version of this article.)

To aid functional interpretation of the differential transcriptome, we analysed enriched gene ontology (GO) terms and Kyoto Encyclopedia of Genes and Genomics (KEGG) pathways. The most enriched KEGG pathways included extracellular matrix receptor interaction, cell and focal adhesion, transforming growth factor beta signalling and glycosaminoglycan biosynthesis, indicating enhanced fibrotic extracellular matrix remodelling in the KI/KI hearts (Fig. 2A). Gene Set Enrichment Analysis also identified the HCM-related KEGG pathway as one of the significantly enriched pathways (Fig. 2A): the heat map (Fig. 2B) highlights the genes within this particular KEGG pathway, showing the activation of hypertrophic signalling within the differentially expressed gene set. GOrilla analysis was performed using differentially expressed genes and identified several enriched GO terms for these genes. In agreement with alterations in the transcriptome, the most enriched GO cellular components were “sarcomere” and “extracellular matrix”, while for GO processes “heart rate” and “cellular proliferation” were found to be most enriched (Fig. S4).

### 2.4 Molecular characteristics of heterozygous and homozygous *Csrp3* C58G KI mice

Based on these results, hearts of mice from all three genotypes were investigated using more targeted molecular and histological analyses. In agreement with a macroscopic cardiomyopathy phenotype and the altered transcriptome profile, qPCR showed striking induction of transcripts related to heart failure and hypertrophic signalling in KI/KI mice (Fig. 2C), including induction of the fetal gene programme [16]. Moreover, transcripts known to be linked to extracellular matrix remodelling and fibrosis were upregulated in these mice (Fig. 2C), while only one transcript related to apoptosis (*Bcl2*) was found to be upregulated (Fig. S5A).

Induction of proteins implicated in hypertrophic signalling, such as Fhl1, Ankrd1/cardiac ankyrin repeat protein (Carp) and calcineurin responsive Rcan1.4 was confirmed by Western blotting in KI/KI mice (Fig. 2D), together with a down-regulation of anti-hypertrophic Fhl2 (Fig. 2D, Fig. S5B). In addition, upregulation of β-myosin heavy chain was confirmed at protein level (Fig. S5C). Histological analysis showed no differences between WT and KI/+ mice, with no evidence of fibrosis (Fig. S6). In contrast, KI/KI mice showed signs of fibrosis, albeit mild, on Sirius Red staining (Fig. S6), confirming the induction of pro-fibrotic signalling at transcript level. Taken together, at the molecular level the hearts of KI/KI mice are characterised by induction of the fetal gene programme and upregulation of markers of hypertrophy and heart failure.

### 2.5 Analysis of MLP expression in *Csrp3* C58G KI mice

**Fig. 3:**
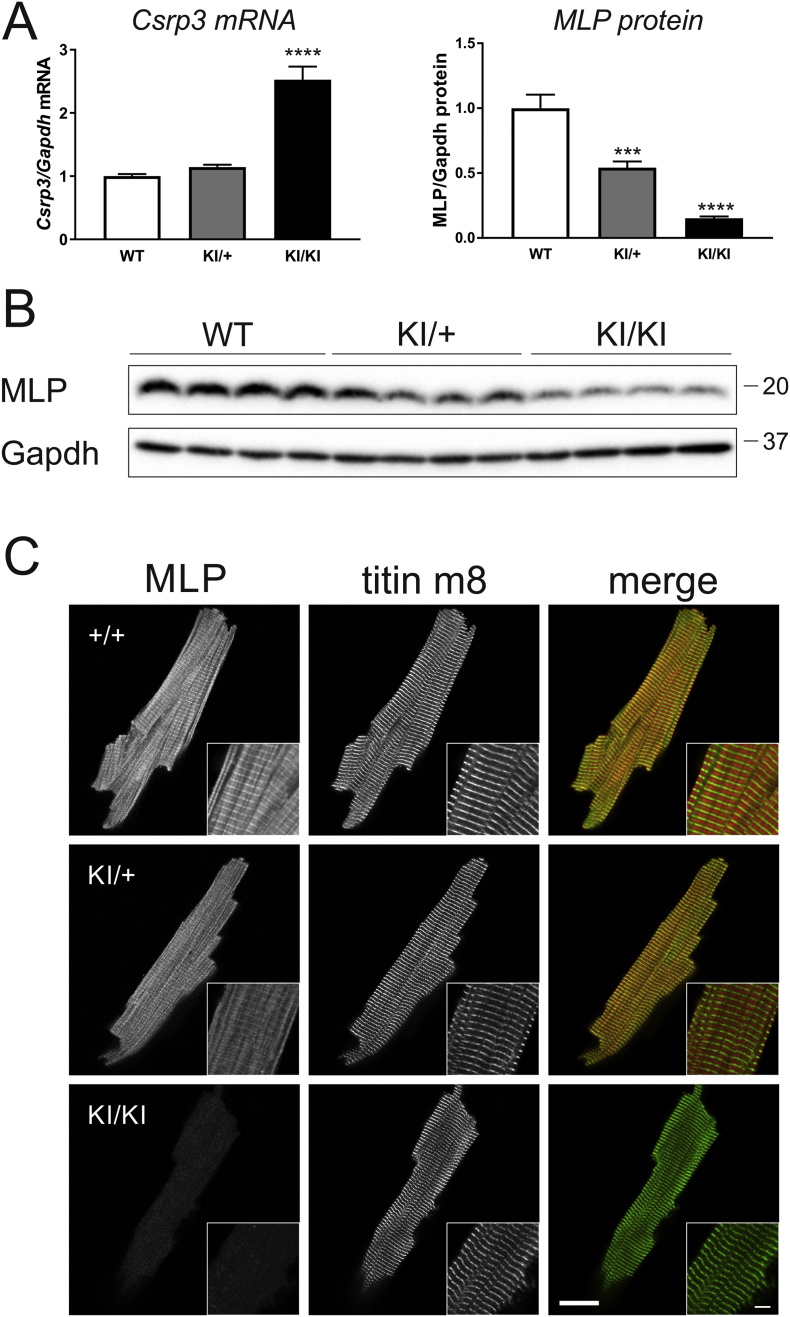
MLP depletion in KI/KI hearts. A – Measurement of *Csrp3* transcript by qPCR (left) and MLP protein (right) in the three groups, normalised to Gapdh transcript and protein, respectively. In the KI/KI mice, *Csrp3* transcript is 2.5-fold up-regulated. The abundance of MLP decreased in KI/KI mice by 80% and in KI/+ mice by 50%. MLP level is down-regulated to 50% in KI/+ mice and to 20% in KI/KI mice. ***p < 0.001, ****p < 0.0001 *versus* WT, n = 6 per group. B – Representative Western blot demonstrating reduced MLP levels in KI/+ and KI/KI mice. Gapdh serves as loading control. The position of marker proteins is indicated (molecular weight in kDa). N.B., both TaqMan probe target site and epitope of the anti-MLP antibody 79D2 are not overlapping with the site of the C58G mutation. C – Immuno-fluorescence on isolated adult cardiomyocytes from mice of the three genotype groups. Cells are stained for MLP (left), titin M-band epitope m8 (middle) and merged images are shown on the right (MLP red, titin m8 green). Scale bar represents 20 μm. Insert: Magnified area, scale bar represents 5 μm.

To assess whether the mutation resulted in any changes to mRNA and/or protein expression, *Csrp3* transcript expression and MLP levels were measured. *Csrp3* transcript was upregulated in KI/KI, but not in KI/+ mice (Fig. 3A). Surprisingly, however, the abundance of the MLP decreased in KI/KI mice by 80% and in KI/+ mice by 50% of the WT level (Fig. 3A, B). This finding was confirmed by immuno-fluorescence staining of isolated adult cardiomyocytes. Indeed, the MLP specific immunoreactive signal was lower in KI/+ cells than in WT cells, and this effect was even more pronounced in KI/KI cells (Fig. 3C). Of note, the localisation of the remaining MLP was diffuse with some Z-disc staining in KI/+ cells, and diffuse in KI/KI cells. No nuclear accumulation or aggregation of MLP was observed.

### 2.6 The *Csrp3* C58G KI allele cannot rescue the phenotype in *Csrp3*/MLP knock out mice

**Fig. 4:**
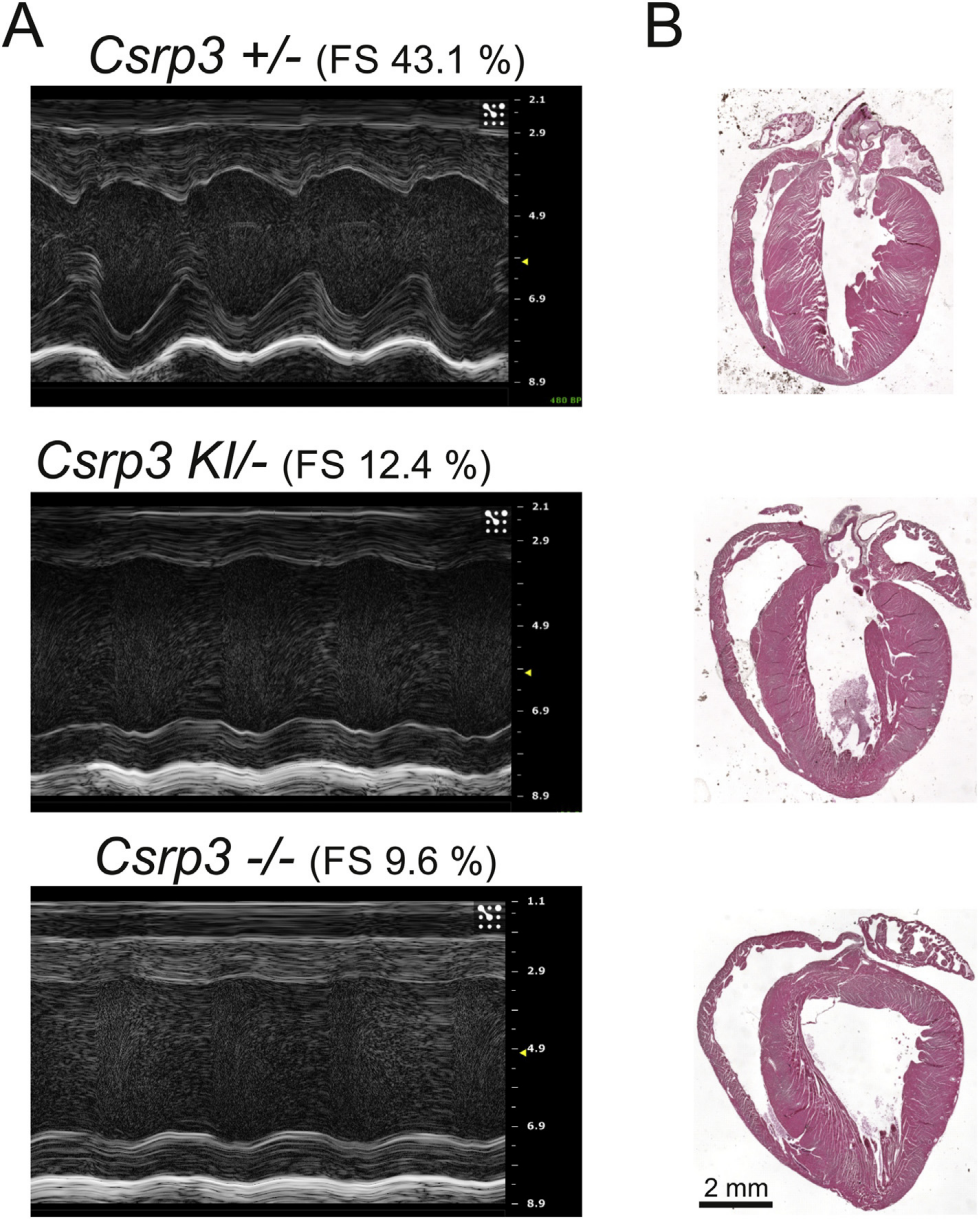
The *Csrp3* C58G allele cannot rescue the phenotype of *Csrp3*-/- knock out mice. A – Representative examples of M-mode echocardiography of *Csrp3*+/- mice with normal systolic function (top), *Csrp3* KI/- mice (middle) and *Csrp3*-/- mice (bottom), the latter both with impaired systolic function. The calculated value of LV fractional shortening (FS) is given for each mouse. B – Cardiac morphology of the genotypes. Haematoxylin-eosin stained paraffin heart sections are shown.

Inactivation of both alleles of the *Csrp3* gene (*Csrp3*-/-; also known in the literature as MLP knock out, KO, mice) leads to a striking cardiac phenotype resembling DCM [7], while hemizygous *Csrp3*+/- have normal cardiac dimensions and function at baseline ([17] and Fig. 4 and Fig. S7B, Table S5). To further address the genetic consequences of the C58G mutation, we generated mice with one *Csrp3* null allele and a *Csrp3* C58G allele (designated *Csrp3* KI/-). These mice were found to have a similar level of LV dilatation and reduced fractional shortening to *Csrp3*-/- mice at 2 months of age (Fig. 4, Table S6), indicating that the *Csrp3* C58G allele cannot functionally compensate for the loss of the *Csrp3* WT allele on the *Csrp3* null background.

### 2.7 Protein depletion is a common feature of HCM-associated *Csrp3* mutations

**Fig. 5:**
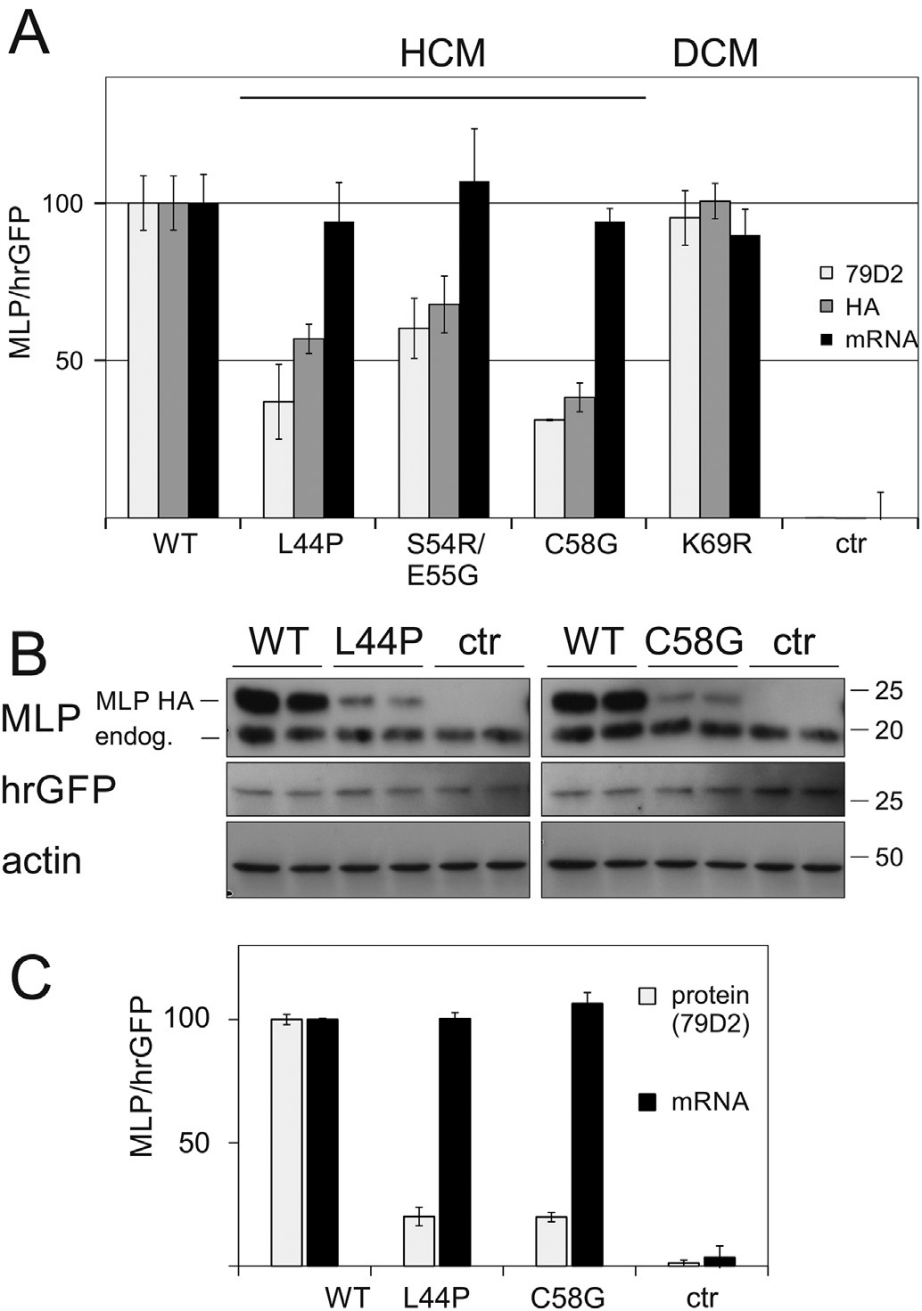
Protein depletion is a hallmark of HCM-associated MLP mutations. A – Recombinant HA-tagged MLP WT, HCM-associated MLP mutants (L44P, S44R/E54G, C58G), a DCM-associated MLP mutant (K69R) and empty parental vector were expressed in HEK cells, by transfection with a bicistronic vector system (see Section 1.6 of Material and Methods in Appendix A Supplementary data). Cell lysates were blotted for MLP (using either anti-HA antibody against the N-terminal tag, light grey, or anti-MLP antibody 79D2 against the C-terminus of the protein, dark grey) and hrGFP. In addition, *CSRP3* and hrGFP transcripts were measured by qPCR in parallel transfected cells (black bars). The ratios of *CSRP3* transcript or MLP protein to hrGFP were set to 100% for WT transfections and all other samples expressed relative to WT, n = 3 per group. Note that all HCM-associated mutants are affected by protein depletion, while the DCM-associated mutation is not affected. B – NRC were transduced with adenoviral particles coding for MLP WT, L44P, C58G and empty vector (see A) and cell lysates were blotted for MLP (using antibody 79D2) and hrGFP. The HA-tag on the recombinant MLP allows separation of this protein from endogenous rat MLP by size. Pan-actin serves as a loading control. The position of marker proteins is indicated (molecular weight in kDa). C – Quantification of Western blots from panel B (grey bars) and measurement of *CSRP3* and hrGFP transcripts by qPCR to confirm equal transduction efficiency (back bars). Data are normalised as in panel A. The TaqMan probe is specific for the recombinant (human) MLP constructs and does not recognise the endogenous rat *Csrp3* transcript.

The observed dramatic reduction of MLP levels in *Csrp3* C58G KI mice suggested that protein depletion could be a common feature of HCM-associated *Csrp3* mutations. To investigate this, C58G and two additional HCM-causing MLP mutations (L44P and S54R/E55G, [9]) were expressed in mammalian cells in a bicistronic system with a hrGFP reporter protein. Upon normalisation to hrGFP, all three HCM-associated mutations showed reduced protein levels, while transcript levels were normal, indicating comparable transfection efficiency (Fig. 5A, Fig. S8A). In contrast, a DCM mutation (K69R, [11]), showed similar protein levels to MLP WT control.

For a more detailed assessment, two HCM mutations (MLP L44P and C58G) were expressed in primary neonatal rat cardiomyocytes (NRC) using the bicistronic reporter system and adenoviral gene delivery. Both mutant proteins were expressed at approximately 20% of the level of WT recombinant protein, with no dominant negative effect on endogenous MLP expression observed (Fig. 5B, C). The protein depletion was further confirmed using adult Guinea pig cardiomyocytes (Fig. S8B); WT recombinant protein was detectable as diffuse cytoplasmic protein 48 h post-infection. In contrast, only traces of MLP L44P and MLP C58G were observed in cells, despite the reporter protein hrGFP being clearly present in infected cells.

### 2.8 The UPS is responsible for the protein depletion of HCM-associated MLP mutants

**Fig. 6:**
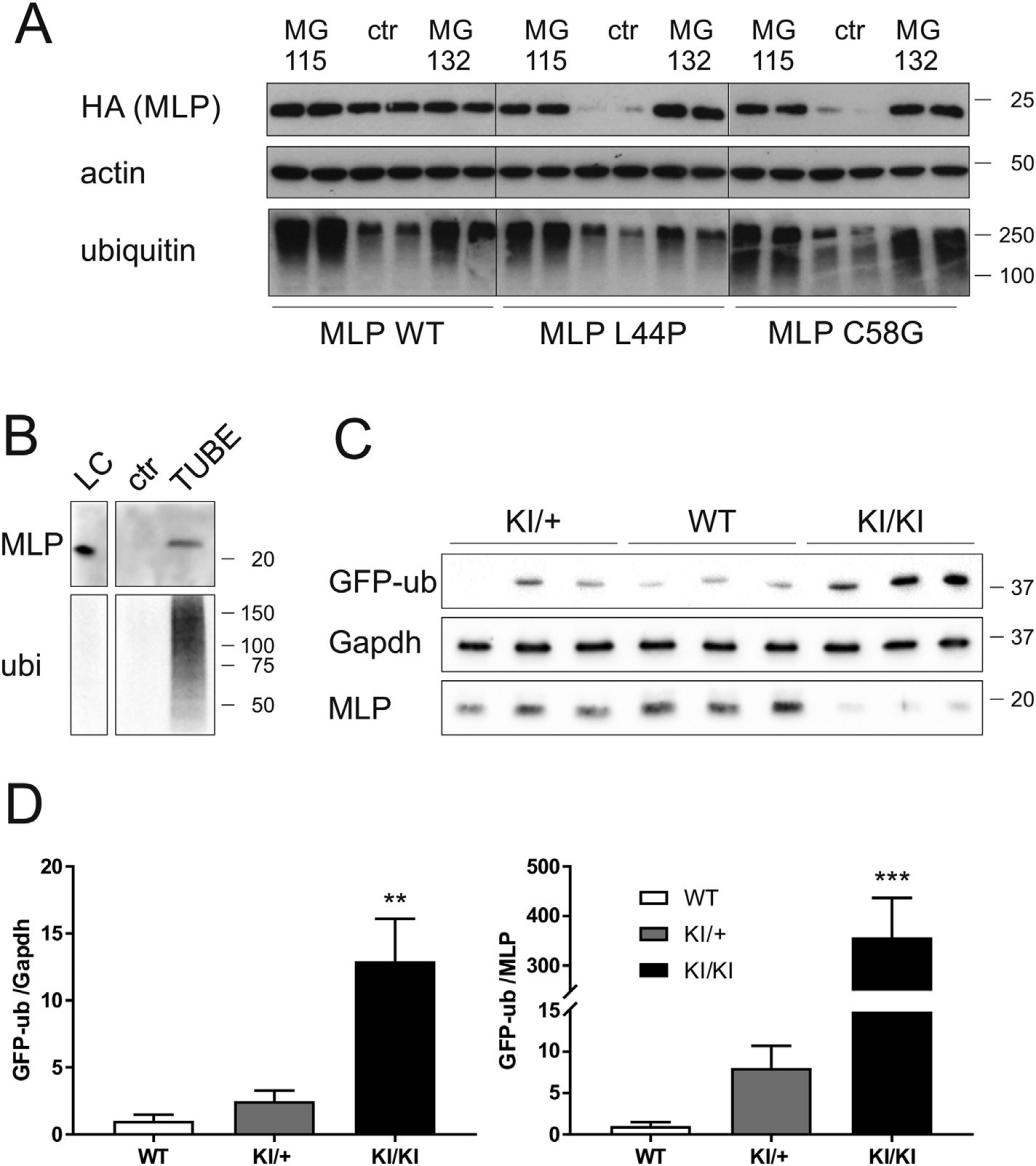
HCM-associated MLP mutant proteins are subjected to depletion by proteasomal pathways: A – NRC were transduced with adenoviral particles as in Fig. 5B and treated with proteasomal inhibitors MG-115 and MG-132. Cell lysates were blotted for HA, detecting the recombinant MLP WT, L44P and C58G. Pan-actin served as loading control and blotting for ubiquitin confirmed efficient inhibition of the proteasome by accumulation of ubiquitinated proteins. In control (ctr, DMSO-treated) cells, MLP L44P and C58G are destabilised, however they are restored to WT protein levels upon proteasomal inhibition. B – TUBE assay: Ubiquitinated proteins were pulled down from *Csrp3* KI/KI heart lysate (treated *in vivo* with proteasomal inhibitor MG-262 1 μmol/kg BW for 20 h) using immobilised tandem ubiquitin-binding entities (TUBE). Agarose matrix without TUBE served as control (ctr). Lysate controls are shown (LC, 1% of input). Blotting for ubiquitin (ubi) indicates the enrichment of ubiquitinated proteins in the TUBE pulldown. Blotting for MLP shows specific pulldown of MLP with TUBE, indicating ubiquitination of the protein. Please note the higher molecular weight (>20 kDa) than normal endogenous MLP (< 20 kDa, see panel C). C – Proteasomal overload in the KI/KI mice: The ubiquitin G76 V GFP reporter (GFP-ub) was crossed onto mice of the three genotypes. Samples of hearts of 6 month old mice were blotted for GFP and increased accumulation of GFP-ub was observed in the KI/KI mice, indicating proteasomal overload. Blotting for MLP illustrates the genotype of the mice and Gapdh served as loading control. The position of marker proteins is indicated (molecular weight in kDa). D – Quantification of GFP-ub induction (blots relating to panel C), expressed relative to Gapdh (left) or MLP (right) protein levels, n = 6 per group. A striking induction of the GFP-ub reporter is observed in KI/KI hearts; **p < 0.01, ***p < 0.001.

Two major pathways are known to control protein degradation in mammalian cells: autophagy and degradation by the UPS (reviewed in [18]). To test the possible role of these mechanisms in depletion of mutant MLP, NRCs were transduced with WT or mutant MLP and treated with inhibitors targeting either autophagy or the UPS. In control treated cells, both MLP L44P and C58G proteins were destabilised, as expected (Fig. 6A, Fig. S8C). Inhibition of autophagy with bafilomycin or LY294002 had no effect on mutant MLP depletion in NRC (Fig. S8C and data not shown), suggesting that autophagy does not mediate mutant MLP depletion. In contrast, inhibition of the UPS by MG-115 or MG-132 treatment fully restored mutant MLP to WT levels (Fig. 6A), indicating that this pathway is indeed responsible for depleting mutant MLP in these cells.

Using tandem ubiquitin-binding entities (TUBEs, [19]), we pulled down MLP C58 from lysates of *Csrp3* KI/KI hearts (Fig. 6B), suggesting that MLP C58G is ubiquitinated *in vivo*. To confirm the role of the UPS in MLP C58G depletion *in vivo*, *Csrp3* KI/+ and KI/KI mice were interbred with a ubiquitin G76V-GFP reporter line [20]. In this line, the ubiquitin-GFP reporter protein is constantly turned over, but accumulates upon inhibition [20] or overload [21, 22] of the UPS. While the ubiquitin-GFP reporter protein was only detectable at low levels in WT and KI/+ mice, it clearly accumulated in KI/KI mice (Fig. 6C, D), suggesting overload of the UPS in this mouse model of non-sarcomeric HCM.

**Fig. 7:**
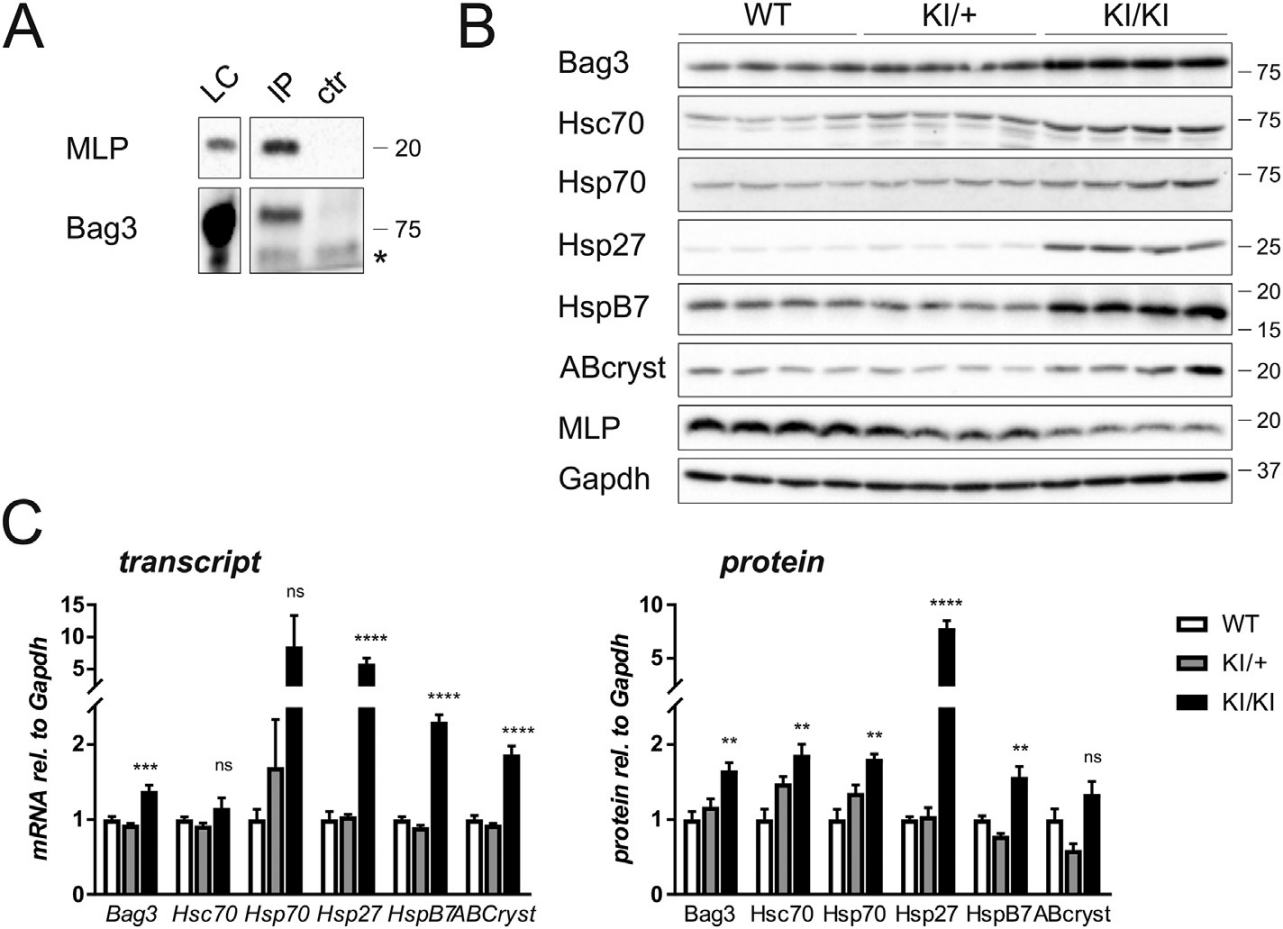
Proteotoxic stress response in the KI/KI mice: A – Co-immunoprecipitation of MLP and Bag3 from KI/KI heart lysate (see Fig. 6B). MLP C58G was precipitated using anti-MLP 79D2 antibody (IP) or isotype antibody control (ctr). Lysate controls are shown as detection controls (LC, 1% of input); * indicates signal from antibody chains. Bag3 co-precipitates with MLP C58G, evidencing a complex formation between the endogenous proteins. The position of marker proteins is indicated (molecular weight in kDa). B – In the KI/KI mice with proteasomal overload, the protein quality control complex Bag3 - Hsp70 – Hsc70 is induced and small heat shock proteins Hsp27, HspB7 and αβ-crystallin (ABcryst) are upregulated. Please note, the blots for MLP and loading control Gapdh on the same membrane are already shown in Fig. 3B. The position of marker proteins is indicated (molecular weight in kDa). C – Left: Assessment of transcriptional changes by qPCR for genes related to protein quality control. All measurements are normalised to *Gapdh* (n = 6 per group); significant changes are observed in the hearts of KI/KI mice for all transcripts apart from Hsc70 and Hsp70. Right: Quantification of protein levels of proteins involved in protein quality control (blots of panel 7B). All measurements are normalised to Gapdh (n = 4 per group); significant changes are observed in the hearts of KI/KI mice for all proteins apart from αβ-crystallin. **p < 0.01, ***p < 0.001, ****p < 0.0001 *versus* WT.

Co-chaperone BCL-2 associated athanogene3 (Bag3) plays important roles in protein quality control [23] and its crucial role in the maintenance of cardiac homeostasis has recently been demonstrated by cardiomyopathy resulting from loss-of-function mutations in both mice and humans [24]. Together with its associated proteins, Hsp70 and Hsc70, Bag3 stabilises small heat shock proteins (Hsps) involved in the refolding or degradation of unfolded protein substrates. Through co-immunoprecipitation studies, we found Bag3 to interact with MLP C58G from *Crsp3* KI/KI hearts (Fig. 7A). This prompted us to interrogate the Bag3 chaperone system both at the transcript and protein level (Fig. 7B, C). We found a striking induction of the Bag3 – Hsp70 – HSc70 complex in the KI/KI mice, together with an upregulation of small Hsps, particularly Hsp27.

Taken together, our mouse model provides insights into the pathological mechanisms of cardiomyopathy in the presence of a non-sarcomeric HCM-causing mutation in *CSRP3*. Furthermore, our work indicates that proteasomal degradation by the UPS is responsible for the depletion of HCM-associated mutant MLP, which is the underlying driver of the disease.

## 3 Discussion

Genetic studies have clearly demonstrated that *CSRP3* mutations can cause autosomal dominant HCM [4, 9, 12], even though the encoded protein, MLP, is not a sarcomeric protein [4] and hence contradicts the existing paradigm of HCM as a “disease of the sarcomere”. Consequently, *CSRP3* mutations, despite their rarity [12], are mechanistically interesting since the mechanisms underlying their pathogenic effects have not been well understood. To this end, we generated a mouse model carrying the best characterised *CSRP3* mutation – C58G [4] – (referred to here as *Csrp3* KI) to study the pathology underlying non-sarcomeric HCM.

The newly generated *Csrp3* KI mouse model reflects aspects of the human cardiomyopathy, especially in the homozygous setting. The mice develop cardiomyopathy with diastolic and systolic impairment, increased LV mass and reduced contractile reserve. At the molecular level, transcriptome profiling shows clear evidence of a pro-fibrotic signature and alterations in agreement with a HCM-like signature. RNAseq findings and qPCR show concordant results with upregulation of key genes involved in hypertrophy and fibrosis such as *Acta1*, *Myh7*, *Tgfb*, *Nppa*, *Nppb*, *Ankrd1/2*. GOrilla and KEGG pathway enrichment findings were consistent with the phenotype of cardiomyopathy in the murine model. GO Enrichment score and a strong evidence of sarcomeric involvement in cellular components, heart rate regulation, and cell proliferation in biological processes emphasize structural and functional changes similar to heart failure.

A targeted analysis of transcripts furthermore demonstrated activation of pro-hypertrophic signalling and re-activation of the fetal gene programme, both hallmarks of HCM. Additionally, changes indicative of cardiac hypertrophy and heart failure, e.g. Fhl1 upregualtion and Fhl2 downregulation [25, 26], Carp induction [27]) and upregulation of β-myosin heavy chain [28] were observed at the protein level in the myocardium of KI/KI mice.

Heterozygous KI/+ mice did not develop an overt cardiac phenotype even upon aging up to 12 months (data not shown). This may be explained by the fact that HCM caused by *CSRP3* mutations is often characterised by late on-set in humans [4]. Furthermore, there are various examples that the direct genetic equivalent of a human HCM mutation does not result in an overt phenotype in mice, as in the case of myosin binding protein C mutations [22] and myosin heavy chain mutations [29, 30]. Fundamental differences in cardiac physiology between mice and humans, such as the different composition of key protein isoforms (e.g. for myosin heavy chain), substantial differences in heart rate and electrical conduction [31, 32], as well as the sedentary life style of laboratory mice may also contribute to this phenomenon. Despite not being significantly affected, KI/+ mice had a tendency towards reduced contractile reserve. Moreover, we demonstrated that the *Csrp3* C58G allele did not functionally replace the WT *Csrp3* allele, when the other *Csrp3* allele was inactivated.

In order to challenge the KI/+ mice, we employed transaortic constriction (TAC) surgery. Surprisingly, their hypertrophic response to aortic banding did not differ to that of their WT littermates, suggesting that MLP's proposed mechano-sensing function [2] is not required for the hypertrophic response to TAC. Nevertheless we cannot rule out a role for MLP in the transition to heart failure, as suggested for a model of myocardial infarction [17].

Our cellular and molecular analyses of this mouse model demonstrated depletion of mutant MLP in both the heterozygous and homozygous C58G KI mice. This is in agreement with the finding of reduced MLP level in the index patient carrying the *CSRP3* C58G mutation [4], and further demonstrated by our cellular experiments, which consistently confirmed protein depletion as a hallmark of all three HCM-causing *CSRP3* mutations investigated. Moreover, we demonstrated ubiquitin conjugation of MLP C58G and that the activity of the UPS is responsible for this protein depletion, while autophagy does not play a direct role. In this respect, HCM-causing *CSRP3* mutations resemble mutations in *MYBPC3*, one of the major sarcomeric disease genes for HCM: the latter are subject to degradation by the UPS *in vitro* [21, 33] and in a mouse model [22]. In the case of both *CSRP3* and *MYBPC3* mutations, the overload of the UPS through the constant turnover of a mutant protein leads to chronic impairment of the system to fulfil its crucial tasks in cellular homeostasis, resulting in accumulation of unfolded proteins. In support of this, we observed the formation of a protein complex of MLP C58G with Bag3 and induction of the Bag3 protein quality control system with upregulation of small Hsps, particularly Hsp27. In the long term, such chronic stressors are thought to contribute to cardiomyopathy [34] and heart failure [35].

The crucial role for Bag3 in cardiac homeostasis is best understood for loss of function mutations in the human *BAG3* gene [36, 37] and inactivation of *Bag3* in mice [24], both resulting in DCM. However, the protein has also been implicated in skeletal muscle disease (myofibrillar myopathy, [38]) and desmin-related cardiomyopathy, e.g. caused by the R120G mutation in *CRYAB* [38]. Overexpression of the latter in a mouse model activates cardiac hypertrophy [39] and Bag3 has been found to regulate contractility and calcium homeostasis in cardiac cells [40]. It is plausible that activation of Bag3 protein complex could contribute to pathogenic activation of hypertrophic signalling cascades also in the *Csrp3* KI/KI mice.

Our recent work has shown that MLP acts as an endogenous inhibitor of PKCα activity in non-failing hearts, potentially by providing an abundant cytoplasmic substrate competing with the activating auto-phosphorylation of the kinase [6]. In pathological settings such as heart failure, PKCα is chronically activated and MLP cannot dampen PKCα activation sufficiently. Simultaneous induction of Carps leads to a recruitment of a complex of Carps and activated PKCα to the intercalated disk. It is speculated that chronic PKCα activity in this compartment is detrimental to the heart, e.g. by affecting adrenergic signalling [41].

In our mouse model, protein depletion through UPS activity results in a lack of functional MLP and we have further shown that this is a feature of all three HCM-causing MLP mutations investigated in our cellular experiments. At the same time, these three HCM mutations were found to be hypo-phosphorylated by PKCα [6]. However, any direct effect of the mutant MLP on PKCα activity is irrelevant as the lack of functional MLP levels through UPS-mediated protein depletion overrides it. As a consequence, PKCα becomes chronically activated (Fig. S9) and may cause aberrant induction of heart failure signalling. As all three HCM mutations are affecting conserved residues in the same region of the protein, namely the second zinc finger of the first LIM domain, it is likely that all three affect the protein structure [42] in a similar way and lead to partial protein unfolding as demonstrated previously for MLP C58G [4, 43]. These unfolded proteins will be recognised by protein quality control systems (as evidenced by the interaction of MLP C58G with Bag3 and the induction of Bag3 and associated heat shock proteins in our mouse model) and subsequently be targeted for degradation by the UPS. The mode of action appears to differ for DCM-associated mutations: K69R and G72R are located in the intrinsically unstructured glycine-rich region [42], hence an unfolded protein response in the presence of the mutations is unlikely. Instead, these mutations affect MLP's ability to inhibit PKC activity [6]. In addition, a role of amino acids 64–69 in nuclear shuttling of MLP [8] and acetylation of K69 [44] are potentially disturbed by both DCM-associated mutations.

In conclusion, our newly generated *Csrp3* KI mouse model of non-sarcomeric HCM, combined with extensive cell-based work, provides important insights into molecular mechanisms underlying pathogenic effects of HCM-associated *CSPR3* mutations.

## Disclosures

The authors have no conflict of interest to declare.

## Appendix A Supplementary data

Supplementary material Image 1

## References

[1] Arber S., Halder G., Caroni P. (1994). Muscle LIM protein, a novel essential regulator of myogenesis, promotes myogenic differentiation. Cell.

[2] Knoll R., Hoshijima M., Hoffman H.M., Person V., Lorenzen-Schmidt I., Bang M.L., Hayashi T., Shiga N., Yasukawa H., Schaper W., McKenna W., Yokoyama M., Schork N.J., Omens J.H., McCulloch A.D., Kimura A., Gregorio C.C., Poller W., Schaper J., Schultheiss H.P., Chien K.R. (2002). The cardiac mechanical stretch sensor machinery involves a Z disc complex that is defective in a subset of human dilated cardiomyopathy. Cell.

[3] Boateng S.Y., Senyo S.E., Qi L., Goldspink P.H., Russell B. (2009). Myocyte remodeling in response to hypertrophic stimuli requires nucleocytoplasmic shuttling of muscle LIM protein. J. Mol. Cell. Cardiol..

[4] Geier C., Gehmlich K., Ehler E., Hassfeld S., Perrot A., Hayess K., Cardim N., Wenzel K., Erdmann B., Krackhardt F., Posch M.G., Osterziel K.J., Bublak A., Nagele H., Scheffold T., Dietz R., Chien K.R., Spuler S., Furst D.O., Nurnberg P., Ozcelik C. (2008). Beyond the sarcomere: CSRP3 mutations cause hypertrophic cardiomyopathy. Hum. Mol. Genet..

[5] Vafiadaki E., Arvanitis D.A., Sanoudou D. (2015). Muscle LIM Protein: master regulator of cardiac and skeletal muscle functions. Gene.

[6] Lange S., Gehmlich K., Lun A.S., Blondelle J., Hooper C., Dalton N.D., Alvarez E.A., Zhang X., Bang M.L., Abassi Y.A., Dos Remedios C.G., Peterson K.L., Chen J., Ehler E. (2016). MLP and CARP are linked to chronic PKCalpha signalling in dilated cardiomyopathy. Nat. Commun..

[7] Arber S., Hunter J.J., Ross J., Hongo M., Sansig G., Borg J., Perriard J.C., Chien K.R., Caroni P. (1997). MLP-deficient mice exhibit a disruption of cardiac cytoarchitectural organization, dilated cardiomyopathy, and heart failure. Cell.

[8] Bos J.M., Poley R.N., Ny M., Tester D.J., Xu X., Vatta M., Towbin J.A., Gersh B.J., Ommen S.R., Ackerman M.J. (2006). Genotype-phenotype relationships involving hypertrophic cardiomyopathy-associated mutations in titin, muscle LIM protein, and telethonin. Mol. Genet. Metab..

[9] Geier C., Perrot A., Ozcelik C., Binner P., Counsell D., Hoffmann K., Pilz B., Martiniak Y., Gehmlich K., van der Ven P.F., Furst D.O., Vornwald A., von Hodenberg E., Nurnberg P., Scheffold T., Dietz R., Osterziel K.J. (2003). Mutations in the human muscle LIM protein gene in families with hypertrophic cardiomyopathy. Circulation.

[10] Hershberger R.E., Parks S.B., Kushner J.D., Li D., Ludwigsen S., Jakobs P., Nauman D., Burgess D., Partain J., Litt M. (2008). Coding sequence mutations identified in MYH7, TNNT2, SCN5A, CSRP3, LBD3, and TCAP from 313 patients with familial or idiopathic dilated cardiomyopathy. Clin. Transl. Sci..

[11] Mohapatra B., Jimenez S., Lin J.H., Bowles K.R., Coveler K.J., Marx J.G., Chrisco M.A., Murphy R.T., Lurie P.R., Schwartz R.J., Elliott P.M., Vatta M., McKenna W., Towbin J.A., Bowles N.E. (2003). Mutations in the muscle LIM protein and alpha-actinin-2 genes in dilated cardiomyopathy and endocardial fibroelastosis. Mol. Genet. Metab..

[12] Walsh R., Buchan R., Wilk A., John S., Felkin L.E., Thomson K.L., Chiaw T.H., Loong C.C., Pua C.J., Raphael C., Prasad S., Barton P.J., Funke B., Watkins H., Ware J.S., Cook S.A. (2017). Defining the genetic architecture of hypertrophic cardiomyopathy: re-evaluating the role of non-sarcomeric genes. Eur. Heart J..

[13] Gehmlich K., Geier C., Milting H., Furst D., Ehler E. (2008). Back to square one: what do we know about the functions of muscle LIM protein in the heart?. J. Muscle Res. Cell Motil..

[14] Zolk O., Caroni P., Bohm M. (2000). Decreased expression of the cardiac LIM domain protein MLP in chronic human heart failure. Circulation.

[15] Lygate C.A., Schneider J.E., Hulbert K., ten Hove M., Sebag-Montefiore L.M., Cassidy P.J., Clarke K., Neubauer S. (2006). Serial high resolution 3D-MRI after aortic banding in mice: band internalization is a source of variability in the hypertrophic response. Basic Res. Cardiol..

[16] Feldman A.M., Weinberg E.O., Ray P.E., Lorell B.H. (1993). Selective changes in cardiac gene expression during compensated hypertrophy and the transition to cardiac decompensation in rats with chronic aortic banding. Circ. Res..

[17] Heineke J., Ruetten H., Willenbockel C., Gross S.C., Naguib M., Schaefer A., Kempf T., Hilfiker-Kleiner D., Caroni P., Kraft T., Kaiser R.A., Molkentin J.D., Drexler H., Wollert K.C. (2005). Attenuation of cardiac remodeling after myocardial infarction by muscle LIM protein-calcineurin signaling at the sarcomeric Z-disc. Proc. Natl. Acad. Sci. U. S. A..

[18] Wang C., Wang X. (2015). The interplay between autophagy and the ubiquitin-proteasome system in cardiac proteotoxicity. Biochim. Biophys. Acta.

[19] Hjerpe R., Aillet F., Lopitz-Otsoa F., Lang V., England P., Rodriguez M.S. (2009). Efficient protection and isolation of ubiquitylated proteins using tandem ubiquitin-binding entities. EMBO Rep..

[20] Lindsten K., Menendez-Benito V., Masucci M.G., Dantuma N.P. (2003). A transgenic mouse model of the ubiquitin/proteasome system. Nat. Biotechnol..

[21] Sarikas A., Carrier L., Schenke C., Doll D., Flavigny J., Lindenberg K.S., Eschenhagen T., Zolk O. (2005). Impairment of the ubiquitin-proteasome system by truncated cardiac myosin binding protein C mutants. Cardiovasc. Res..

[22] Vignier N., Schlossarek S., Fraysse B., Mearini G., Kramer E., Pointu H., Mougenot N., Guiard J., Reimer R., Hohenberg H., Schwartz K., Vernet M., Eschenhagen T., Carrier L. (2009). Nonsense-mediated mRNA decay and ubiquitin-proteasome system regulate cardiac myosin-binding protein C mutant levels in cardiomyopathic mice. Circ. Res..

[23] Behl C. (2016). Breaking BAG: the co-chaperone BAG3 in health and disease. Trends Pharmacol. Sci..

[24] Fang X., Bogomolovas J., Wu T., Zhang W., Liu C., Veevers J., Stroud M.J., Zhang Z., Ma X., Mu Y., Lao D.H., Dalton N.D., Gu Y., Wang C., Wang M., Liang Y., Lange S., Ouyang K., Peterson K.L., Evans S.M., Chen J. (2017). Loss-of-function mutations in co-chaperone BAG3 destabilize small HSPs and cause cardiomyopathy. J. Clin. Invest..

[25] Friedrich F.W., Reischmann S., Schwalm A., Unger A., Ramanujam D., Munch J., Muller O.J., Hengstenberg C., Galve E., Charron P., Linke W.A., Engelhardt S., Patten M., Richard P., van der Velden J., Eschenhagen T., Isnard R., Carrier L. (2014). FHL2 expression and variants in hypertrophic cardiomyopathy. Basic Res. Cardiol..

[26] Lim D.S., Roberts R., Marian A.J. (2001). Expression profiling of cardiac genes in human hypertrophic cardiomyopathy: insight into the pathogenesis of phenotypes. J. Am. Coll. Cardiol..

[27] Zolk O., Frohme M., Maurer A., Kluxen F.W., Hentsch B., Zubakov D., Hoheisel J.D., Zucker I.H., Pepe S., Eschenhagen T. (2002). Cardiac ankyrin repeat protein, a negative regulator of cardiac gene expression, is augmented in human heart failure. Biochem. Biophys. Res. Commun..

[28] Theis J.L., Bos J.M., Theis J.D., Miller D.V., Dearani J.A., Schaff H.V., Gersh B.J., Ommen S.R., Moss R.L., Ackerman M.J. (2009). Expression patterns of cardiac myofilament proteins: genomic and protein analysis of surgical myectomy tissue from patients with obstructive hypertrophic cardiomyopathy. Circ. Heart Fail..

[29] Blankenburg R., Hackert K., Wurster S., Deenen R., Seidman J.G., Seidman C.E., Lohse M.J., Schmitt J.P. (2014). beta-Myosin heavy chain variant Val606Met causes very mild hypertrophic cardiomyopathy in mice, but exacerbates HCM phenotypes in mice carrying other HCM mutations. Circ. Res..

[30] Geisterfer-Lowrance A.A., Christe M., Conner D.A., Ingwall J.S., Schoen F.J., Seidman C.E., Seidman J.G. (1996). A mouse model of familial hypertrophic cardiomyopathy. Science.

[31] Boukens B.J., Rivaud M.R., Rentschler S., Coronel R. (2014). Misinterpretation of the mouse ECG: 'musing the waves of Mus musculus'. J. Physiol..

[32] Milani-Nejad N., Janssen P.M. (2014). Small and large animal models in cardiac contraction research: advantages and disadvantages. Pharmacol. Ther..

[33] Bahrudin U., Morisaki H., Morisaki T., Ninomiya H., Higaki K., Nanba E., Igawa O., Takashima S., Mizuta E., Miake J., Yamamoto Y., Shirayoshi Y., Kitakaze M., Carrier L., Hisatome I. (2008). Ubiquitin-proteasome system impairment caused by a missense cardiac myosin-binding protein C mutation and associated with cardiac dysfunction in hypertrophic cardiomyopathy. J. Mol. Biol..

[34] Schlossarek S., Frey N., Carrier L. (2014). Ubiquitin-proteasome system and hereditary cardiomyopathies. J. Mol. Cell. Cardiol..

[35] Tang M., Li J., Huang W., Su H., Liang Q., Tian Z., Horak K.M., Molkentin J.D., Wang X. (2010). Proteasome functional insufficiency activates the calcineurin-NFAT pathway in cardiomyocytes and promotes maladaptive remodelling of stressed mouse hearts. Cardiovasc. Res..

[36] Rafiq M.A., Chaudhry A., Care M., Spears D.A., Morel C.F., Hamilton R.M. (2017). Whole exome sequencing identified 1 base pair novel deletion in BCL2-associated athanogene 3 (BAG3) gene associated with severe dilated cardiomyopathy (DCM) requiring heart transplant in multiple family members. Am. J. Med. Genet. A.

[37] Toro R., Perez-Serra A., Campuzano O., Moncayo-Arlandi J., Allegue C., Iglesias A., Mangas A., Brugada R. (2016). Familial dilated cardiomyopathy caused by a novel frameshift in the BAG3 gene. PLoS One.

[38] Selcen D., Muntoni F., Burton B.K., Pegoraro E., Sewry C., Bite A.V., Engel A.G. (2009). Mutation in BAG3 causes severe dominant childhood muscular dystrophy. Ann. Neurol..

[39] Wang X., Osinska H., Klevitsky R., Gerdes A.M., Nieman M., Lorenz J., Hewett T., Robbins J. (2001). Expression of R120G-alphaB-crystallin causes aberrant desmin and alphaB-crystallin aggregation and cardiomyopathy in mice. Circ. Res..

[40] Feldman A.M., Gordon J., Wang J., Song J., Zhang X.Q., Myers V.D., Tilley D.G., Gao E., Hoffman N.E., Tomar D., Madesh M., Rabinowitz J., Koch W.J., Su F., Khalili K., Cheung J.Y. (2016). BAG3 regulates contractility and Ca(2+) homeostasis in adult mouse ventricular myocytes. J. Mol. Cell. Cardiol..

[41] Liu Q., Molkentin J.D. (2011). Protein kinase Calpha as a heart failure therapeutic target. J. Mol. Cell. Cardiol..

[42] Schallus T., Feher K., Ulrich A.S., Stier G., Muhle-Goll C. (2009). Structure and dynamics of the human muscle LIM protein. FEBS Lett..

[43] Gehmlich K., Geier C., Osterziel K.J., Van der Ven P.F., Furst D.O. (2004). Decreased interactions of mutant muscle LIM protein (MLP) with N-RAP and alpha-actinin and their implication for hypertrophic cardiomyopathy. Cell Tissue Res..

[44] Gupta M.P., Samant S.A., Smith S.H., Shroff S.G. (2008). HDAC4 and PCAF bind to cardiac sarcomeres and play a role in regulating myofilament contractile activity. J. Biol. Chem..

