# Supplementary material for: Mutant Muscle LIM Protein C58G causes cardiomyopathy through protein depletion
Source: J Mol Cell Cardiol. 2018 Aug;121:287–96. doi: 10.1016/j.yjmcc.2018.07.248 (PMC6117453; doi:10.1016/j.yjmcc.2018.07.248)
Supplement: Supplementary file 1 — Supplementary material [file mmc1.pdf]

Mutant Muscle LIM Protein C58G causes cardiomyopathy through protein depletion

Mehroz Ehsan[a,*], Matthew Kelly[a,b,*], Charlotte Hooper[a], Arash Yavari[a,b,c], Julia Beglov[a,b], Mohamed Bellahcene[a,b,1], Kirandeep Ghataorhe[a], Giulia Poloni[a], Anuj Goel[a,b], Theodosios Kyriakou[a,b], Karin Fleischanderl[d], Elisabeth Ehler[d], Eugene Makeyev[e], Stephan Lange[f], Houman Ashrafian[a,b,c], Charles Redwood[a], Benjamin Davies[g], Hugh Watkins[a,b], Katja Gehmlich[a,b,#]

Supplemental Material

1. Material and Methods

1.1. Ethical statement

Experimental procedures were performed in accordance with the UK Home office guidelines (project licences 30/2444 and 30/2977) and approved by respective institutional review boards.Animals were housed in specific pathogen free conditions, with the only reported positives on health screening over the entire time course of these studies being for *Tritrichomonas sp* and *Entamoeba spp.* All animals were housed in social groups, provided with food and water ad-libitum and, maintained on a 12h light:12h dark cycle (150–200 lux cool white LED light, measured at the cage floor). Phenotyping experiments were blinded or randomized and no animals were excluded from the study.

1.2. Generation of mice

A targeting vector, suitable for the generation of a *Csrp3* knock-out first allele (*Csrp3*$^{tm1a(EUCOMM)Hmgu}$), was ordered from the IKMC (PG00247_Z_6_C03). This targeting vector is suitable for the integration of loxP sites flanking *Csrp3* exon 3 (ENSMUSE00000199344) together with an FRT flanked reporter/selection cassette (Fig. S1A). The C58G mutation was introduced by sub-cloning a PacI – NotI fragment (7 kb) into pGEMT (Promega) and performing site-directed mutagenesis (Agilent Quikchange XL-II)

using the following primers: 5’-gagtcagagatctacGgtaaggtgtgc-3’ and 5’-gcacaccttacCgtagatctctgactc-3’. The mutated PacI – NotI fragment was re-ligated into the targeting vector. The completed targeting vector was linearised with AsiSI and electroporated into C57BL/6N JM8F6 embryonic stem cells (Biorad, 700V, 3uF). Following selection in 210 μg/ml G418, recombinant clones were screened by PCR to detect homologous recombination over the 3' arm. A forward primer (5'-CACACCTCCCCCTGAACCTGAAAC-3') binding within the FRT flanked reporter/selection cassette was used together with a reverse primer (5'- CTTGTTACTTCGGTGCAGCCTATG-3') binding downstream of the 3' homology arm to amplify a 6.0 kb fragment from correctly recombined clones. Positive clones were examined for correct recombination at the 5' end by long range PCR using a forward primer (5'- CTCCAACTCTTGGCGTAAGCATCAC-3') binding upstream of the 5' homology arm and a reverse primer (5'-CACAACGGGTTCTTCTGTTAGTCC-3') binding within the FRT flanked reporter/selection cassette. Correctly targeted clones yielded a 7.6 kb amplicon. Sanger sequence analysis of the 6.0 kb amplicon encompassing the 3' homology arm with primer 5'-GGCGAGCAGGTCCAAGACTAC -3' was used to determine whether the C58G mutation was present.

ES cells from correctly targeted clones were injected into albino C57BL/6J blastocysts and the resulting chimeras were mated with albino C57BL/6J females. Successful germline transmission yielded black pups and F1 mice harbouring the targeted *Csrp3* allele were identified by PCR (using primer pair 5’-ATAGGAACTTCGTCGAGATAACTT -3’ and 5’- GTGGGACTTTCAAGGAGGTAA -3’). F1 heterozygous male mice were bred with C57BL/6J Flp recombinase deleter mice (Tg(ACTB-Flpe)9205Dym (Jax stock 005703) and offspring were screened for the deletion of the selection cassette using a forward primer (5'- TGAGCTGTTCAGGGAGGCTA -3') binding immediately upstream of the cassette and a reverse primer (5'-GGTGGGACTTTCAAGGAGGT-3') binding immediately downstream of the cassette. A 374 bp amplicon was obtained from the Flp deleted humanized allele and a 201 bp amplicon was

obtained from the wild-type allele. Heterozygous mice without the reporter/selection cassette were then backcrossed with C57BL/6J to remove the Flp transgene prior to inter-crossing to obtain experimental cohorts of heterozygous, homozygous and wild-type mice which were genotyped with the above PCR (Fig. S1B).

Ubiquitin G76V-GFP reporter mice (Jax stock 008111, [1]) and C57BL/6J mice were obtained from Charles River and genotyped with the following primer pair: 5'-TCGACCAAGCTTCCCCACCAC-3' and 5'-CCTACAGCTCCTGGGCAACGT-3'. MLP knockout mice [2] were backcrossed with C57BL/6J for > 6 generations before generating heterozygous MLP knockout mice or crossing them with *Csrp3* knock-in mice. Genotyping for the knock-out allele was performed as above using the following primer pair: 5'-CCTTCTATCGCCTTCTTGACGAG-3' and 5'- CTCATACTCGGAACTTGGG-3', the wild-type allele was probed for with the following primer pair: 5'-CAGGCTGTCCCCTAGACCTC-3' and 5' –GAACCACCAACAGACAGTAGTAGG-3'. All genotyping was performed from ear biopsies with REDExtract-N-Amp™ Tissue PCR Kit (Sigma).

1.3. *In vivo* phenotyping

All phenotyping was performed on male mice (young adult, i.e. 2-3 months old unless stated otherwise); littermates and age-matched male C57BL/6J (Charles River) served as controls. Echocardiography was performed under light general anaesthesia (1-1.5 % isoflurane) with a 22 to 55 MHz linear array transducer using the Vevo 2100 ultrasound system (Visualsonics). For left ventricular (LV) haemodynamics, mice were anaesthetised with isoflurane (1.25–1.5%) for the measurement of haemodynamic indices using a 1.4 F Millar Mikro-tip catheter (SPR-671) inserted into the LV via the carotid artery. After 15 min of baseline recordings, contractile reserve was assessed by infusion of dobutamine via the jugular vein (4–16 ng per g bodyweight per min). For details see [3].
Transaortic constriction (TAC) surgery was performed as described [4] on male mice of 2-3 months age. The hypertrophic response was assessed by echocardiography and *ex vivo* studies two weeks after the intervention.

1.4. *Ex vivo* studies

Tibial length measurements, mRNA isolation from ventricular tissue, reverse transcriptase quantitative PCR (qPCR) and Western blotting of mouse hearts were performed on 2-3 months old mice as described [3] using the Taqman probes (Applied Biosystems) and antibodies listed in Table S7).

Histology on paraffin embedded samples (7 µm sections) was performed with hematoxylin and eosin or with Sirius red, using standard protocols.

To confirm the presence of the C58G mutation at transcript level, the relevant portion of *Csrp3* was amplified by PCR from cDNA of a KI/+ heart using the following primers: 5’-ATGCCAAACTGGGGTGGAGG-3’ and 5’- CTCCTTCTTTTCCACTTGCTGTG-3’ and the product subjected to Sanger sequencing. cDNA where no reverse transcriptase had been added served as control. To confirm the mutation at protein level, a protein sample of KI/+ heart was run on an 11 % polyacrylamide gel (Biorad) and stained with Coomassie Brilliant Blue. In gel tryptic digest of bands from 18 to 25 kDa and analysis by mass spectrometry were performed as described [10].

1.5. RNA sequencing

LV tissue samples were powdered by freezing with liquid nitrogen and smashed in a Biopulveriser (Stratech Scientific). 1 ml TRIzol Reagent (Invitrogen) was added to the tissue powder, mixed well and incubated at RT for 2-3 min. 200 µl Chloroform was added and the tube mixed for 20 sec manually and spun at 15,000 g for 4 min at RT. The aqueous phase was transferred to a fresh tube and 500 µl of a 1:1 mixture of acid phenol chloroform were added, mixed for 20 sec and spun as before. The aqueous phase was again transferred to a fresh tube and 500 µl Isopropanol was added and mixed. Samples were incubated for 20 min at -20°C and spun at 4°C for 10 min at maximum speed. The supernatant was removed and 1 ml of 70% ethanol was added and spun for 1 min at 15,000 g. Ethanol was removed and the pellet was rehydrated in an appropriate volume of RNase-free water. Samples were

purified and DNase treated according to the manufacturer's protocol for the PureLink RNA Mini Kit (Ambion by life technologies).

HiSeq 50 Cycle single-read sequencing was performed by the Huntsman Cancer Institute (University of Utah, US) on an Illumina HiSeq 2500 instrument.

RNA-seq libraries were prepared using Illumina TruSeq Stranded mRNA Sample Prep with poly(A) selection and analysed by HiSeq 50 Cycle single-read sequencing using an Illumina HiSeq 2500 instrument. RNA-seq reads were aligned to *Mus musculus* reference genome (GRCm38) using HISAT2 [5]. Alignment files were sorted and indexed with SAMtools [6]. Differential expression analyses were performed using DESeq2 package in R, according to the standard DESeq2 tutorial [7]. Genes were identified to be differentially expressed if they met the following 3 conditions (i) normalized read count > 2 copies/million reads in the RNA-seq library, (ii) log2 fold-change > 1 (upregulated) or < –1 (downregulated), and (iii) $p < 0.001$ for the comparison of normalized read counts between KI/KI and WT animals. The gene list was then functionally annotated using 2 platforms: GOrilla and Gene Set Enrichment Analysis (GSEA).

Gene Ontology (GO) analysis were performed using GOrilla [8] (http://cbl-gorilla.cs.technion.ac.il) on the differentially expressed genes. This method determines whether the number of differentially expressed genes having a particular GO assignment is significantly greater than would be expected by chance, given the total number of genes, the total number of genes having that assignment, and the number of statistically significant genes, overall. We ran GOrilla on a ranked list (by p-value). GO categories with p-values <0.001 were considered significant.

Gene Set enrichment analysis (GSEA) was performed on differentially expressed genes on gene pathways defined by the KEGG database [9], downloaded from MiSigDB (http://www.broadinstitute.org/gsea/msigdb/). GSEA is used to determine whether a predefined gene set shows statistically a significant difference between two biological states among a list of gene sets. We used phenotype permutation for 1000 times, and chose the

weighted signal to noise statistical approach to rank genes and complete the GSEA analysis. Only the pathways with a false discovery rate < 0.001 are reported.

1.6. Virus design and production

cDNAs coding for human MLP WT, L44P, C58G, S54R/E55G, K69R with an N-terminal –YPYDVPDYA - HA Tag were cloned in to a shuttle vector (pShuttle-IRES-HrGFP1, Agilent), containing a CMV promoter 3' of the multiple cloning site and an inter-ribosomal expression site linked to a hrGFP gene at the 5' end. Recombinant adenoviral constructs were created using the AdEasy recombination system (Agilent technologies) and purified via CsCl gradient centrifugation as previously described [10]. Particles were desalted into a buffer containing 20 mM Tris-HCl pH 7.0 and 7.5 % sucrose. Desalted virus was mixed 1:1 with a solution containing 20 % glycerol 80 % FBS for cryo- protection and stored at -80 °C. Viral titre estimation was carried out estimating the number of green cells infected by serial dilutions of purified virus in a plate containing ~$2x10^6$ unmodified HEK293 cells over a 48 hour period. Estimates showed that the number of viable virus particles was between $1x10^{10}$ and $7x10^{11}$ mL^{-1}.

1.7. Cellular experiments

Adult mouse cardiomyocytes were isolated and immunofluorescence performed as described [11, 12] using anti-MLP 79D2 and titin m8 primary antibodies (Table S7).
HEK293 cells were grown in DMEM supplemented with 10 % fetal bovine serum, 1 % penicillin/streptomycin and 1 % L-glutamine (all Sigma) and transfected with lipofectamine 2000 (Thermo Fisher) according to the manufacturer's instructions. 1 µg of plasmid DNA was used per 35 mm dish and cells were harvested 48 hrs post-transfection.
The isolation, culture and adenoviral infection of neonatal rat cardiomyocytes (NRC) was performed as described [12]. MG132, MG115, bafilomycin and LY294002 were purchased from Sigma and dissolved in dimethyl sulfoxide (DMSO). Cells were treated at 5 µM for 8 hrs

(MG-115, MG-132), 50 µM 8 hrs (bafilomycin) and 10 µM 24 hrs (LY294002). Vehicle-treated cells treated for the same length served as controls.

Isolation of Guinea pig left ventricular cardiomyocytes was performed as described [13]. Recombinant adenovirus was immediately added to 3 ml of cell suspension to an estimated multiplicity of infection (MOI) of ~1000, infected cells were placed at 37 °C in a 5 % CO2 atmosphere for 48 hours on laminin-coated coverslips. Immunofluorescence was performed using anti-HA, anti-hrGFP and anti-α-actinin primary antibodies.

1.8. Binding experiments

Csrp3 KI/KI mic were injected intraperitoneally with MG-262 (1 µmol/kg body weight, Bio-Techne) 20 hrs before harvest. Heart tissue was flushed in PBS and snap frozen in liquid nitrogen. Tissue powder was prepared in liquid nitrogen (BioPulverizer, BioSpecs).

For immunoprecipitation, cell lysates were prepared as described [14] using following buffer 1% Triton X-100, 20 mM Tris-HCl (pH 7.6), 138 mM sodium chloride, 5 mM dithiothreitol, 5 % glycerol, phosphatase and protease inhibitors (Roche). Lysates (200 ug protein) were precipitated with 2 µg anti-MLP antibody 79D2 in 500 µL buffer overnight on ice. As control, 2 µg isotype control antibody were used (Sigma, IgG2a). 25 µL Protein G Dynabeads (Thermo Fisher) were added, incubated for 1 hour at 4 degrees (shaking), followed by 15 min at room temperature (shaking). Beads were washed with buffer and bound proteins eluted with 25 µL of SDS sample buffer and subsequent heating (100 degrees 3 min). Western blots were performed as described [14], using VeriBlot (abcam, 1:1000) as secondary antibody.

For tandem ubiquitin-binding entities (TUBE) assays [15], lysis was performed in the buffer above, supplemented with 100 mM N-ethylmaleimide (Sigma) and 50 µM PR-619 (LifeSensors via Tebu-Bio). Lysates (250 ug protein) were incubated with 20 µL TUBE1 agarose (LifeSensors via Tebu-Bio) in 500 µL buffer overnight at 4 degrees (shaking). As control, the same agarose matrix without TUBEs was used (LifeSensors via Tebu-Bio). Next day, the beads were washed 3 times with 1 mL buffer, bound proteins eluted 30 µL of SDS

sample buffer and subsequent heating (100 degrees 3 min) and analysed by Western blotting.

1.9. Statistics

Values are given as mean ± standard error of mean. A one-way ANOVA test followed by Bonferroni post-hoc test was used to compare differences among multiple groups, i.e. WT, KI/+ and KI/KI. Two-way ANOVA test was performed for invasive haemodynamic measurements and TAC experiments. To compare two unpaired sample groups, Student's t-test was performed. $p < 0.05$ was considered significant. Statistical analysis was performed using GraphPad Prism 7.03.

Annotations used: n.s. – not significant, * $p < 0.05$, ** $p < 0.01$, *** $p < 0.001$, *** $p < 0.0001$ versus WT; n indicates number of animals in each group.

2. Supplemental Figures and Tables

Figure S1: A – Strategy to generate the *Csrp3* KI mouse model. An existing EUCOMM targeting vector was modified by introducing the C58G mutation into exon 3 and subsequently used for homologous recombination in ES cells. Mice with the targeted allele, obtained after blastocyst injection of the targeted ES cells, were crossed with FLPe transgenic mice to delete the cassette between the FRT sites. B – The size difference (172 bp) due to the remaining sequence from the targeting vector (containing the FRT and loxP sites) is used for genotyping: the targeted allele yields a PCR product of 373 bp, the WT allele a PCR product of 201 bp. The positions of marker bands are indicated.

Figure S2: A – Confirmation of mutant expression at transcript level: Left – reverse transcriptase PCR was performed on mRNA isolated from KI/+ hearts, a PCR product is only obtained in the presence of reverse transcriptase (RT) enzyme. The positions of marker bands are indicated. Right – Sanger sequencing trace of this PCR product shows the presence of both WT and mutant allele (arrow). B – Identification of MLP C58G protein in KI/+ hearts by mass spectrometry: peptides containing WT sequence as well as peptides containing the C58G mutation (arrow) were identified based on their molecular weight. C – Direct sequencing of peptides by MS/MS shows the presence of the C58G mutation in two peptides (arrows).

Figure S3 – TAC response of KI/+ hearts is identical to that of WT littermates. A – 2 week TAC intervention causes an identical hypertrophic response in both WT and KI/+ hearts (* $p < 0.05$, ** $p < 0.01$ versus sham group of the same genotype). B – Assessment of transcriptional changes by qPCR for genes related to the fetal gene programme (top) and to hypertrophic signalling (bottom) upon TAC. All measurements are normalised to *Gapdh*; there are no significant changes between the TAC response of WT and KI/+ hearts (n = 6 for WT groups, n= 5 for KI/+ groups). C – TAC response of Fhl1 and MLP is similar for WT and KI/+ hearts. Gapdh serves as loading control (n = 3 per group).

Figure S4 – GO term enrichment analysis of genes with significant abundance changes. Significant differentially regulated genes ($p < 0.001$, log fold change >1 or <-1, >2 RPKM) were taken as input for the GOrilla web-server. Top panel shows functional enrichment map for (a) GO Component and (b) GO Process. The colour of each box represents associated p-value. Bottom panel highlights the enrichment scores of statistically significant GO terms.

Figure S5: Further molecular characterisation of the KI/+ and KI/KI mice. A – Assessment of transcriptional changes by qPCR for genes related to apoptosis. Measurements are normalised to *Gapdh*; a 3-fold up-regulation is observed for *Bcl2* in KI/KI hearts; **** $p < 0.0001$, n = 6 per group. B – Quantification of Fhl2 protein down-regulation (see Fig. 2D), normalised to Gapdh, n = 6 per group. An approx. 50 % reduction of Fhl2 protein is observed in KI/KI hearts; ** $p < 0.01$, n = 6 per group. C – Expression of β-myosin heavy chain as a marker of heart failure in KI/KI hearts analysed by Western blotting. β-myosin heavy chain is profoundly upregulated in KI/KI hearts. For reference, pan-myosin is visualised by PonceauS staining and Gapdh serves as loading control.

Figure S6: Histology of cardiac sections from WT, KI/+ and KI/KI mice. H&E (top) as well as Sirus Red (bottom) staining are shown. H&E stain was normal in all three genotypes and mild interstitial fibrosis is evident as red signal in the KI/KI heart (Sirus Red stain). Scale bars represent 50 µm.

Figure S7: *Csrp3* +/- mice have no phenotype at 3 months of age. A - Measurement of *Csrp3* transcript level by qPCR (left) and MLP protein by Western blotting (right and bottom) in *Csrp3* +/- (HET) and WT littermates; qPCR values are normalised to *Gapdh*. For Western blotting, α-actinin was used as loading control and values are normalised to it. In the *Csrp3* +/- mice (HET), *Csrp3* transcript and MLP protein level are down-regulated to 50 % of WT levels; *** $p < 0.001$. B – Normal cardiac dimension and function by echocardiography:

Fractional shortening and end-diastolic dimensions are shown normal in *Csrp3* +/- (HET) mice. For cohort characteristics and a wider set of echocardiographic parameters please refer to Table S5. C – Heart weight is normal in *Csrp3 +/-* mice (HET), values are normalised to tibia length. D – *Csrp3 +/-* mice (HET) show no induction of the fetal gene programme. Transcripts linked to the fetal gene programme were assessed by qPCR (see Fig. 2C) and no changes were observed. As an example, the measurement of *Nppb* is shown (normalised to *Gapdh*). n numbers for each group for each experiment are indicated.

Figure S8: A – Blots corresponding to the experiment in Figure 5A. HEK cells expressing recombinant HA-tagged MLP (WT and mutants, or empty vector) and hrGFP reporter were blotted with anti-HA, anti MLP (79D2) and hrGFP. B – Protein depletion of MLP mutants in adult Guinea pig cardiomyocytes. Cells were transduced with adenovirus delivering the constructs as in panel A (WT, L44P and C58G only), fixed and stained with anti-HA to visualise recombinant MLP (top row). hrGFP (second row) identified transduced cells. Sarcomeres were visualised by staining against α-actinin (third row) and merged images are shown in the bottom row (HA in red, hrGFP in green, α-actinin in blue). Scale bar represents 10 microns. HA-signal is detectable diffusely in the cytoplasm only for MLP WT: MLP L44P and C58G proteins are barely detectable by HA-staining. C – NRC were transduced with adenoviral particles as in Fig. 5B and treated with autophagy inhibitor bafilomycin A1 (baf). Cell lysates were blotted for HA, detecting the recombinant MLP WT, L44P and C58G. Pan-actin served as loading control and blotting for LC3 confirmed efficient inhibition of the autophagy by accumulation of lipidated LC3-II (position indicated). In control cells (ctr, DMSO-treated), MLP L44P and C58G are destabilised. Bafilomycin treatment had no effect, suggesting that autophagy is not involved in MLP depletion in the presence of the mutations.

Figure S9: Up-regulation and activation of PKCα in KI/KI hearts: blotting revealed increased protein levels for PKCα and increased activation (as indicated by phosphorylation of T638, pPKCα) in KI/KI heart samples. Gapdh served as loading control.

Table S1: Echocardiographic parameters of WT, KI/+ and KI/KI hearts.

Table S2: Haemodynamic parameters of WT, KI/+ and KI/KI hearts.

Table S3: Echocardiographic parameters of WT and KI/+ mice sham and TAC-operated.

Table S4: Differentially expressed genes ($p < 0.001$, lfc >1, <-1, >2 RPKM) in KI/KI hearts compared to WT hearts. Genes are sorted by adjusted p-value. Log2foldchange shows values for log (base 2) of fold change (KI Avg/WT Avg), lfcSE (log fold change standard error of mean).

Table S5: Echocardiographic parameters of *Csrp3* +/+ (WT) and *Csrp3* +/- (HET) hearts.

Table S6: Echocardiographic parameters of *Csrp3* +/-, *Csrp3* KI/- and *Csrp3* -/- hearts.

Table S7: List of reagents (TaqMan probes, antibodies) used.

For Tables S1, S3 and S6, heart weight normalised to tibial length (HW/TL) is also given.

3. References

[1] K. Lindsten, V. Menendez-Benito, M.G. Masucci, N.P. Dantuma, A transgenic mouse model of the ubiquitin/proteasome system, Nat Biotechnol 21(8) (2003) 897-902.

[2] S. Arber, J.J. Hunter, J. Ross, Jr., M. Hongo, G. Sansig, J. Borg, J.C. Perriard, K.R. Chien, P. Caroni, MLP-deficient mice exhibit a disruption of cardiac cytoarchitectural organization, dilated cardiomyopathy, and heart failure, Cell 88(3) (1997) 393-403.

[3] K. Gehmlich, M.S. Dodd, J.W. Allwood, M. Kelly, M. Bellahcene, H.V. Lad, A. Stockenhuber, C. Hooper, H. Ashrafian, C.S. Redwood, L. Carrier, W.B. Dunn, Changes in the cardiac metabolome caused by perhexiline treatment in a mouse model of hypertrophic cardiomyopathy, Mol Biosyst 11(2) (2015) 564-73.

[4] C.A. Lygate, J.E. Schneider, K. Hulbert, M. ten Hove, L.M. Sebag-Montefiore, P.J. Cassidy, K. Clarke, S. Neubauer, Serial high resolution 3D-MRI after aortic banding in mice: band internalization is a source of variability in the hypertrophic response, Basic Res Cardiol 101(1) (2006) 8-16.

[5] D. Kim, B. Langmead, S.L. Salzberg, HISAT: a fast spliced aligner with low memory requirements, Nat Methods 12(4) (2015) 357-60.

[6] H. Li, B. Handsaker, A. Wysoker, T. Fennell, J. Ruan, N. Homer, G. Marth, G. Abecasis, R. Durbin, S. Genome Project Data Processing, The Sequence Alignment/Map format and SAMtools, Bioinformatics 25(16) (2009) 2078-9.

[7] M.I. Love, W. Huber, S. Anders, Moderated estimation of fold change and dispersion for RNA-seq data with DESeq2, Genome Biol 15(12) (2014) 550.

[8] E. Eden, R. Navon, I. Steinfeld, D. Lipson, Z. Yakhini, GOrilla: a tool for discovery and visualization of enriched GO terms in ranked gene lists, BMC Bioinformatics 10 (2009) 48.

[9] M. Kanehisa, S. Goto, KEGG: kyoto encyclopedia of genes and genomes, Nucleic Acids Res 28(1) (2000) 27-30.

[10] J. Luo, Z.L. Deng, X. Luo, N. Tang, W.X. Song, J. Chen, K.A. Sharff, H.H. Luu, R.C. Haydon, K.W. Kinzler, B. Vogelstein, T.C. He, A protocol for rapid generation of recombinant adenoviruses using the AdEasy system, Nat Protoc 2(5) (2007) 1236-47.

[11] R. Carnicer, A.B. Hale, S. Suffredini, X. Liu, S. Reilly, M.H. Zhang, N.C. Surdo, J.K. Bendall, M.J. Crabtree, G.B. Lim, N.J. Alp, K.M. Channon, B. Casadei, Cardiomyocyte GTP

cyclohydrolase 1 and tetrahydrobiopterin increase NOS1 activity and accelerate myocardial relaxation, Circ Res 111(6) (2012) 718-27.

[12] R. Hastings, C.P. de Villiers, C. Hooper, L. Ormondroyd, A. Pagnamenta, S. Lise, S. Salatino, S.J. Knight, J.C. Taylor, K.L. Thomson, L. Arnold, S.D. Chatziefthimiou, P.V. Konarev, M. Wilmanns, E. Ehler, A. Ghisleni, M. Gautel, E. Blair, H. Watkins, K. Gehmlich, Combination of Whole Genome Sequencing, Linkage, and Functional Studies Implicates a Missense Mutation in Titin as a Cause of Autosomal Dominant Cardiomyopathy With Features of Left Ventricular Noncompaction, Circ Cardiovasc Genet 9(5) (2016) 426-435.

[13] P. Robinson, X. Liu, A. Sparrow, S. Patel, Y.H. Zhang, B. Casadei, H. Watkins, C.S. Redwood, Hypertrophic cardiomyopathy mutations increase myofilament Ca(2+) buffering, alter intracellular Ca(2+) handling and stimulate Ca(2+) dependent signalling, J Biol Chem (2018).

[14] K. Gehmlich, N. Pinotsis, K. Hayess, P.F. van der Ven, H. Milting, A. El Banayosy, R. Korfer, M. Wilmanns, E. Ehler, D.O. Furst, Paxillin and ponsin interact in nascent costameres of muscle cells, J Mol Biol 369(3) (2007) 665-82.

[15] R. Hjerpe, F. Aillet, F. Lopitz-Otsoa, V. Lang, P. England, M.S. Rodriguez, Efficient protection and isolation of ubiquitylated proteins using tandem ubiquitin-binding entities, EMBO Rep 10(11) (2009) 1250-8.

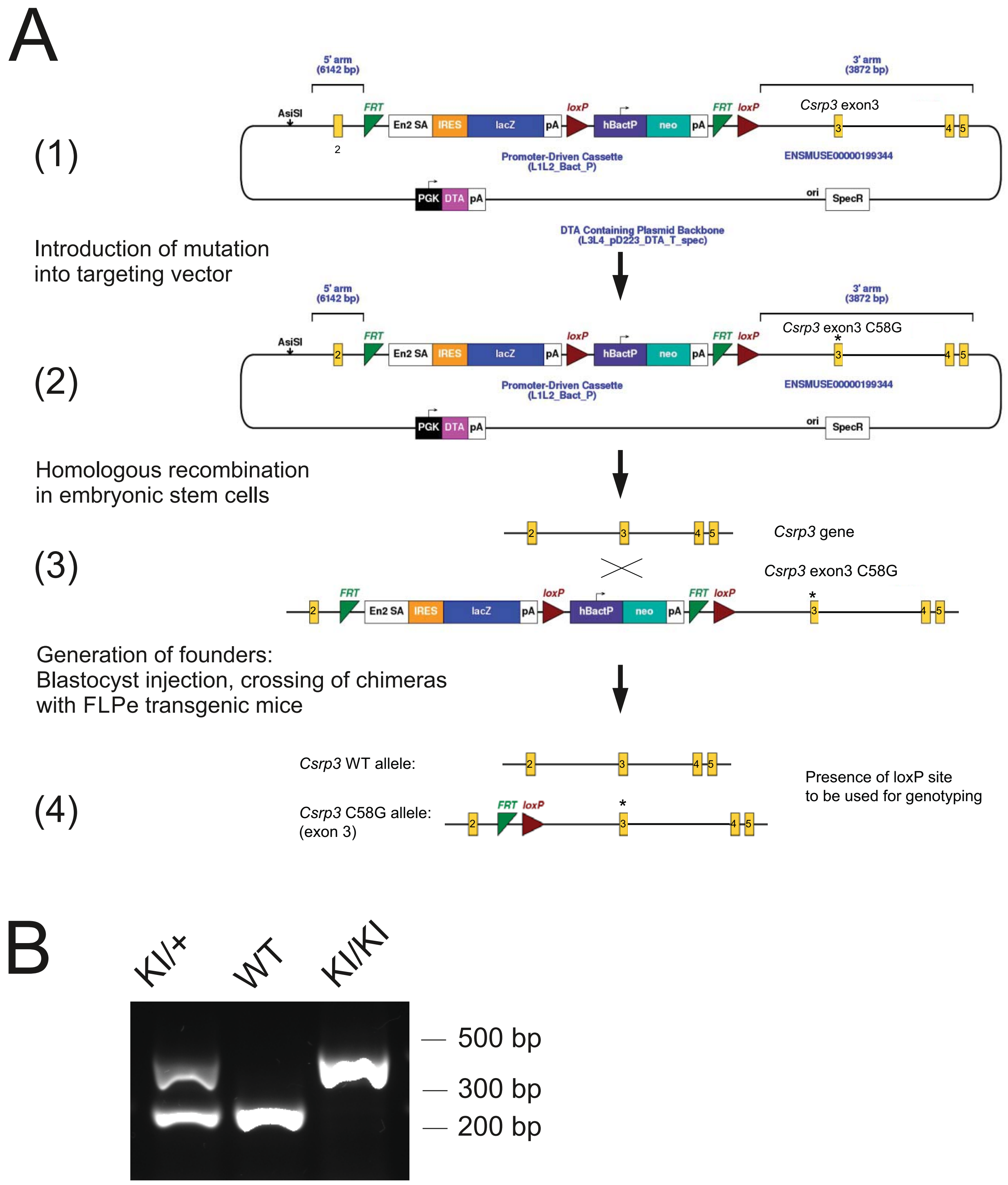

A
(1)
5' arm
(6142 bp)
3' arm
(3872 bp)
AsiSI
FRT
En2 SA
IRES
lacZ
pA
loxP
hBactP
neo
pA
FRT
loxP
Csrp3 exon3
Promoter-Driven Cassette
(L1L2_Bact_P)
ENSMUSE00000199344
PGK
DTA
pA
ori
SpecR
DTA Containing Plasmid Backbone
(L3L4_pD223_DTA_T_spec)
Introduction of mutation
into targeting vector
(2)
Csrp3 exon3 C58G
Homologous recombination
in embryonic stem cells
(3)
Csrp3 gene
Csrp3 exon3 C58G
Generation of founders:
Blastocyst injection, crossing of chimeras
with FLPe transgenic mice
(4)
Csrp3 WT allele:
Csrp3 C58G allele:
(exon 3)
Presence of loxP site
to be used for genotyping
B
KI/+
WT
KI/KI
500 bp
300 bp
200 bp

Figure S1

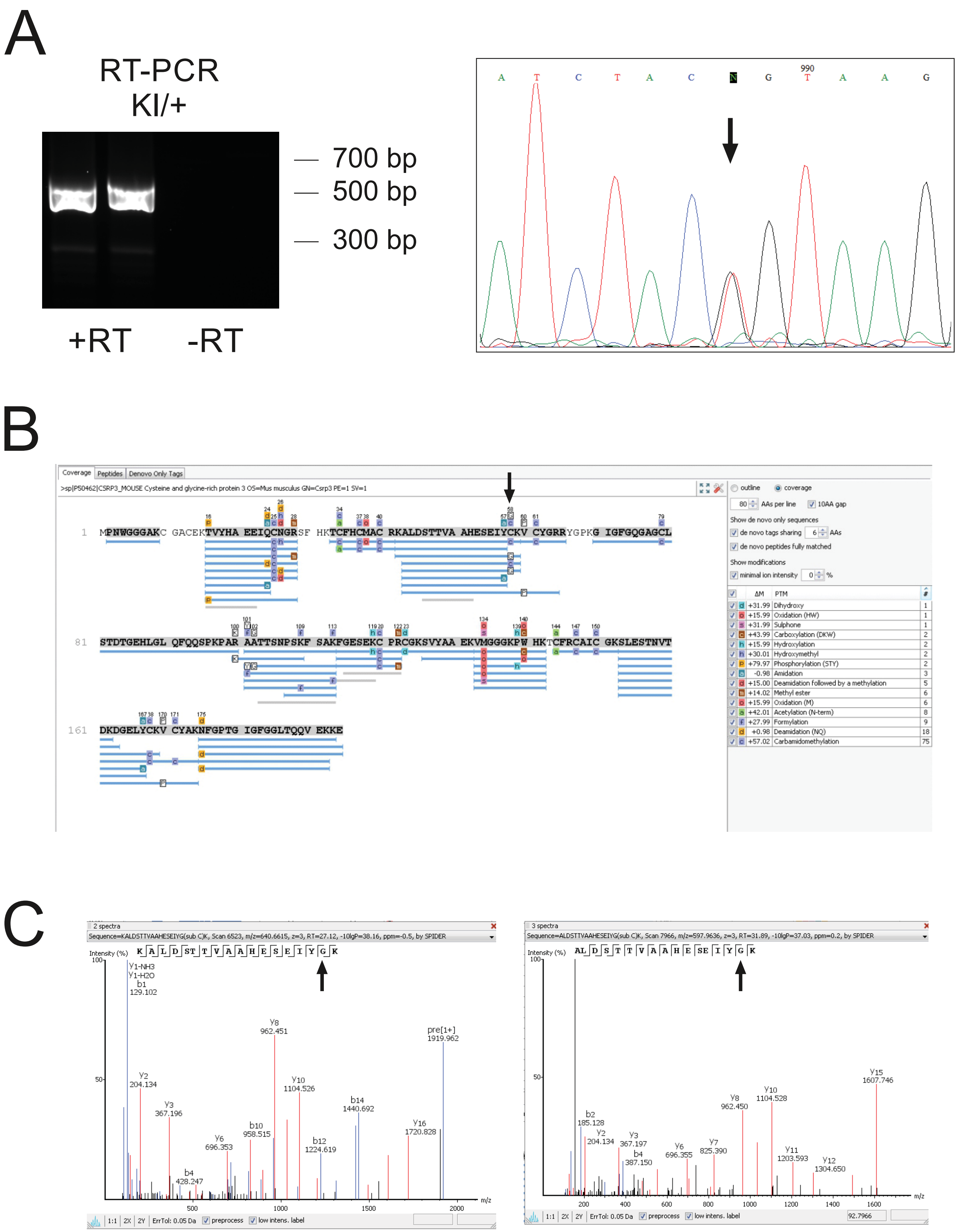
A
RT-PCR
KI/+
700 bp
500 bp
300 bp
+RT
-RT
A T C T A C N G T A A G
990
B
Coverage
Peptides
Denovo Only Tags
>sp|P50462|CSRP3_MOUSE Cysteine and glycine-rich protein 3 OS=Mus musculus GN=Csrp3 PE=1 SV=1
1 MPNWGGGAKC GACEKTVYHA EEIQCNGRSF HKTCFHCMAC RKALDSTTVA AHESEIYCKV CYGRRYGPKG IGFGQGAGCL
81 STDTGEHLGL QFQQSPKPAR AATTSNPSKF SAKFGESEKC PRCGKSVYAA EKVMGGGKPW HKTCFRCAIC GKSLESTNVT
161 DKDGELYCKV CYAKNFGPTG IGFGGLTQQV EKKE
outline
coverage
AAs per line
10AA gap
Show de novo only sequences
de novo tags sharing
AAs
de novo peptides fully matched
Show modifications
minimal ion intensity
ΔM
PTM
+31.99 Dihydroxy
+15.99 Oxidation (HW)
+31.99 Sulphone
+43.99 Carboxylation (DKW)
+15.99 Hydroxylation
+30.01 Hydroxymethyl
+79.97 Phosphorylation (STY)
-0.98 Amidation
+15.00 Deamidation followed by a methylation
+14.02 Methyl ester
+15.99 Oxidation (M)
+42.01 Acetylation (N-term)
+27.99 Formylation
+0.98 Deamidation (NQ)
+57.02 Carbamidomethylation
C
2 spectra
Sequence=KALDSTTVAAHESEIYG(sub C)K, Scan 6523, m/z=640.6615, z=3, RT=27.12, -10lgP=38.16, ppm=-0.5, by SPIDER
Intensity (%)
y1-NH3
y1-H2O
b1
129.102
y2
204.134
y3
367.196
b4
428.247
y6
696.353
b10
958.515
y8
962.451
y10
1104.526
b12
1224.619
b14
1440.692
y16
1720.828
pre[1+]
1919.962
m/z
1:1 2X 2Y ErrTol: 0.05 Da preprocess low intens. label
3 spectra
Sequence=ALDSTTVAAHESEIYG(sub C)K, Scan 7966, m/z=597.9636, z=3, RT=31.89, -10lgP=37.03, ppm=0.2, by SPIDER
b2
185.128
y2
204.134
y3
367.197
b4
387.150
y6
696.355
y7
825.390
y8
962.450
y10
1104.528
y11
1203.593
y12
1304.650
y15
1607.746
92.7966

Figure S2

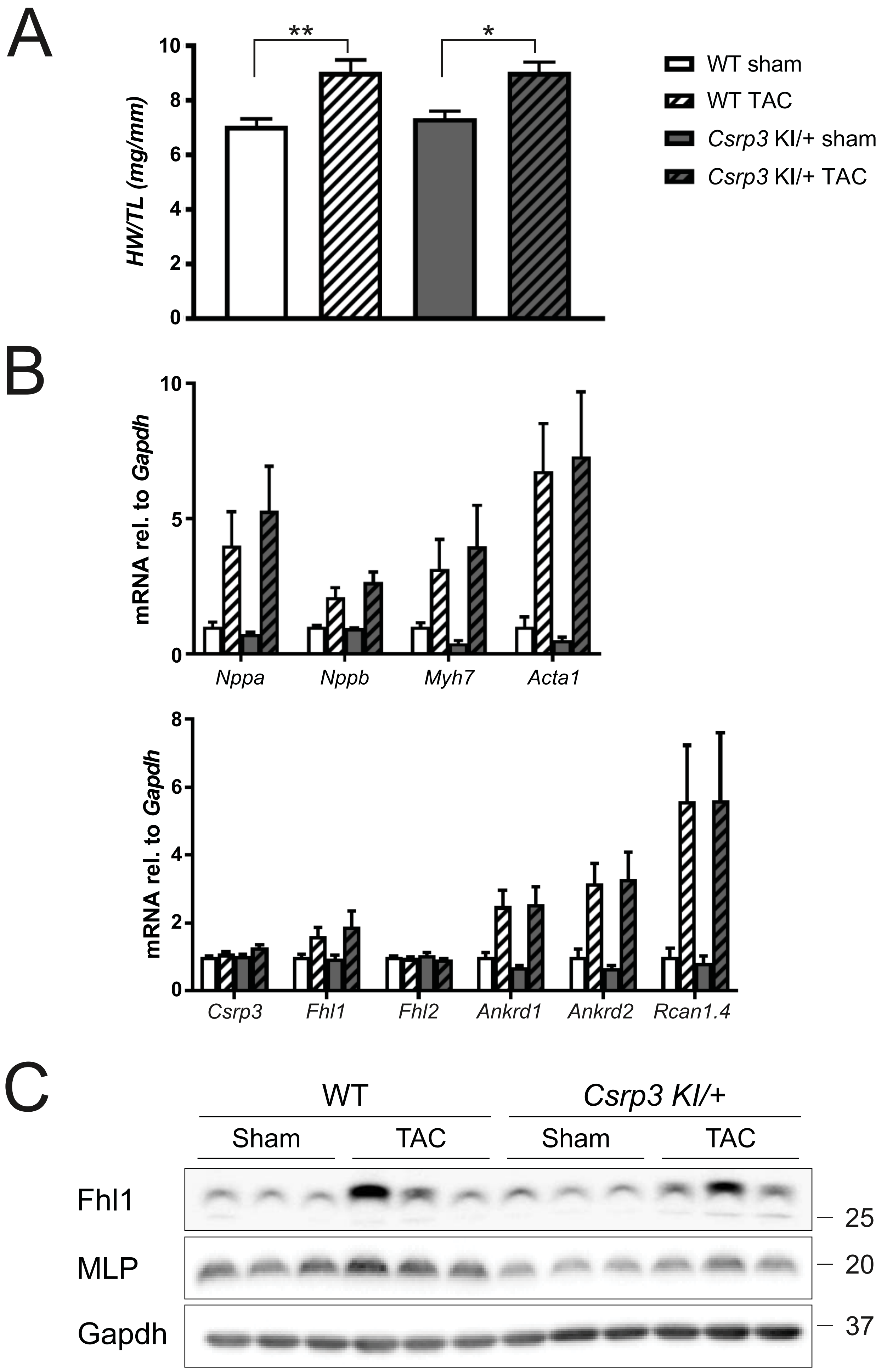
A
**
*
HW/TL (mg/mm)
10
8
6
4
2
0
WT sham
WT TAC
Csrp3 KI/+ sham
Csrp3 KI/+ TAC
B
mRNA rel. to Gapdh
10
5
0
Nppa
Nppb
Myh7
Acta1
mRNA rel. to Gapdh
8
6
4
2
0
Csrp3
Fhl1
Fhl2
Ankrd1
Ankrd2
Rcan1.4
C
WT
Csrp3 KI/+
Sham
TAC
Sham
TAC
Fhl1
MLP
Gapdh
25
20
37

Figure S3

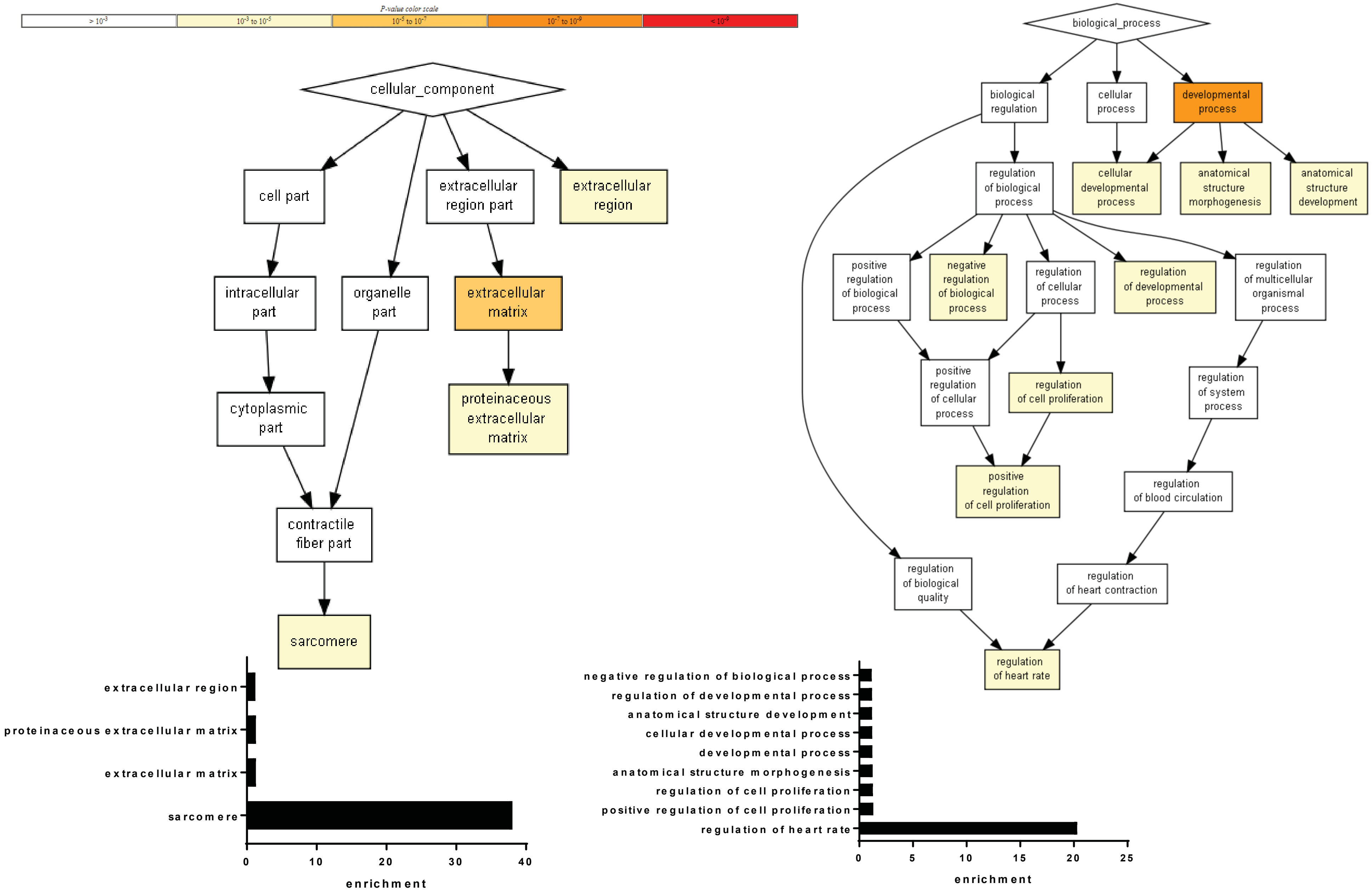

GO Component

GO Process

Figure S4

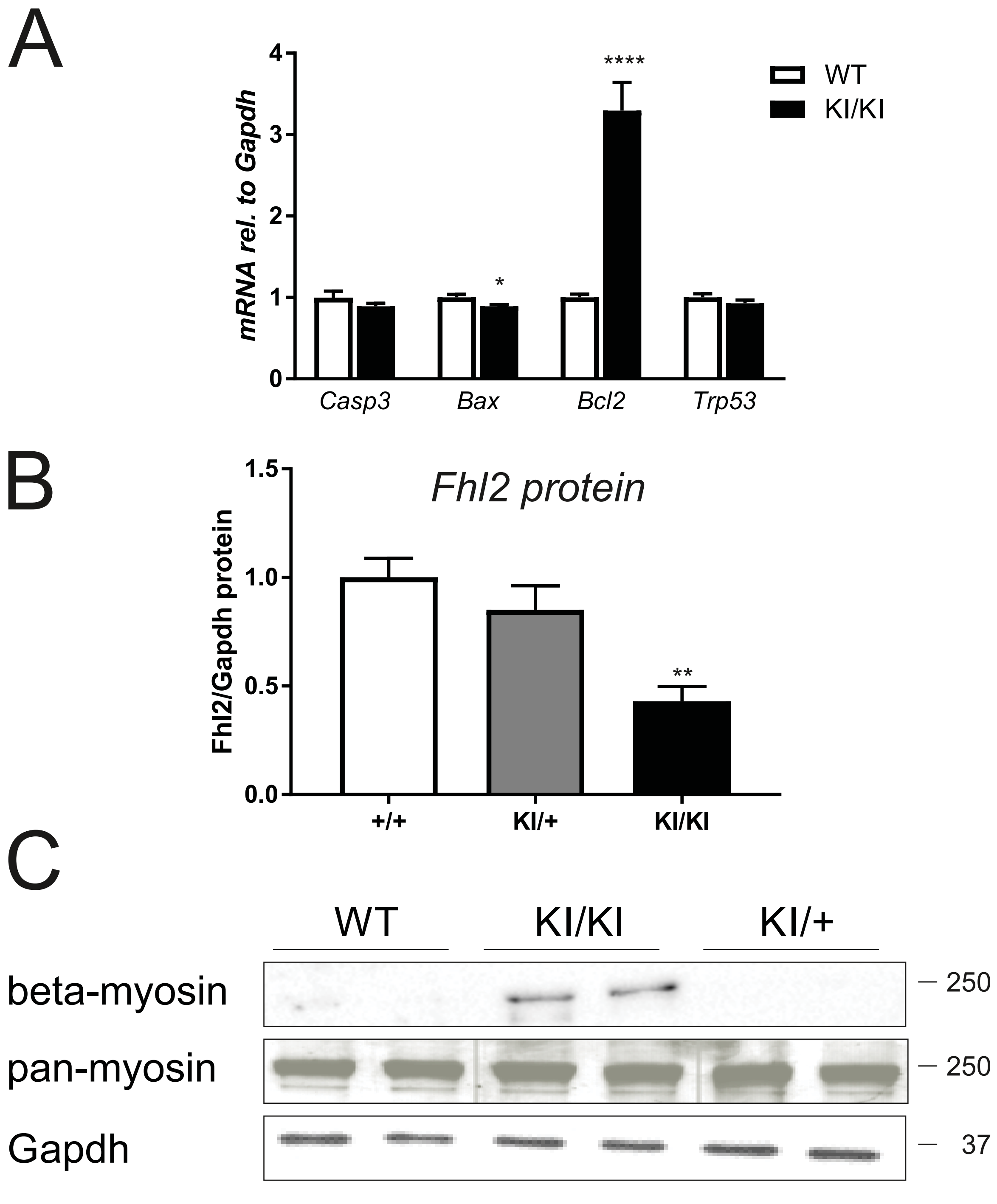
A
4
3
2
1
0
mRNA rel. to Gapdh
****
*
WT
KI/KI
Casp3
Bax
Bcl2
Trp53
B
Fhl2 protein
1.5
1.0
0.5
0.0
Fhl2/Gapdh protein
**
+/+
KI/+
KI/KI
C
WT
KI/KI
KI/+
beta-myosin
pan-myosin
Gapdh
250
250
37

Figure S5

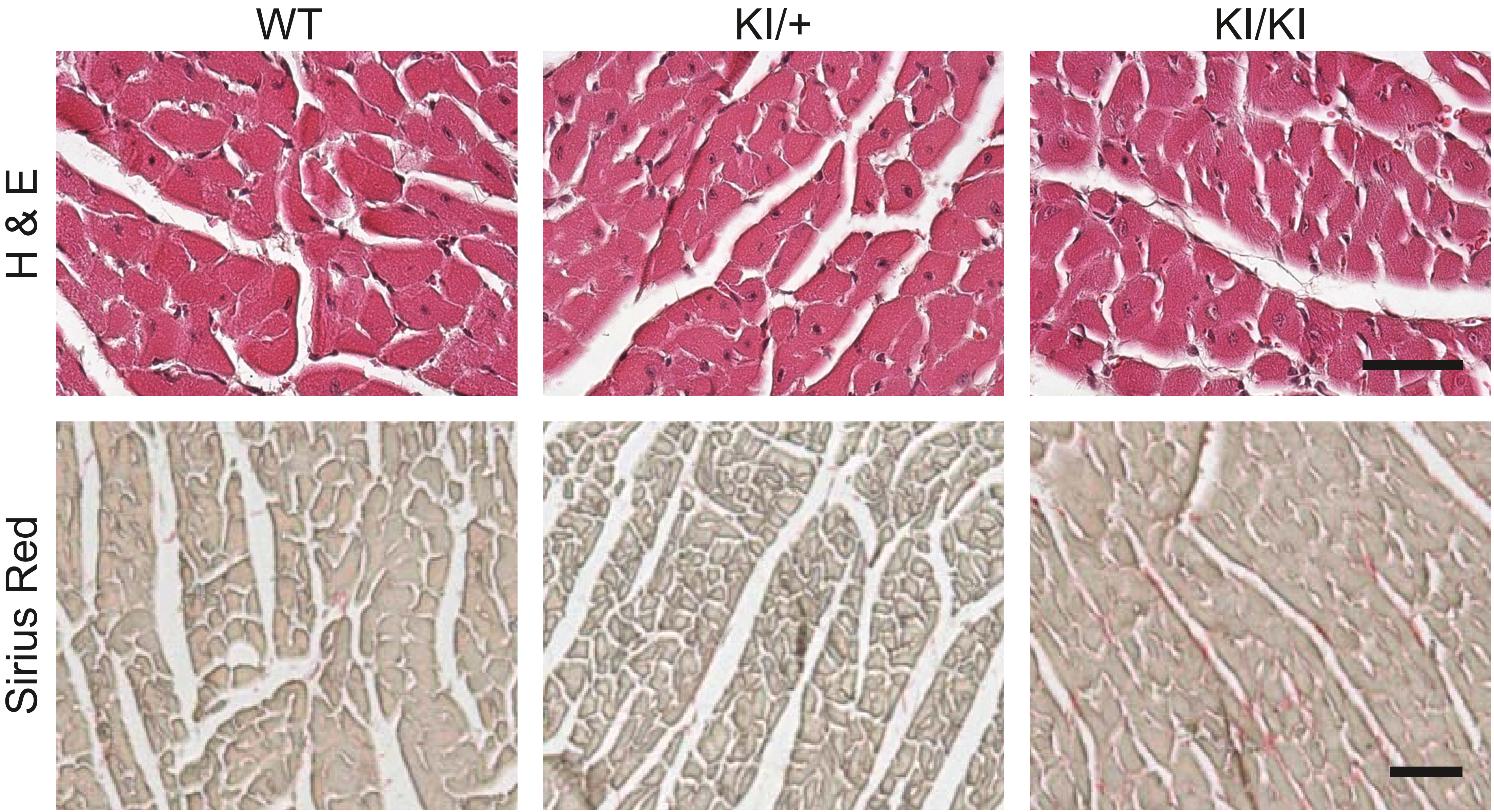

Figure S6

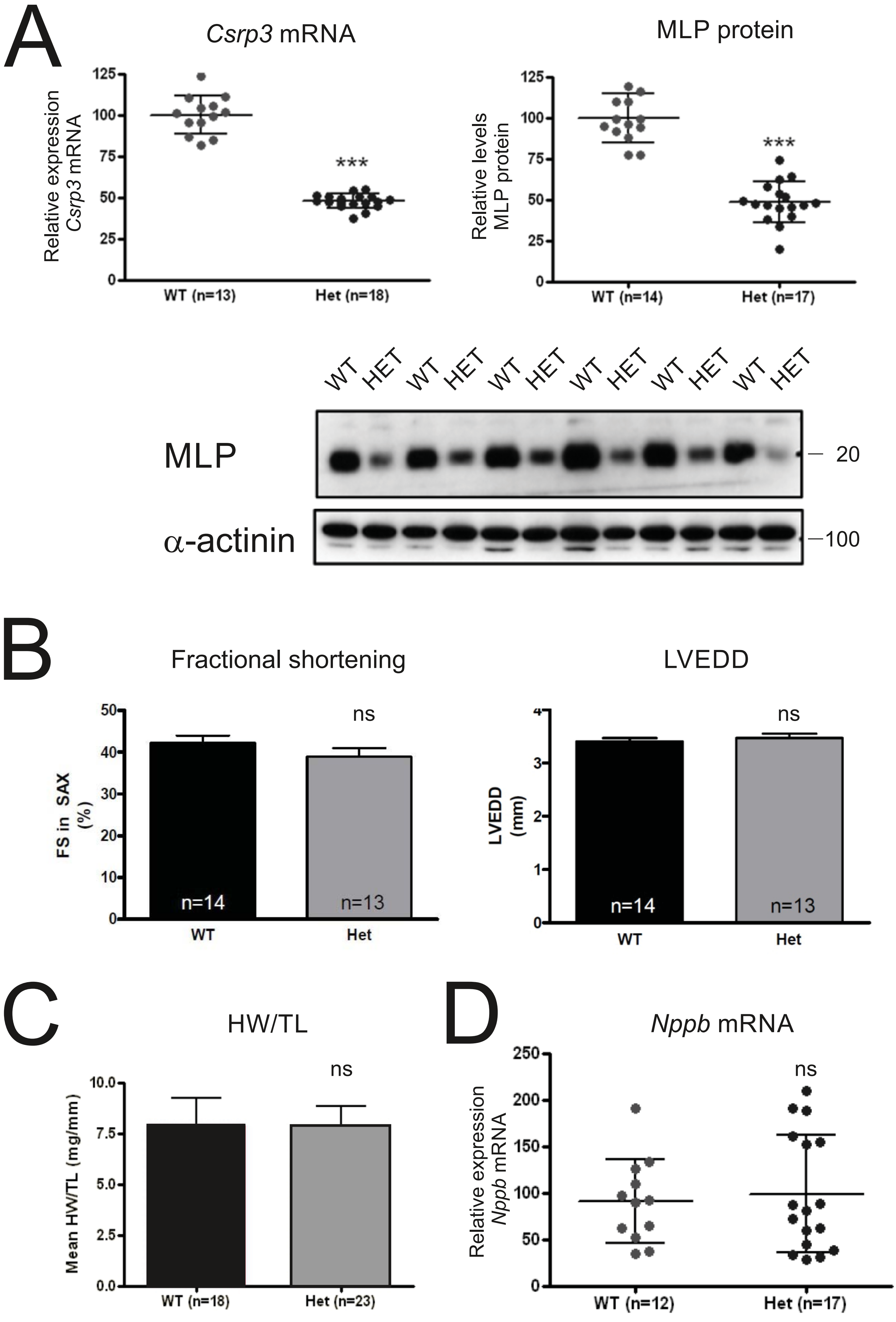
A
Csrp3 mRNA
Relative expression Csrp3 mRNA
125
100
75
50
25
0
***
WT (n=13)
Het (n=18)
MLP protein
Relative levels MLP protein
125
100
75
50
25
0
***
WT (n=14)
Het (n=17)
WT HET WT HET WT HET WT HET WT HET WT HET
MLP
20
α-actinin
100
B
Fractional shortening
FS in SAX (%)
50
40
30
20
10
0
ns
n=14
n=13
WT
Het
LVEDD
LVEDD (mm)
4
3
2
1
0
ns
n=14
n=13
WT
Het
C
HW/TL
Mean HW/TL (mg/mm)
10.0
7.5
5.0
2.5
0.0
ns
WT (n=18)
Het (n=23)
D
Nppb mRNA
Relative expression Nppb mRNA
250
200
150
100
50
0
ns
WT (n=12)
Het (n=17)

Figure S7

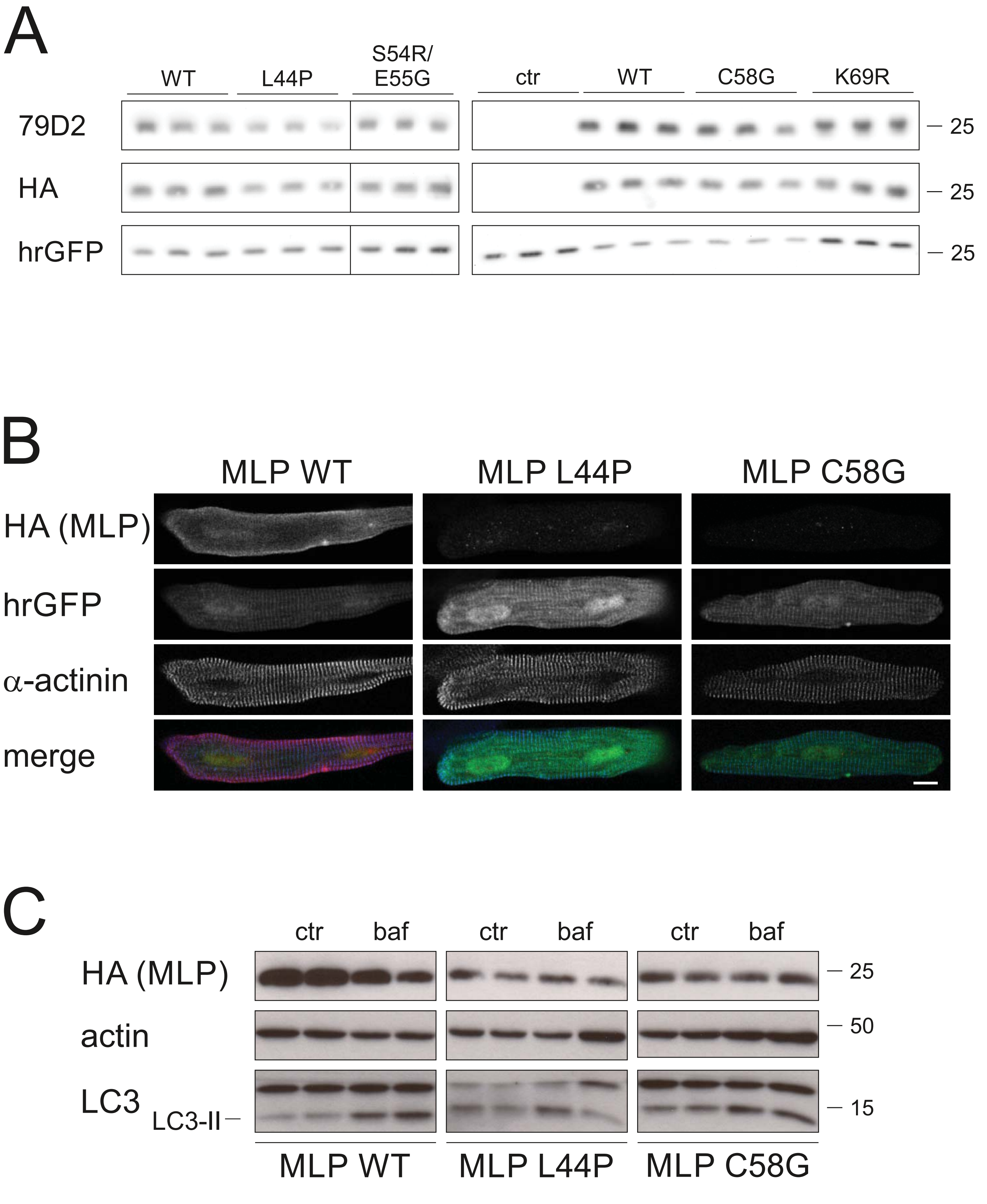
A
WT
L44P
S54R/
E55G
ctr
WT
C58G
K69R
79D2
HA
hrGFP
25
25
25
B
MLP WT
MLP L44P
MLP C58G
HA (MLP)
hrGFP
α-actinin
merge
C
ctr
baf
ctr
baf
ctr
baf
HA (MLP)
actin
LC3
LC3-II
25
50
15
MLP WT
MLP L44P
MLP C58G

Figure S8

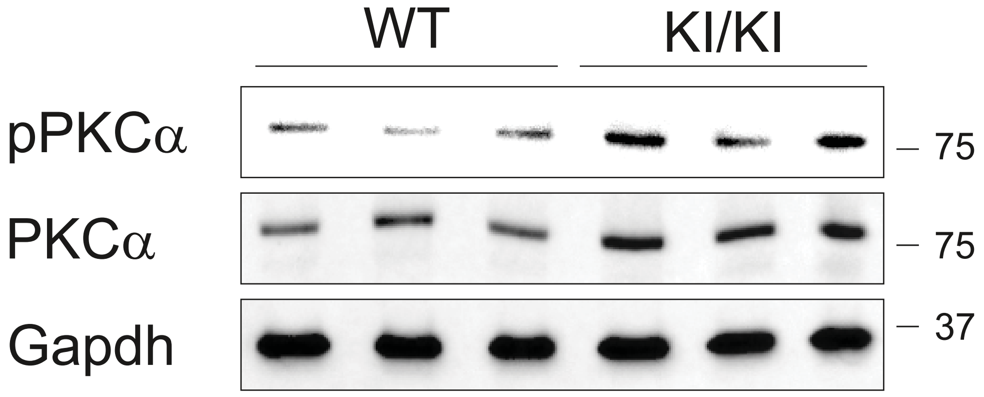

Figure S9

Table S1 Echocardiographic parameters of WT, KI/+ and KI/KI hearts (mean ± SEM) at 2 and 6 months. LVM – calculated LV mass. Please note that the 6 months old mice additionally carry the ubiquitin G76V-GFP reporter transgene.

**2 months:**		**WT**	**KI/+**	**KI/KI**
Body weight	g	24.5 ± 0.9	24.1 ± 0.8	25.3 ± 0.6
n		7	8	7
FS	%	46.0 ± 1.7	50.0 ± 1.6	13.6 ± 1.2****
LVEDD	mm	3.41 ± 0.09	3.27 ± 0.02	4.39 ± 0.10****
LVESD	mm	1.85 ± 0.10	1.64 ± 0.06	3.80 ± 0.12****
LVAWD	mm	1.16 ± 0.03	1.19 ± 0.04	0.98 ± 0.04*
LVPWD	mm	0.89 ± 0.02	0.91 ± 0.03	0.88 ± 0.03
LVM	mg	103.9 ± 4.1	101.4 ± 4.6	135.2 ± 6.4**
HR	bpm	486 ± 2	485 ± 3	455 ± 5****
HW/TL	mg/mm	7.3 ± 0.3	7.0 ± 0.2	9.7 ± 0.4****
**6 months:**		**WT**	**KI/+**	**KI/KI**
Body weight	g	34.6 ± 1.2	34.2 ± 1.0	31.5 ± 1.5
n		8	8	6
FS	%	44.5 ± 1.8	51.3 ± 2.7	30.4 ± 3.4**
LVEDD	mm	3.50 ± 0.13	3.69 ± 0.07	3.55 ± 0.14
LVESD	mm	1.95 ± 0.11	1.81 ± 0.12	2.57 ± 0.23*
LVAWD	mm	1.08 ± 0.04	1.21 ± 0.03*	1.21 ± 0.02*
LVPWD	mm	0.87 ± 0.03	0.86 ± 0.03	0.95 ± 0.03
LVM	mg	101.8 ± 8.2	118.5 ± 4.8	124.9 ± 8.1
HR	bpm	498 ± 8	489 ± 9	482 ± 7
HW/TL	mg/mm	7.3 ± 0.2	7.3 ± 0.3	8.0 ± 0.3

* $p < 0.05$, ** $p < 0.01$, **** $p < 0.0001$ versus WT

Table S2 Haemodynamic parameters of WT, KI/+ and KI/KI hearts (mean ± SEM)

		WT	KI/+	KI/KI
n		9	6	7
baseline				
Heart rate	(bpm)	449 ± 18	455 ± 15	420 ± 11
dP/dT_{max}	(mmHg/s)	8168 ± 617	8415 ± 212	3417 ± 135****
dP/dT_{min}	(mmHg/s)	-6516 ± 486	-6329 ± 286	-1650 ± 134****
Tau	(ms)	8.28 ± 0.45	8.27 ± 0.19	27.63 ± 2.16****
LVESP	(mmHg)	91.23 ± 3.59	99.50 ± 7.18	75.48 ± 1.30**
LVEDP	(mmHg)	4.43 ± 1.11	6.06 ± 1.11	14.30 ± 1.54****
Mean AP	(mmHg)	39.65 ± 3.12	43.90 ± 2.89	48.02 ± 1.35*
Dobutamine (low dose; 4 ng g^{-1} BW min^{-1})				
Heart rate	(bpm)	475 ± 14	480 ± 13	435 ± 11
dP/dT_{max}	(mmHg/s)	9908 ± 490	9024 ± 333	3638 ± 141****
dP/dT_{min}	(mmHg/s)	-6846 ± 382	-6325 ± 326	-1750 ± 142****
Tau	(ms)	7.75 ± 0.46	8.02 ± 0.22	24.87 ± 2.16****
$\Delta dP/dT_{max}$	(mmHg/s)	1740 ± 529	609 ± 517	221 ± 47*
Dobutamine (high dose; 16 ng g^{-1} BW min^{-1})				
Heart rate	(bpm)	499 ± 14	508 ± 14	463 ± 7
dP/dT_{max}	(mmHg/s)	10295 ± 432	9816 ± 256	4115 ± 146****
dP/dT_{min}	(mmHg/s)	-7013 ± 370	-6539 ± 342	-2049 ± 142****
Tau	(ms)	7.32 ± 0.32	7.24 ± 0.29	19.44 ± 1.40****
$\Delta dP/dT_{max}$	(mmHg/s)	2128 ± 400	1402 ± 383	698 ± 111*

* $p < 0.05$, ** $p < 0.01$, **** $p < 0.0001$ versus WT

Table S3 Echocardiographic parameters of WT and KI/+ mice sham and TAC-operated (mean ± SEM)

		**WT sham**	**WT TAC**	**KI/+ sham**	**KI/+ TAC**
n		10	11	10	10
FS	%	46.5 ± 2.2	38.2 ± 2.7	42.7 ± 1.5	46.7 ± 3.1
LVEDD	mm	3.38 ± 0.09	3.62 ± 0.12	3.64 ± 0.11	3.71 ± 0.09
LVESD	mm	1.82 ± 0.12	2.26 ± 0.15	2.09 ± 0.10	1.99 ± 0.15
LVAWD	mm	1.19 ± 0.02	1.27 ± 0.03	1.10 ± 0.05	1.28 ± 0.05*
LVPWD	mm	0.88 ± 0.02	1.02 ± 0.04	0.89 ± 0.03	1.04 ± 0.06
HR	bpm	482 ± 7	484 ± 3	496 ± 4	487 ± 4
HW/TL	mg/mm	7.1 ± 0.3	9.0 ± 0.5**	7.3 ± 0.3	9.0 ± 0.3*

2-Way ANOVA * $p < 0.05$ versus KI/+ sham, ** $p < 0.01$ versus WT sham

	A	B	C	D	E	F	G	H	I	J	K	L	M	N
1	***Table S4: Differentially expressed genes (p<0.001, lfc >1, <-1, >2 RPKM) in KI/KI hearts compared to WT hearts.***													
2										**RPKM**				
3	***Gene symbol***	***log2FoldChange***	***lfc SE***	***p value adjusted***	***KI1***	***KI2***	***KI3***	***KI4***	***WT1***	***WT2***	***WT3***	***KI Avg***	***Wt Avg***	***fold change***
4	*Acta1*	4.823	0.106	0	326074.1	359868.3	263128.8	277757.3	11075.4	11002.5	10439.2	306707.1	10839.1	28.3
5	*Col8a1*	2.522	0.090	4.39E-168	11560.5	12324.7	13483.8	12002.6	2285.3	1891.0	2272.3	12342.9	2149.6	5.7
6	*Thbs4*	4.587	0.168	2.83E-160	9536.1	9655.3	8292.7	5330.3	345.5	341.3	337.2	8203.6	341.3	24.0
7	*Cilp*	3.930	0.148	5.14E-151	15780.8	14039.2	18222.3	10798.8	1084.9	875.0	935.0	14710.3	965.0	15.2
8	*Xirp2*	3.506	0.141	9.34E-134	151961.7	174857.9	123142.3	207961.4	15310.6	13618.6	14499.2	164480.8	14476.2	11.4
9	*Crispld1*	2.665	0.108	8.65E-131	972.1	995.5	1004.7	1015.0	170.5	144.3	156.6	996.8	157.2	6.3
10	*Frzb*	2.205	0.095	8.45E-117	1519.4	1734.9	1645.3	1647.0	384.8	339.0	340.6	1636.6	354.8	4.6
11	*Adcy7*	1.578	0.072	1.53E-102	3514.0	3429.2	3750.8	3697.0	1259.9	1178.2	1176.2	3597.7	1204.8	3.0
12	*Mmp2*	1.552	0.074	1.59E-93	11718.7	10339.3	10950.4	11838.0	3687.7	3765.2	4015.4	11211.6	3822.8	2.9
13	*Frem1*	4.785	0.229	2.26E-93	689.7	760.7	839.7	568.7	15.7	32.4	29.7	714.7	26.0	27.5
14	*Srpx*	2.443	0.117	6.65E-93	1458.9	1407.7	1339.0	1144.8	262.5	250.6	225.2	1337.6	246.1	5.4
15	*Serpinf1*	2.092	0.101	6.65E-93	8224.5	8878.9	7871.6	6648.1	1956.6	1791.4	1817.4	7905.8	1855.1	4.3
16	*Gnao1*	1.869	0.091	5.61E-91	2497.2	2744.3	2764.0	3000.7	695.6	775.4	787.5	2751.5	752.8	3.7
17	*Rbp1*	2.138	0.106	3.69E-87	4510.9	5701.1	4360.8	4353.3	1064.7	1071.9	1089.3	4731.5	1075.3	4.4
18	*Svep1*	2.678	0.133	7.27E-87	3350.9	3192.5	4026.4	2666.2	540.8	457.6	553.2	3309.0	517.2	6.4
19	*Enpp1*	2.117	0.105	8.59E-87	1027.5	1154.5	1103.4	1231.3	252.4	259.6	268.6	1129.2	260.2	4.3
20	*Fibin*	2.121	0.108	3.36E-83	3904.0	3845.1	3915.8	2973.2	862.7	844.8	816.1	3659.5	841.2	4.4
21	*Ankrd1*	2.799	0.144	1.62E-81	251600.6	302079.0	181940.2	263000.9	41006.7	32270.3	34326.1	249655.2	35867.7	7.0
22	*Meox1*	2.133	0.110	8.95E-81	6366.5	8955.6	8352.3	7688.4	1847.8	1736.6	1778.5	7840.7	1787.6	4.4
23	*Serpinb1c*	4.628	0.241	1.74E-79	717.9	741.3	502.8	527.5	32.5	22.4	20.6	622.4	25.2	24.7
24	*Mgp*	1.880	0.100	1.68E-75	39614.8	52469.9	44172.4	45490.0	11423.2	13410.5	12204.0	45436.8	12345.9	3.7
25	*Mrc2*	1.447	0.078	6.52E-74	2766.3	2968.9	3156.1	2907.8	1075.9	1039.5	1130.4	2949.8	1082.0	2.7
26	*Colec12*	1.465	0.080	7.59E-73	3378.2	3613.1	3700.6	3317.2	1320.5	1174.9	1311.0	3502.3	1268.8	2.8
27	*Col9a2*	5.047	0.276	6.37E-72	476.1	576.8	764.8	468.5	15.7	16.8	19.4	571.5	17.3	33.0
28	*Igfbp7*	1.523	0.084	1.14E-70	29299.6	31803.4	29831.9	25917.1	9569.8	10163.3	10759.3	29213.0	10164.1	2.9
29	*Bgn*	1.803	0.101	2.37E-68	38725.5	39208.0	45252.8	33630.8	10323.8	11518.4	11873.7	39204.3	11238.6	3.5
30	*Pam*	1.125	0.064	4.62E-66	49050.6	47116.4	48316.2	44477.1	21100.8	21168.1	22726.7	47240.1	21665.2	2.2
31	*Col4a3*	1.805	0.103	1.69E-65	1327.3	1182.2	1261.6	1096.2	365.7	330.1	349.8	1216.8	348.5	3.5
32	*Nnt*	-1.541	0.089	2.22E-64	11412.3	10478.8	11345.1	9379.7	28608.6	33169.9	31267.4	10654.0	31015.3	0.34
33	*Nppa*	4.857	0.281	2.95E-64	257296.4	253385.7	199512.5	126693.5	7372.0	8490.5	5802.0	209222.0	7221.5	29.0
34	*Pamr1*	2.549	0.147	2.95E-64	1186.5	1382.8	1466.6	1025.5	205.3	190.2	253.8	1265.4	216.4	5.8
35	*Emp1*	1.416	0.082	1.66E-63	15658.3	17233.6	15796.0	15324.0	5494.0	5872.2	6623.8	16003.0	5996.7	2.7
36	*Myh7*	4.214	0.249	9.36E-62	44317.0	41679.6	38208.9	26055.3	1824.2	1669.5	2580.9	37565.2	2024.9	18.6
37	*Lum*	1.768	0.107	3.48E-59	27801.7	30584.3	32769.4	36035.4	8546.7	8811.7	10656.4	31797.7	9338.2	3.4
38	*Kif1a*	3.240	0.196	9.17E-59	377.6	518.5	404.9	463.2	52.7	34.7	52.6	441.1	46.7	9.5
39	*Sulf1*	1.219	0.076	1.48E-55	3429.6	3361.7	3596.0	3313.0	1423.7	1398.7	1591.1	3425.0	1471.2	2.3
40	*Slc1a3*	2.496	0.156	2.95E-55	411.5	489.9	435.6	392.5	79.7	73.9	76.6	432.4	76.7	5.6
41	*Crlf1*	4.058	0.255	1.78E-54	447.1	677.5	433.0	372.4	26.9	23.5	36.6	482.5	29.0	16.6
42	*Comp*	2.074	0.131	1.45E-53	1396.0	1474.3	1250.5	1086.7	351.2	305.5	270.9	1301.9	309.2	4.2
43	*Pla2g4a*	1.808	0.115	1.59E-53	1519.4	1685.0	1351.8	1636.4	451.0	383.8	491.5	1548.2	442.1	3.5

	A	B	C	D	E	F	G	H	I	J	K	L	M	N
3	***Gene symbol***	***log2FoldChange***	***lfc SE***	***p value adjusted***	***KI1***	***KI2***	***KI3***	***KI4***	***WT1***	***WT2***	***WT3***	***KI Avg***	***Wt Avg***	***fold change***
44	*Mfap4*	2.128	0.135	3.95E-53	5641.2	5444.1	6691.7	5881.0	1007.5	1536.3	1515.6	5914.5	1353.1	4.4
45	*Ctsk*	1.616	0.103	4.01E-53	1291.7	1153.5	1153.6	1061.4	406.1	364.8	370.3	1165.0	380.4	3.1
46	*Lrp8*	3.938	0.252	1.20E-52	395.0	329.1	265.4	300.7	19.1	20.1	24.0	322.5	21.1	15.3
47	*Loxl1*	1.126	0.072	1.78E-52	5286.8	5785.2	6040.9	5970.7	2648.8	2579.2	2702.1	5770.9	2643.4	2.2
48	*Prune2*	1.268	0.082	7.64E-52	7241.7	6566.3	6674.7	6723.0	2519.8	2973.0	2977.6	6801.4	2823.5	2.4
49	*Tgfbi*	1.352	0.087	1.21E-51	3562.0	4354.4	3761.0	3726.6	1558.3	1507.2	1459.6	3851.0	1508.4	2.6
50	*Sord*	-1.985	0.128	1.32E-51	2291.9	2347.7	2522.4	3295.0	10142.0	11645.9	9256.2	2614.3	10348.0	0.25
51	*Itih2*	3.107	0.202	3.97E-51	314.6	334.6	390.5	348.2	29.2	36.9	54.9	347.0	40.3	8.6
52	*Acot1*	-2.939	0.194	3.36E-49	62.1	48.1	68.9	54.9	466.7	539.3	342.9	58.5	449.6	0.13
53	*Shisa3*	3.454	0.228	3.49E-49	530.7	711.7	425.4	454.7	58.3	54.8	32.0	530.6	48.4	11.0
54	*Rcn3*	1.231	0.081	4.05E-49	3404.7	3936.6	3945.6	3562.0	1522.4	1594.5	1628.8	3712.2	1581.9	2.3
55	*Dcn*	1.463	0.097	6.74E-49	120147.5	136350.5	117711.4	118903.8	51281.1	39796.3	43085.1	123278.3	44720.8	2.8
56	*Ltbp2*	2.878	0.192	1.50E-48	1797.6	1995.6	1898.8	1099.4	289.5	207.0	196.6	1697.8	231.0	7.3
57	*Aebp1*	1.502	0.100	2.31E-48	1733.8	1693.3	1840.9	1669.1	654.1	520.3	663.0	1734.3	612.4	2.8
58	*Fbp2*	-2.338	0.157	5.79E-48	207.0	132.2	191.4	215.2	1070.3	916.4	840.1	186.5	942.3	0.20
59	*Scd4*	-3.952	0.265	6.90E-48	32.3	23.1	39.1	32.7	698.9	468.8	310.9	31.8	492.9	0.06
60	*Col8a2*	2.655	0.179	1.85E-47	437.2	670.1	635.5	635.2	93.1	107.4	82.3	594.5	94.3	6.3
61	*Lman1l*	3.706	0.251	6.44E-47	337.0	379.0	246.7	273.3	20.2	24.6	26.3	309.0	23.7	13.0
62	*Abat*	1.687	0.115	1.01E-46	1547.5	1508.5	1626.6	1520.4	389.3	560.6	494.9	1550.7	481.6	3.2
63	*Ano10*	-2.037	0.139	3.83E-46	1346.3	1219.2	1194.4	1757.8	5014.9	6611.8	5342.5	1379.4	5656.4	0.24
64	*Acta2*	-1.655	0.115	1.84E-44	2845.8	2815.4	3322.9	3483.9	11212.3	9941.8	8292.6	3117.0	9815.6	0.32
65	*Mxra8*	1.230	0.086	2.87E-44	2083.2	2351.4	2070.6	2110.2	851.5	936.6	967.0	2153.9	918.4	2.3
66	*Nmrk2*	2.586	0.180	3.58E-44	1714.0	1548.2	1383.3	938.0	270.4	219.3	208.0	1395.8	232.6	6.0
67	*Fmod*	2.247	0.157	5.66E-44	1824.9	1680.4	1694.6	1592.1	474.6	343.5	254.9	1698.0	357.7	4.7
68	*Col1a2*	1.615	0.113	1.10E-43	16034.2	14498.6	18136.3	12986.0	4729.9	4798.0	5571.1	15413.8	5033.0	3.1
69	*Abhd18*	-1.868	0.131	1.97E-43	549.0	442.7	626.1	696.4	2019.4	2187.5	2122.6	578.5	2109.8	0.27
70	*D630003M21Rik*	1.346	0.096	3.86E-42	1500.3	1846.8	1548.3	1654.4	642.9	646.7	642.4	1637.4	644.0	2.5
71	*Trabd2b*	-1.015	0.073	6.99E-42	2412.0	2283.0	2235.7	2511.1	4915.1	4870.8	4517.2	2360.4	4767.7	0.50
72	*Fzd1*	1.225	0.089	1.00E-40	991.1	1083.3	1021.7	1013.9	423.0	435.3	460.6	1027.5	439.6	2.3
73	*Fxyd6*	1.188	0.087	2.23E-40	4621.9	5296.3	4656.8	4471.4	1944.3	2076.8	2250.6	4761.6	2090.5	2.3
74	*Casq1*	2.057	0.151	3.81E-40	2134.6	2124.0	2245.9	2180.9	707.9	425.2	432.1	2171.3	521.7	4.2
75	*Pcolce*	1.351	0.099	4.40E-40	6132.2	6628.2	6864.4	5431.6	2216.9	2526.6	2625.5	6264.1	2456.3	2.6
76	*Itgbl1*	2.232	0.164	4.47E-40	5042.5	5148.4	3955.8	2938.4	1007.5	841.4	879.0	4271.3	909.3	4.7
77	*Olfml3*	1.308	0.097	2.38E-39	3241.6	3273.9	2879.7	2788.6	1298.0	1300.2	1092.7	3045.9	1230.3	2.5
78	*Ace*	1.948	0.144	2.67E-39	11400.7	11647.1	10291.9	7417.2	3049.3	2369.9	2504.4	10189.3	2641.2	3.9
79	*Pi16*	1.057	0.078	3.38E-39	8435.6	9767.1	9955.0	8827.9	4523.5	4343.7	4466.9	9246.4	4444.7	2.1
80	*Clec3b*	-1.591	0.118	4.18E-39	1195.6	1129.5	1012.3	1498.2	3686.6	3678.0	3553.7	1208.9	3639.4	0.33
81	*Col4a4*	1.530	0.115	3.95E-38	1603.0	1343.0	1683.6	1317.8	502.6	486.7	556.7	1486.8	515.3	2.9
82	*Fbln2*	1.239	0.093	5.20E-38	10836.8	11771.0	12394.0	9760.6	4460.7	4721.9	5045.3	11190.6	4742.6	2.4
83	*Abca8a*	1.259	0.095	6.20E-38	11521.6	12137.0	11801.9	10719.7	5385.1	4143.4	4944.7	11545.1	4824.4	2.4
84	*Ttll1*	-1.926	0.145	8.96E-38	817.2	778.3	1072.7	1257.7	3695.6	3918.5	3569.7	981.5	3727.9	0.26
85	*Sdc2*	1.028	0.078	1.94E-37	4529.2	5118.8	5157.0	4849.2	2275.2	2396.8	2558.1	4913.5	2410.0	2.0

	A	B	C	D	E	F	G	H	I	J	K	L	M	N
3	***Gene symbol***	***log2FoldChange***	***lfc SE***	***p value adjusted***	***KI1***	***KI2***	***KI3***	***KI4***	***WT1***	***WT2***	***WT3***	***KI Avg***	***Wt Avg***	***fold change***
86	*Arhgef40*	1.167	0.089	2.89E-37	2713.4	3274.8	3027.7	3251.8	1320.5	1340.5	1434.5	3066.9	1365.2	2.2
87	*Ptgfrn*	1.083	0.083	5.02E-37	6369.0	6686.4	6994.6	5847.3	2952.9	2956.2	3258.8	6474.3	3055.9	2.1
88	*Ccl6*	-1.125	0.086	1.45E-36	1023.4	1035.2	1121.2	1213.3	2329.1	2524.3	2326.0	1098.3	2393.2	0.46
89	*Scara5*	-1.366	0.105	1.93E-36	532.4	586.0	686.5	654.2	1657.1	1475.9	1619.7	614.8	1584.2	0.39
90	*Lamc3*	3.650	0.282	3.86E-36	373.4	472.3	240.8	253.2	28.0	23.5	28.6	334.9	26.7	12.5
91	*Pfkfb1*	-3.133	0.244	1.76E-35	31.5	23.1	36.6	17.9	295.1	227.1	203.5	27.3	241.9	0.11
92	*Ccdc68*	1.741	0.136	3.70E-35	448.8	476.9	524.0	434.7	141.4	125.3	156.6	471.1	141.1	3.3
93	*Tril*	1.529	0.120	4.14E-35	1492.1	1627.7	1452.2	1294.6	464.5	459.9	601.2	1466.6	508.5	2.9
94	*Pla2g5*	-2.045	0.161	6.12E-35	274.1	309.6	371.8	476.9	1626.8	1463.6	1333.9	358.1	1474.7	0.24
95	*Tgfb3*	1.036	0.082	8.38E-35	2301.0	2601.0	2415.2	2215.7	1184.7	1167.1	1135.0	2383.2	1162.3	2.1
96	*Atp6v0a4*	2.797	0.221	1.70E-34	191.3	244.9	244.2	207.9	26.9	34.7	34.3	222.1	32.0	6.9
97	*Nkd2*	2.443	0.194	3.70E-34	558.1	843.0	648.2	468.5	131.3	106.3	109.7	629.4	115.8	5.4
98	*Pdgfrl*	1.849	0.147	3.99E-34	898.4	988.1	1072.7	694.2	250.2	249.5	261.8	913.4	253.8	3.6
99	*Stk32b*	2.786	0.222	4.62E-34	192.1	255.1	241.6	223.7	34.8	39.2	25.1	228.1	33.0	6.9
100	*Olfml1*	1.180	0.094	1.33E-33	928.2	870.7	866.0	829.3	370.2	382.7	404.6	873.6	385.8	2.3
101	*Gpr153*	1.299	0.104	1.39E-33	2221.5	2525.2	2702.7	2958.5	1044.5	1108.9	1017.3	2602.0	1056.9	2.5
102	*Itih5*	1.789	0.143	1.49E-33	3368.3	3249.9	4277.4	3575.7	943.5	867.2	1330.5	3617.8	1047.1	3.5
103	*Scml4*	1.914	0.154	3.20E-33	850.4	1075.9	1177.4	1322.0	245.7	312.2	322.3	1106.4	293.4	3.8
104	*Plbd1*	-1.409	0.114	4.02E-33	1444.0	1060.2	1134.9	1400.1	3492.5	3334.4	3205.0	1259.8	3344.0	0.38
105	*Mfap5*	1.563	0.126	4.24E-33	6395.5	6779.8	7178.3	6816.9	1731.1	2524.3	2639.2	6792.6	2298.2	3.0
106	*Crispld2*	1.753	0.142	6.57E-33	11181.3	12997.6	8366.8	11015.1	3854.9	3004.4	2833.6	10890.2	3230.9	3.4
107	*Acaa2*	-1.278	0.104	9.71E-33	12121.1	12169.4	11308.5	13835.3	32927.9	31736.5	25241.3	12358.6	29968.6	0.41
108	*Whrn*	-2.171	0.176	1.20E-32	238.5	248.6	325.0	432.6	1387.8	1555.3	1250.5	311.2	1397.9	0.22
109	*Creb3l2*	1.121	0.092	2.34E-32	1322.3	1456.7	1520.2	1540.4	674.3	630.0	708.7	1459.9	671.0	2.2
110	*Cyfip2*	-1.050	0.087	9.55E-32	3382.4	2861.6	2797.1	2858.2	6529.5	5953.9	6002.0	2974.8	6161.8	0.48
111	*Rgs6*	-1.362	0.112	1.34E-31	299.7	334.6	362.4	335.5	953.6	831.4	781.8	333.1	855.6	0.39
112	*Crat*	-1.061	0.088	1.67E-31	10230.7	10257.0	9130.7	10310.3	22610.9	21492.6	18358.1	9982.2	20820.5	0.48
113	*Prepl*	-1.244	0.103	1.82E-31	1350.5	1282.0	1362.8	1640.7	3003.3	3553.8	3447.4	1409.0	3334.8	0.42
114	*4430402I18Rik*	-1.386	0.115	2.75E-31	268.3	271.7	284.1	326.0	803.3	772.1	674.4	287.5	749.9	0.38
115	*Impa2*	-1.749	0.146	4.37E-31	231.0	206.1	266.3	195.2	779.7	862.7	627.5	224.6	756.6	0.30
116	*Fgf16*	-1.532	0.128	5.67E-31	201.2	192.3	208.4	251.1	679.9	593.0	572.7	213.2	615.2	0.35
117	*Lrrn2*	1.544	0.129	6.02E-31	448.8	475.1	512.1	522.3	154.8	158.9	189.7	489.6	167.8	2.9
118	*Shisa4*	1.956	0.164	1.15E-30	587.1	677.5	433.0	624.6	157.1	152.2	139.4	580.5	149.6	3.9
119	*Cnksr1*	2.059	0.173	1.18E-30	1144.3	1223.8	912.0	1195.4	181.7	290.9	332.6	1118.9	268.4	4.2
120	*Unc5b*	1.024	0.086	1.21E-30	2777.1	2951.3	2915.4	2797.0	1482.0	1236.4	1500.8	2860.2	1406.4	2.0
121	*Rtn4*	1.026	0.086	1.24E-30	7695.4	7696.7	6992.0	8523.0	3753.9	3551.5	4073.7	7726.8	3793.0	2.0
122	*1700040L02Rik*	-1.945	0.164	1.63E-30	168.9	129.4	189.7	234.2	729.2	702.7	649.2	180.6	693.7	0.26
123	*Lrrk1*	1.103	0.093	1.65E-30	2094.8	2260.8	2147.2	2013.1	1073.7	868.3	1032.1	2129.0	991.4	2.1
124	*Prrx2*	3.404	0.287	2.37E-30	134.1	181.2	159.9	178.3	13.5	16.8	16.0	163.4	15.4	10.6
125	*Chodl*	5.698	0.482	3.60E-30	296.4	342.0	240.8	134.0	6.7	4.5	3.4	253.3	4.9	51.9
126	*Fah*	-1.253	0.106	5.35E-30	298.1	293.0	308.0	259.6	733.7	657.9	682.4	289.6	691.3	0.42
127	*Spry1*	1.090	0.093	7.22E-30	2105.6	2350.5	2084.2	1917.1	941.3	1061.9	976.1	2114.4	993.1	2.1

	A	B	C	D	E	F	G	H	I	J	K	L	M	N
3	***Gene symbol***	***log2FoldChange***	***lfc SE***	***p value adjusted***	***KI1***	***KI2***	***KI3***	***KI4***	***WT1***	***WT2***	***WT3***	***KI Avg***	***Wt Avg***	***fold change***
128	*Ech1*	-1.370	0.117	1.09E-29	23590.5	24230.6	19049.2	27203.2	64875.2	64075.0	53395.1	23518.4	60781.8	0.39
129	*P3h2*	1.773	0.152	1.96E-29	659.1	672.0	568.3	474.8	154.8	174.6	192.0	593.5	173.8	3.4
130	*Ltbp3*	1.092	0.094	2.88E-29	2705.1	2923.6	2957.9	2491.1	1265.5	1204.0	1428.8	2769.4	1299.4	2.1
131	*Eci1*	-1.235	0.107	1.37E-28	4985.4	5029.1	4686.6	6193.3	13189.1	12760.4	10921.6	5223.6	12290.4	0.43
132	*Aldh5a1*	-1.135	0.099	1.76E-28	744.4	606.3	707.8	752.3	1439.4	1616.9	1572.8	702.7	1543.0	0.46
133	*Gck*	1.995	0.174	1.91E-28	1892.0	1871.7	1638.5	1660.7	298.4	604.2	426.3	1765.7	443.0	4.0
134	*Bmp4*	1.175	0.102	2.10E-28	948.9	989.9	869.4	870.4	369.1	417.4	435.5	919.7	407.3	2.3
135	*Spp1*	2.852	0.251	5.96E-28	539.0	545.3	314.8	316.5	74.0	42.5	61.7	428.9	59.4	7.2
136	*Hfe2*	-1.167	0.103	6.24E-28	5815.0	5475.6	4865.2	5925.4	14054.1	12261.4	10869.0	5520.3	12394.8	0.45
137	*Abca6*	1.220	0.107	6.37E-28	1474.7	1380.9	1231.8	1203.9	544.1	532.6	627.5	1322.8	568.1	2.3
138	*Etfb*	-1.207	0.106	9.76E-28	12267.6	12915.3	10929.1	14589.7	30126.5	31745.5	25880.3	12675.4	29250.8	0.43
139	*Cadm4*	-1.480	0.131	9.97E-28	202.0	185.8	171.0	223.7	602.5	528.1	502.9	195.6	544.5	0.36
140	*Fth1*	-1.094	0.097	1.05E-27	40769.0	44162.3	37596.4	47994.7	85350.0	100148.6	87574.9	42630.6	91024.5	0.47
141	*Kcnv2*	-1.962	0.174	1.32E-27	91.1	71.2	115.7	104.5	347.8	421.8	347.5	95.6	372.4	0.26
142	*Ackr4*	1.601	0.142	1.52E-27	1444.0	1209.0	1221.6	959.1	447.6	401.7	346.3	1208.4	398.6	3.0
143	*Abca8b*	1.260	0.112	1.70E-27	2538.6	2347.7	2675.5	2209.3	1153.3	857.1	1049.3	2442.8	1019.9	2.4
144	*Igfals*	-2.736	0.243	1.81E-27	32.3	14.8	32.3	25.3	179.5	171.2	176.0	26.2	175.6	0.15
145	*Myom3*	3.832	0.340	1.94E-27	1463.1	1655.4	1282.9	2880.4	133.5	125.3	124.6	1820.4	127.8	14.2
146	*Lrp1*	1.169	0.105	4.77E-27	10099.9	9518.5	10508.9	10051.8	4063.5	3987.9	5348.2	10044.8	4466.5	2.2
147	*Plekhh2*	1.063	0.095	4.82E-27	799.8	792.1	769.0	778.7	378.1	346.9	402.3	784.9	375.8	2.1
148	*Mal*	-1.562	0.140	6.69E-27	317.1	303.2	321.6	214.2	886.3	776.5	903.0	289.0	855.3	0.34
149	*Dnm1*	1.251	0.113	1.08E-26	815.6	1001.9	846.5	964.3	396.0	349.1	397.8	907.1	381.0	2.4
150	*Ret*	-1.333	0.120	1.56E-26	272.4	260.7	270.5	262.7	741.6	568.4	705.2	266.6	671.7	0.40
151	*Col5a2*	1.430	0.129	1.92E-26	8604.6	7490.6	10801.5	7272.7	3024.7	3010.0	3474.8	8542.3	3169.8	2.7
152	*Kcnk2*	1.783	0.162	2.86E-26	537.4	579.5	450.9	628.8	195.2	130.9	152.0	549.2	159.4	3.4
153	*Egflam*	-1.557	0.141	2.88E-26	354.4	288.4	348.8	453.7	1176.9	1036.1	971.6	361.3	1061.5	0.34
154	*Rbp4*	3.555	0.323	3.16E-26	121.7	191.3	213.5	163.5	10.1	12.3	21.7	172.5	14.7	11.7
155	*Emilin1*	1.055	0.096	3.45E-26	3446.1	4017.9	3981.3	4113.8	1728.9	1789.2	2098.6	3889.8	1872.2	2.1
156	*Clec11a*	2.291	0.209	4.42E-26	549.0	487.1	612.5	421.0	65.1	111.9	140.6	517.4	105.9	4.9
157	*Thbs1*	1.309	0.119	4.63E-26	1679.2	2073.2	2160.0	2055.3	935.7	763.1	713.2	1991.9	804.0	2.5
158	*Fstl1*	1.124	0.103	6.85E-26	21880.7	22212.9	27575.8	21288.4	9784.1	10659.0	11539.9	23239.5	10661.0	2.2
159	*Adamts8*	2.416	0.221	8.39E-26	308.0	419.6	305.4	405.2	92.0	42.5	67.4	359.6	67.3	5.3
160	*Cygb*	1.011	0.093	1.75E-25	6307.7	7407.4	5744.0	6280.9	3042.6	3221.4	3317.0	6435.0	3193.7	2.0
161	*Dkk3*	1.564	0.145	2.46E-25	1704.0	1690.6	2254.4	2510.0	729.2	616.5	723.5	2039.8	689.8	3.0
162	*B3gnt9*	1.165	0.108	2.46E-25	577.9	563.8	558.1	625.7	245.7	260.7	270.9	581.4	259.1	2.2
163	*Syndig1*	7.243	0.670	2.67E-25	558.9	727.4	312.2	1132.1	6.7	4.5	2.3	682.7	4.5	151.8
164	*Col26a1*	2.327	0.215	2.78E-25	180.5	175.6	206.7	222.6	48.2	31.3	37.7	196.4	39.1	5.0
165	*Stard10*	-1.236	0.115	2.95E-25	456.2	512.1	435.6	582.4	1126.4	1256.6	1120.2	496.6	1167.7	0.43
166	*Cpxm1*	1.303	0.121	3.63E-25	1008.5	1007.5	883.9	941.1	467.8	352.5	347.5	960.3	389.3	2.5
167	*Tenm3*	1.548	0.144	4.27E-25	634.2	572.1	833.7	637.3	219.9	233.9	233.2	669.3	229.0	2.9
168	*Col1a1*	1.348	0.126	5.18E-25	10038.7	9382.6	11902.3	9147.6	3274.8	3967.8	4679.5	10117.8	3974.0	2.5
169	*Cdh19*	1.174	0.109	6.05E-25	556.4	540.7	530.8	508.6	234.5	223.8	252.6	534.1	237.0	2.3

	A	B	C	D	E	F	G	H	I	J	K	L	M	N
3	***Gene symbol***	***log2FoldChange***	***lfc SE***	***p value adjusted***	***KI1***	***KI2***	***KI3***	***KI4***	***WT1***	***WT2***	***WT3***	***KI Avg***	***Wt Avg***	***fold change***
170	*Padi4*	2.245	0.210	7.45E-25	170.6	187.6	187.2	188.9	38.1	48.1	29.7	183.6	38.7	4.7
171	*Lox*	1.571	0.147	7.93E-25	738.6	795.8	1006.4	686.9	304.0	246.2	265.2	806.9	271.8	3.0
172	*Dbn1*	1.096	0.102	8.03E-25	1256.1	1596.3	1518.5	1404.3	665.3	707.2	653.8	1443.8	675.4	2.1
173	*Dclk3*	3.245	0.306	1.85E-24	196.2	265.3	179.5	127.7	25.8	17.9	17.1	192.2	20.3	9.5
174	*Tpm2*	1.493	0.141	2.00E-24	3614.2	3305.3	2601.5	2521.7	902.0	1202.9	1104.2	3010.7	1069.7	2.8
175	*Uchl1*	1.463	0.138	2.20E-24	694.7	818.0	925.6	687.9	246.8	298.8	305.2	781.5	283.6	2.8
176	*Nox4*	2.022	0.192	3.59E-24	594.5	474.2	577.6	342.9	129.0	110.8	128.0	497.3	122.6	4.1
177	*Lhfpl2*	1.362	0.129	3.62E-24	1380.3	1463.2	1633.4	1517.2	500.4	522.5	727.0	1498.5	583.3	2.6
178	*Hcn2*	-1.586	0.150	4.10E-24	263.3	239.4	296.0	357.7	851.5	1005.9	741.8	289.1	866.4	0.33
179	*Pdgfra*	1.119	0.106	4.32E-24	5766.2	6713.2	4987.7	5349.3	2746.4	2414.7	2718.1	5704.1	2626.4	2.2
180	*Amot*	1.377	0.131	4.55E-24	1035.8	1229.3	877.9	1008.7	448.8	358.1	392.1	1037.9	399.6	2.6
181	*Ssc5d*	1.393	0.133	6.24E-24	1054.0	972.4	1177.4	798.7	363.5	384.9	395.5	1000.6	381.3	2.6
182	*Hspb6*	1.133	0.108	9.97E-24	55976.0	59039.9	48847.9	68787.3	28276.5	26565.9	24701.8	58162.8	26514.7	2.2
183	*Ptger4*	1.490	0.143	1.60E-23	466.2	550.0	445.8	424.1	176.1	183.5	144.0	471.5	167.9	2.8
184	*Nupr1*	1.662	0.160	1.85E-23	515.8	583.2	415.1	450.5	131.3	180.1	154.3	491.2	155.2	3.2
185	*P3h3*	1.147	0.111	2.24E-23	1682.5	1911.5	1903.9	1470.8	797.7	728.4	835.5	1742.2	787.2	2.2
186	*Mmp23*	1.298	0.125	2.89E-23	984.5	1157.2	887.3	843.0	406.1	368.1	406.9	968.0	393.7	2.5
187	*Col22a1*	3.454	0.334	3.34E-23	221.1	289.3	217.8	110.8	22.4	20.1	14.9	209.7	19.1	11.0
188	*Acot2*	-1.419	0.138	3.93E-23	830.5	745.0	694.2	636.2	1897.1	2358.7	1575.1	726.5	1943.7	0.37
189	*Sorl1*	1.291	0.126	9.08E-23	584.6	494.5	538.5	594.0	197.5	249.5	230.9	552.9	226.0	2.4
190	*Etv4*	2.669	0.261	9.47E-23	202.9	309.6	188.9	188.9	33.7	25.7	45.7	222.6	35.0	6.4
191	*Ddah1*	1.441	0.141	9.85E-23	866.9	795.8	954.5	641.5	276.0	316.7	308.6	814.7	300.4	2.7
192	*Nt5dc2*	-1.309	0.128	9.85E-23	274.9	283.8	294.3	359.8	732.6	842.6	673.2	303.2	749.5	0.40
193	*Enpp6*	1.030	0.101	1.01E-22	655.8	682.1	645.7	661.5	317.5	302.1	352.1	661.3	323.9	2.0
194	*Tnfrsf11b*	2.824	0.276	1.05E-22	135.0	164.5	232.2	160.4	20.2	23.5	29.7	173.0	24.5	7.1
195	*Maob*	-1.547	0.152	1.68E-22	599.5	504.7	534.2	844.1	1717.6	1891.0	1826.5	620.6	1811.7	0.34
196	*Ptprn*	1.911	0.188	2.01E-22	752.7	727.4	511.3	574.0	226.6	144.3	140.6	641.3	170.5	3.8
197	*Hadh*	-1.044	0.103	2.81E-22	12236.2	12097.3	10666.2	13631.7	26517.3	26975.4	21707.1	12157.8	25066.6	0.49
198	*Lrrcc1*	1.368	0.135	3.44E-22	1690.8	2009.4	1533.8	1304.1	650.7	585.2	664.1	1634.5	633.3	2.6
199	*9030617O03Rik*	-1.500	0.150	1.26E-21	744.4	693.2	923.0	1140.5	2476.0	2696.6	2250.6	875.3	2474.4	0.35
200	*Itgb5*	1.013	0.102	1.49E-21	9000.3	10408.6	8245.1	7736.9	4411.3	4544.0	4193.7	8847.7	4383.0	2.0
201	*Ndufv3*	-1.054	0.107	2.72E-21	6664.6	7126.4	6561.5	8783.6	14675.6	16260.5	14413.5	7284.0	15116.5	0.48
202	*Mmp14*	1.072	0.108	2.83E-21	2722.5	2545.5	2653.4	3126.2	1290.2	1148.0	1501.9	2761.9	1313.4	2.1
203	*Vwa1*	1.111	0.112	2.94E-21	3182.0	3639.9	2734.2	2770.7	1439.4	1312.5	1529.4	3081.7	1427.1	2.2
204	*Atp8a2*	1.405	0.142	3.25E-21	542.3	503.7	542.8	418.9	204.2	203.6	161.2	501.9	189.7	2.6
205	*Omd*	1.826	0.186	4.62E-21	587.1	676.6	519.8	399.9	182.9	152.2	126.9	545.8	154.0	3.5
206	*Col11a1*	2.870	0.292	5.55E-21	125.9	126.6	131.0	99.2	19.1	19.0	11.4	120.7	16.5	7.3
207	*Fbln5*	1.063	0.108	6.14E-21	3244.1	3749.0	3811.2	2888.8	1561.7	1584.4	1771.7	3423.3	1639.3	2.1
208	*Cst6*	1.530	0.157	9.11E-21	464.5	577.7	558.9	404.1	153.7	191.3	176.0	501.3	173.7	2.9
209	*Naalad2*	1.083	0.111	9.35E-21	1824.9	1748.8	2067.2	1622.7	769.6	832.5	970.4	1815.9	857.5	2.1
210	*Ngef*	2.799	0.287	9.59E-21	120.9	129.4	167.6	125.6	24.7	22.4	11.4	135.9	19.5	7.0
211	*Lrrc55*	1.730	0.178	1.64E-20	377.6	403.0	451.7	472.7	103.2	108.5	173.7	426.2	128.5	3.3

	A	B	C	D	E	F	G	H	I	J	K	L	M	N
3	***Gene symbol***	***log2FoldChange***	***lfc SE***	***p value adjusted***	***KI1***	***KI2***	***KI3***	***KI4***	***WT1***	***WT2***	***WT3***	***KI Avg***	***Wt Avg***	***fold change***
212	*Tmem35a*	-1.649	0.170	1.72E-20	87.8	86.0	120.0	126.6	353.4	303.2	329.2	105.1	328.6	0.32
213	*Tmem200a*	4.368	0.451	1.75E-20	94.4	137.7	110.6	91.8	9.0	3.4	3.4	108.6	5.3	20.7
214	*Ass1*	1.632	0.169	2.25E-20	279.9	339.2	272.2	260.6	79.7	92.9	106.3	288.0	92.9	3.1
215	*Col16a1*	1.405	0.145	2.47E-20	1381.9	1346.7	1660.6	1137.4	446.5	489.0	630.9	1381.7	522.1	2.6
216	*Reln*	1.529	0.158	2.52E-20	630.1	682.1	580.2	440.0	219.9	182.4	204.6	583.1	202.3	2.9
217	*Edn3*	4.007	0.416	3.03E-20	993.6	980.7	427.9	1135.3	41.5	50.4	73.2	884.4	55.0	16.1
218	*Mgst1*	-1.225	0.127	3.60E-20	1085.5	990.9	783.5	973.8	2627.5	2039.8	2052.9	958.4	2240.1	0.43
219	*Tnmd*	3.602	0.374	3.61E-20	223.6	221.8	176.9	87.6	13.5	21.3	9.1	177.5	14.6	12.1
220	*Ccdc136*	1.696	0.177	5.31E-20	230.2	264.4	213.5	273.3	68.4	71.6	86.9	245.3	75.6	3.2
221	*Lama2*	1.023	0.107	5.91E-20	8390.1	7415.7	9904.0	8001.8	3948.0	3896.2	4600.7	8427.9	4148.3	2.0
222	*Pdlim2*	1.044	0.109	6.83E-20	1800.1	2350.5	1976.2	1922.4	879.6	1048.4	1000.1	2012.3	976.1	2.1
223	*Adhfe1*	-1.203	0.126	7.52E-20	1303.3	1180.3	1416.4	1787.3	3295.0	3439.6	3078.2	1421.8	3270.9	0.43
224	*Syt7*	-1.112	0.117	1.03E-19	625.1	684.9	751.2	730.1	1747.9	1487.1	1288.2	697.8	1507.7	0.46
225	*Vgll3*	1.251	0.132	1.59E-19	793.2	1001.9	824.3	708.0	382.6	323.4	342.9	831.9	349.6	2.4
226	*Panx1*	1.388	0.147	1.75E-19	338.7	342.9	302.9	277.5	124.5	120.8	116.6	315.5	120.7	2.6
227	*Cep128*	-1.332	0.141	2.07E-19	321.3	242.2	373.5	331.3	731.5	873.9	789.8	317.0	798.4	0.40
228	*Atp5g1*	-1.062	0.113	2.52E-19	7829.6	7680.0	6975.8	9033.6	13900.4	18601.3	16860.7	7879.8	16454.1	0.48
229	*Negr1*	1.188	0.127	3.12E-19	481.9	598.9	575.9	490.6	223.3	239.5	244.6	536.8	235.8	2.3
230	*Hvcn1*	1.168	0.125	3.28E-19	346.1	368.8	346.2	351.3	159.3	154.4	157.7	353.1	157.2	2.2
231	*Decr1*	-1.022	0.110	6.97E-19	10642.3	9956.6	8759.8	10474.9	23586.9	19760.5	17300.8	9958.4	20216.1	0.49
232	*Apbb1*	-1.044	0.112	8.00E-19	2097.3	2109.3	1992.4	2765.4	4657.0	4817.0	4382.3	2241.1	4618.8	0.49
233	*Itga11*	1.284	0.138	8.46E-19	463.7	446.4	494.3	369.3	179.5	173.4	194.3	443.4	182.4	2.4
234	*Star*	1.540	0.166	9.14E-19	343.6	350.3	266.3	378.8	129.0	110.8	105.2	334.7	115.0	2.9
235	*Sh3rf3*	1.818	0.197	1.46E-18	176.4	204.3	191.4	207.9	70.7	45.9	49.1	195.0	55.2	3.5
236	*Timp1*	1.473	0.160	1.78E-18	497.6	561.1	424.5	615.1	153.7	209.2	203.5	524.6	188.8	2.8
237	*Angpt1*	-1.482	0.161	1.88E-18	519.2	595.3	530.0	466.3	1966.7	1210.7	1247.0	527.7	1474.8	0.36
238	*Gcnt4*	4.083	0.446	2.66E-18	89.4	98.0	96.1	72.8	5.6	3.4	6.9	89.1	5.3	16.9
239	*Retnla*	-2.346	0.256	2.69E-18	39.7	31.4	35.7	47.5	268.1	175.7	142.9	38.6	195.6	0.20
240	*Ptn*	1.042	0.114	2.77E-18	527.4	488.0	534.2	476.9	225.5	258.5	254.9	506.7	246.3	2.1
241	*AI464131*	-1.319	0.145	3.29E-18	122.5	107.2	140.4	113.9	302.9	296.5	308.6	121.0	302.7	0.40
242	*Mme*	-1.407	0.154	3.74E-18	235.2	187.6	261.2	249.0	750.6	567.3	537.2	233.2	618.4	0.38
243	*Slitrk4*	2.165	0.238	3.91E-18	182.2	158.1	167.6	262.7	41.5	43.6	43.4	192.6	42.9	4.5
244	*Ckm*	-1.136	0.125	3.98E-18	28063.4	26949.0	26988.9	35655.5	54918.3	75539.7	63456.0	29414.2	64638.0	0.46
245	*Tbx15*	3.929	0.432	4.55E-18	517.5	505.6	310.5	935.9	42.6	30.2	38.9	567.4	37.2	15.2
246	*Scd1*	1.094	0.121	5.58E-18	1894.5	2242.4	2632.1	2426.7	1120.8	945.5	1163.6	2298.9	1076.6	2.1
247	*Ryr3*	2.012	0.223	7.04E-18	250.1	233.8	171.0	168.8	58.3	44.8	50.3	205.9	51.1	4.0
248	*Arc*	1.845	0.204	7.24E-18	157.3	170.1	200.8	157.2	51.6	45.9	45.7	171.3	47.7	3.6
249	*Mylk4*	-2.445	0.271	7.99E-18	1075.6	755.2	1544.0	1468.7	7286.8	6758.4	5732.3	1210.9	6592.5	0.18
250	*Gpt*	-1.363	0.151	9.37E-18	343.6	336.4	333.5	434.7	1153.3	869.4	768.1	362.1	930.3	0.39
251	*Slc25a22*	-1.223	0.136	1.04E-17	643.4	511.1	689.1	775.5	1488.8	1725.4	1369.3	654.8	1527.8	0.43
252	*Col15a1*	1.270	0.141	1.09E-17	12758.6	12512.3	15213.3	14860.9	4348.5	5711.1	7154.2	13836.3	5737.9	2.4
253	*Ybx2*	-1.213	0.135	1.20E-17	162.3	170.1	176.9	196.2	409.5	454.3	360.1	176.4	407.9	0.43

	A	B	C	D	E	F	G	H	I	J	K	L	M	N
3	***Gene symbol***	***log2FoldChange***	***lfc SE***	***p value adjusted***	***KI1***	***KI2***	***KI3***	***KI4***	***WT1***	***WT2***	***WT3***	***KI Avg***	***Wt Avg***	***fold change***
254	*Srpx2*	1.254	0.141	1.90E-17	1386.1	1464.1	1612.1	1064.6	661.9	527.0	549.8	1381.7	579.6	2.4
255	*Map6*	1.121	0.126	2.19E-17	530.7	573.1	555.5	476.9	281.6	235.0	220.6	534.1	245.7	2.2
256	*Gpat3*	-1.150	0.129	2.52E-17	292.3	311.5	327.5	235.3	696.7	656.8	592.1	291.6	648.5	0.45
257	*Pcdh9*	1.513	0.170	2.57E-17	216.9	231.1	257.8	216.3	68.4	88.4	85.7	230.5	80.9	2.9
258	*Bcl2*	1.159	0.131	2.94E-17	1126.9	1128.6	912.8	824.0	440.9	420.7	480.1	998.1	447.2	2.2
259	*Foxs1*	1.100	0.124	3.43E-17	546.5	647.9	706.9	677.4	285.0	286.4	330.3	644.7	300.6	2.1
260	*Slc36a2*	-1.790	0.202	3.65E-17	143.2	122.0	209.3	249.0	572.2	681.4	618.4	180.9	624.0	0.29
261	*Uck2*	1.449	0.165	5.28E-17	3051.2	3431.0	2147.2	4046.2	1074.8	1244.3	1161.3	3168.9	1160.1	2.7
262	*Ogdhl*	-1.054	0.120	5.31E-17	1487.1	1530.6	1215.7	1546.8	3322.0	3185.6	2490.6	1445.0	2999.4	0.48
263	*Rab15*	1.759	0.200	5.69E-17	300.6	336.4	220.3	371.4	77.4	98.5	96.0	307.2	90.6	3.4
264	*Qsox1*	1.145	0.130	7.00E-17	1978.1	2064.0	1838.4	2721.1	921.1	938.8	1056.2	2150.4	972.0	2.2
265	*Paqr9*	-1.038	0.119	1.17E-16	575.5	512.1	698.4	694.2	1308.1	1309.2	1200.2	620.1	1272.5	0.49
266	*Fam20c*	1.004	0.115	1.31E-16	958.8	1215.5	1001.3	1218.6	574.4	515.8	552.1	1098.5	547.4	2.0
267	*Dio2*	2.716	0.312	1.38E-16	2518.8	2429.1	3771.2	1644.9	469.0	372.6	341.8	2591.0	394.4	6.6
268	*Adamts2*	1.118	0.129	1.78E-16	2305.1	2212.8	2785.2	1882.3	948.0	1050.7	1177.3	2296.4	1058.7	2.2
269	*Ccdc63*	1.637	0.189	1.98E-16	390.0	434.4	669.5	560.2	181.7	143.2	170.3	513.5	165.1	3.1
270	*Adamts20*	3.324	0.385	2.21E-16	169.7	205.2	72.3	125.6	13.5	11.2	18.3	143.2	14.3	10.0
271	*Klhdc8a*	-1.239	0.144	2.86E-16	656.6	447.4	621.9	677.4	1640.2	1315.9	1298.5	600.8	1418.2	0.42
272	*Rab27b*	1.929	0.224	2.96E-16	361.0	279.1	283.3	508.6	108.8	95.1	77.7	358.0	93.9	3.8
273	*Smim3*	-1.276	0.148	2.98E-16	226.9	173.8	188.0	218.4	576.7	428.6	460.6	201.8	488.6	0.41
274	*Pdpn*	1.084	0.127	4.48E-16	627.6	769.9	654.2	596.1	287.2	297.6	352.1	662.0	312.3	2.1
275	*Cpeb1*	1.124	0.132	5.31E-16	632.6	741.3	621.0	529.7	310.8	286.4	272.0	631.1	289.8	2.2
276	*Slc6a17*	-1.776	0.209	5.99E-16	45.5	40.7	45.1	33.8	134.6	151.1	140.6	41.3	142.1	0.29
277	*Sec16b*	1.106	0.130	6.11E-16	408.2	348.5	387.1	407.3	171.7	170.1	198.9	387.8	180.2	2.2
278	*Igsf10*	1.550	0.182	6.24E-16	750.2	578.6	949.4	827.2	214.3	242.8	338.3	776.3	265.1	2.9
279	*Cmss1*	-1.243	0.146	6.84E-16	1300.0	1127.7	1618.9	1870.7	3503.7	3749.6	3249.6	1479.3	3501.0	0.42
280	*Cgref1*	1.831	0.216	8.07E-16	178.8	212.6	154.0	155.1	58.3	48.1	41.1	175.1	49.2	3.6
281	*Abi3bp*	1.106	0.131	1.06E-15	981.2	780.1	1065.1	1114.2	489.2	420.7	462.9	985.1	457.6	2.2
282	*Tmem45a*	1.074	0.127	1.16E-15	592.8	654.4	772.4	747.0	292.8	341.3	350.9	691.7	328.3	2.1
283	*Kcnj14*	3.472	0.413	1.59E-15	192.9	230.2	70.6	140.3	22.4	9.0	11.4	158.5	14.3	11.1
284	*Abhd15*	1.580	0.189	2.00E-15	245.1	228.3	244.2	175.1	84.1	73.9	66.3	223.2	74.8	3.0
285	*Klhl34*	1.690	0.202	2.09E-15	760.1	898.4	597.2	1081.5	182.9	293.2	299.5	834.3	258.5	3.2
286	*Dpy19l3*	1.045	0.125	2.38E-15	340.3	333.7	365.8	334.5	167.2	162.2	170.3	343.6	166.6	2.1
287	*Sox9*	1.458	0.175	2.56E-15	236.8	247.7	308.0	287.0	78.5	105.2	110.9	269.9	98.2	2.7
288	*Scarf2*	1.012	0.122	3.06E-15	852.0	1026.9	809.0	752.3	434.2	439.7	405.8	860.1	426.6	2.0
289	*Loxl3*	1.483	0.179	3.38E-15	915.8	1066.6	847.3	562.4	313.0	282.0	315.5	848.0	303.5	2.8
290	*Tpbg*	2.393	0.289	3.78E-15	88.6	100.7	103.8	93.9	12.3	21.3	21.7	96.8	18.4	5.2
291	*Myot*	2.097	0.253	4.01E-15	7606.0	7803.9	4665.3	8792.0	1327.2	1727.6	2006.0	7216.8	1687.0	4.3
292	*Gria3*	1.513	0.183	4.13E-15	226.0	249.6	200.8	181.5	72.9	71.6	81.2	214.5	75.2	2.9
293	*Adra1a*	-1.385	0.168	4.78E-15	186.3	147.0	228.0	155.1	516.1	492.3	397.8	179.1	468.7	0.38
294	*Plekha4*	1.137	0.139	8.85E-15	427.2	469.5	402.4	520.2	203.1	183.5	233.2	454.8	206.6	2.2
295	*Egr2*	2.304	0.282	8.99E-15	147.4	165.5	316.5	277.5	43.8	45.9	48.0	226.7	45.9	4.9

	A	B	C	D	E	F	G	H	I	J	K	L	M	N
3	***Gene symbol***	***log2FoldChange***	***lfc SE***	***p value adjusted***	***KI1***	***KI2***	***KI3***	***KI4***	***WT1***	***WT2***	***WT3***	***KI Avg***	***Wt Avg***	***fold change***
296	*Dfna5*	2.335	0.286	9.82E-15	107.6	129.4	89.3	145.6	25.8	17.9	26.3	118.0	23.3	5.1
297	*Lbp*	1.239	0.152	1.02E-14	1147.6	1202.5	785.2	871.5	430.8	393.9	449.2	1001.7	424.6	2.4
298	*Serpinb6b*	1.141	0.140	1.33E-14	4730.4	4838.7	4282.5	3115.7	1727.7	2155.1	1887.1	4241.8	1923.3	2.2
299	*Eda*	1.017	0.126	2.15E-14	510.0	479.7	442.4	464.2	198.6	246.2	258.3	474.1	234.4	2.0
300	*Mgam*	2.646	0.330	3.20E-14	109.3	114.6	113.1	220.5	16.8	22.4	27.4	139.4	22.2	6.3
301	*Slc16a7*	-1.476	0.185	3.94E-14	90.3	91.5	139.5	116.1	262.5	345.8	304.0	109.3	304.1	0.36
302	*Col12a1*	2.992	0.374	4.03E-14	929.8	891.0	1397.7	538.1	145.8	88.4	120.0	939.2	118.1	8.0
303	*Olfml2a*	1.073	0.135	4.60E-14	389.2	443.7	431.3	517.0	198.6	209.2	226.3	445.3	211.4	2.1
304	*Ldlr*	1.002	0.126	4.94E-14	703.8	843.9	638.0	752.3	389.3	322.3	388.6	734.5	366.7	2.0
305	*Hectd2*	1.182	0.149	5.80E-14	293.1	310.6	267.1	246.9	123.4	124.2	122.3	279.4	123.3	2.3
306	*Nlrc3*	3.393	0.429	7.65E-14	837.9	777.3	409.2	543.4	49.4	38.0	96.0	642.0	61.1	10.5
307	*Fitm1*	-1.111	0.141	8.30E-14	1753.7	1913.3	1801.8	2734.8	4428.2	4746.5	4110.3	2050.9	4428.3	0.46
308	*Necab1*	1.685	0.213	8.42E-14	284.0	260.7	458.5	272.2	101.0	94.0	102.9	318.9	99.3	3.2
309	*Tg*	-2.655	0.337	8.77E-14	17.4	5.5	19.6	22.2	84.1	111.9	108.6	16.2	101.5	0.16
310	*Cpxm2*	2.611	0.331	9.14E-14	3471.8	3358.0	2080.0	1825.3	543.0	307.7	467.5	2683.8	439.4	6.1
311	*Fzd2*	1.176	0.150	1.18E-13	394.1	435.3	554.7	543.4	224.4	209.2	205.7	481.9	213.1	2.3
312	*Mdk*	1.248	0.159	1.27E-13	277.4	347.5	390.5	383.0	133.5	151.1	156.6	349.6	147.1	2.4
313	*Trpm6*	2.242	0.286	1.34E-13	87.8	82.3	85.1	93.9	16.8	17.9	20.6	87.3	18.4	4.7
314	*Ttll7*	1.056	0.135	1.36E-13	533.2	574.0	450.0	571.9	231.1	242.8	293.8	532.3	255.9	2.1
315	*Bicc1*	1.049	0.134	1.50E-13	1730.5	1876.3	1904.7	1658.6	1101.7	724.0	773.8	1792.5	866.5	2.1
316	*Dct*	1.712	0.219	1.55E-13	551.4	493.6	564.0	909.5	153.7	167.8	254.9	629.6	192.1	3.3
317	*Dbh*	-1.964	0.252	1.78E-13	71.2	106.3	114.0	143.5	592.4	392.7	283.5	108.7	422.9	0.26
318	*Zfp185*	1.723	0.221	1.79E-13	154.0	198.7	176.9	129.8	56.1	43.6	50.3	164.9	50.0	3.3
319	*Fn1*	1.037	0.133	1.89E-13	5803.4	5101.2	7243.0	5430.5	2544.5	2656.4	3417.6	5894.5	2872.8	2.1
320	*1500009L16Rik*	1.387	0.178	1.96E-13	630.1	729.3	413.4	524.4	236.7	205.9	216.0	574.3	219.5	2.6
321	*Gipc2*	1.276	0.165	2.68E-13	256.7	278.2	227.1	280.7	127.9	94.0	100.6	260.7	107.5	2.4
322	*Slc22a3*	-1.900	0.246	3.40E-13	57.1	52.7	84.2	32.7	225.5	222.7	190.9	56.7	213.0	0.27
323	*Cyp1b1*	1.345	0.175	3.58E-13	1425.0	1569.5	1008.1	933.7	446.5	561.7	449.2	1234.1	485.8	2.5
324	*Abca4*	-1.710	0.222	3.71E-13	62.9	90.6	85.9	136.1	255.8	317.8	342.9	93.9	305.5	0.31
325	*Igfbp6*	1.283	0.167	4.73E-13	1850.6	2459.6	1409.6	1447.6	758.4	754.2	697.2	1791.8	736.6	2.4
326	*Rbfox1*	-1.070	0.140	4.84E-13	385.0	356.8	516.4	485.3	983.9	943.3	817.3	435.9	914.8	0.48
327	*Postn*	2.853	0.372	4.89E-13	31480.5	25601.4	47011.2	17669.5	4677.2	3286.3	4672.7	30440.6	4212.1	7.2
328	*Ankrd2*	6.400	0.837	5.32E-13	437.2	500.0	609.1	2521.7	16.8	5.6	13.7	1017.0	12.0	84.4
329	*Il34*	1.186	0.156	6.92E-13	310.5	369.7	306.3	268.0	127.9	152.2	133.7	313.6	137.9	2.3
330	*Tmem150c*	-1.784	0.234	7.14E-13	64.6	61.9	54.4	44.3	262.5	162.2	158.9	56.3	194.6	0.29
331	*Lpin3*	1.190	0.158	1.25E-12	490.2	481.6	547.0	703.7	209.8	273.0	246.9	555.6	243.2	2.3
332	*Kcnip2*	-1.171	0.155	1.25E-12	1082.2	1027.8	1577.2	1628.0	3092.0	3183.4	2701.0	1328.8	2992.1	0.44
333	*Tmem119*	1.124	0.149	1.29E-12	735.3	605.4	895.8	858.8	304.0	388.3	372.6	773.8	355.0	2.2
334	*Sema3d*	1.107	0.147	1.46E-12	460.4	517.6	646.5	474.8	245.7	218.2	267.5	524.8	243.8	2.2
335	*Olfr1033*	1.101	0.147	1.54E-12	332.0	403.0	345.4	362.9	136.9	177.9	189.7	360.8	168.2	2.1
336	*Mthfd2*	1.573	0.210	1.90E-12	828.8	621.1	413.4	482.2	171.7	194.7	225.2	586.4	197.2	3.0
337	*Slc24a2*	2.474	0.331	1.91E-12	168.1	159.0	169.3	416.8	39.3	39.2	44.6	228.3	41.0	5.6

	A	B	C	D	E	F	G	H	I	J	K	L	M	N
3	***Gene symbol***	***log2FoldChange***	***lfc SE***	***p value adjusted***	***KI1***	***KI2***	***KI3***	***KI4***	***WT1***	***WT2***	***WT3***	***KI Avg***	***Wt Avg***	***fold change***
338	*Chil1*	2.308	0.310	2.21E-12	74.5	75.8	94.4	98.1	22.4	16.8	12.6	85.7	17.3	5.0
339	*Piezo2*	1.814	0.244	2.47E-12	231.8	138.6	142.1	158.3	43.8	45.9	53.7	167.7	47.8	3.5
340	*Bambi*	1.157	0.156	2.60E-12	1562.4	1710.0	1577.2	2549.1	854.9	819.1	813.8	1849.7	829.3	2.2
341	*Fam180a*	2.372	0.320	3.09E-12	149.9	141.4	81.7	85.5	25.8	20.1	20.6	114.6	22.2	5.2
342	*Fhl1*	2.982	0.404	3.53E-12	33752.5	35203.9	31313.9	74409.8	5181.0	5706.6	5692.2	43670.0	5526.6	7.9
343	*Emilin2*	-1.248	0.169	3.80E-12	469.5	481.6	729.1	799.8	1494.4	1460.2	1458.5	620.0	1471.0	0.42
344	*Napepld*	1.263	0.172	5.44E-12	475.3	481.6	376.0	626.7	178.4	204.8	228.6	489.9	203.9	2.4
345	*Slc22a4*	1.652	0.226	5.99E-12	360.2	482.5	225.4	403.0	131.3	101.8	117.7	367.8	116.9	3.1
346	*Thrsp*	-1.013	0.139	6.08E-12	574.6	718.2	570.8	821.9	1347.4	1508.3	1204.7	671.4	1353.5	0.50
347	*Twist1*	1.226	0.168	7.49E-12	232.7	330.0	279.9	244.8	113.3	115.3	120.0	271.8	116.2	2.3
348	*C3*	-1.022	0.140	7.53E-12	2014.5	1727.5	1476.0	1563.6	4261.0	2895.8	3176.5	1695.4	3444.4	0.49
349	*Bhlhe41*	1.004	0.138	7.57E-12	491.8	455.7	543.6	549.7	305.2	238.3	219.5	510.2	254.3	2.0
350	*A530016L24Rik*	-1.158	0.160	9.36E-12	696.3	504.7	846.5	591.9	1507.8	1662.7	1248.2	659.8	1472.9	0.45
351	*Slc13a4*	2.975	0.411	1.00E-11	109.3	130.3	53.6	72.8	16.8	10.1	8.0	91.5	11.6	7.9
352	*Kcnj3*	-1.168	0.162	1.39E-11	227.7	192.3	292.6	307.0	628.3	578.5	509.8	254.9	572.2	0.45
353	*Adamtsl2*	1.947	0.271	1.43E-11	4303.1	4384.0	5298.2	3598.9	711.3	1451.3	1258.5	4396.1	1140.3	3.9
354	*Shc4*	2.154	0.300	1.49E-11	176.4	186.7	109.7	291.2	43.8	49.2	35.4	191.0	42.8	4.5
355	*Serpine2*	1.167	0.164	2.35E-11	2872.3	2879.2	2214.4	1699.7	1230.7	976.8	1021.9	2416.4	1076.5	2.2
356	*Sv2b*	2.114	0.298	2.87E-11	82.8	93.4	87.6	127.7	26.9	25.7	14.9	97.9	22.5	4.3
357	*Ptx4*	3.758	0.530	2.96E-11	59.6	71.2	54.4	38.0	5.6	4.5	2.3	55.8	4.1	13.5
358	*Add2*	3.202	0.453	3.43E-11	63.8	74.9	45.9	50.6	3.4	7.8	8.0	58.8	6.4	9.2
359	*Srgap3*	1.101	0.156	3.93E-11	212.8	196.9	216.9	198.4	97.6	92.9	98.3	206.2	96.3	2.1
360	*Aff3*	1.056	0.151	4.92E-11	502.6	398.4	601.5	507.5	256.9	217.1	251.5	502.5	241.8	2.1
361	*Entpd5*	-1.052	0.150	5.26E-11	3799.7	3081.6	4792.1	4875.5	10054.5	8038.5	7636.5	4137.2	8576.5	0.48
362	*Sphk1*	1.424	0.204	6.45E-11	149.9	196.0	147.2	140.3	58.3	60.4	58.3	158.3	59.0	2.7
363	*Agmo*	1.016	0.147	9.57E-11	418.1	458.5	377.7	348.2	223.3	165.6	205.7	400.6	198.2	2.0
364	*Bcas1*	2.207	0.320	1.14E-10	73.7	104.4	124.2	163.5	26.9	30.2	18.3	116.5	25.1	4.6
365	*Megf10*	1.761	0.256	1.18E-10	162.3	129.4	232.2	131.9	56.1	45.9	43.4	164.0	48.5	3.4
366	*8430408G22Rik*	-1.262	0.184	1.20E-10	462.9	446.4	395.6	482.2	707.9	1421.1	1084.7	446.8	1071.2	0.42
367	*Aqp8*	2.119	0.309	1.41E-10	375.1	322.6	195.7	139.3	46.0	54.8	77.7	258.2	59.5	4.3
368	*Dnaaf3*	-1.040	0.153	1.84E-10	171.4	131.3	185.5	162.5	375.8	322.3	306.3	162.6	334.8	0.49
369	*Gdf6*	2.963	0.436	2.04E-10	380.1	587.9	416.8	175.1	51.6	43.6	54.9	390.0	50.0	7.8
370	*Orai2*	1.090	0.161	2.24E-10	220.2	230.2	214.4	215.2	117.8	107.4	84.6	220.0	103.3	2.1
371	*Plxnb1*	-1.228	0.181	2.25E-10	454.6	389.1	706.9	696.4	1397.9	1261.0	1287.0	561.8	1315.3	0.43
372	*Etv5*	1.111	0.164	2.41E-10	725.3	775.5	622.7	459.0	291.7	308.8	297.2	645.6	299.2	2.2
373	*Cnksr2*	1.775	0.262	2.50E-10	129.2	161.8	101.2	139.3	29.2	33.6	53.7	132.9	38.8	3.4
374	*Mfap2*	1.210	0.179	2.86E-10	260.0	236.6	287.5	372.4	124.5	130.9	118.9	289.1	124.8	2.3
375	*Doc2b*	2.395	0.355	2.91E-10	57.1	62.9	55.3	62.2	10.1	12.3	11.4	59.4	11.3	5.3
376	*Epn3*	-2.388	0.354	2.91E-10	194.6	153.4	382.0	338.7	1523.5	1300.2	1371.6	267.2	1398.5	0.19
377	*Rtn4r*	-3.422	0.510	3.53E-10	34.8	26.8	72.3	23.2	614.8	362.5	289.2	39.3	422.2	0.09
378	*Fbln7*	2.278	0.342	4.75E-10	75.3	100.7	74.0	55.9	19.1	11.2	17.1	76.5	15.8	4.8
379	*Nova1*	1.213	0.182	5.13E-10	269.9	280.1	192.3	233.2	112.2	113.0	90.3	243.9	105.2	2.3

	A	B	C	D	E	F	G	H	I	J	K	L	M	N
3	***Gene symbol***	***log2FoldChange***	***lfc SE***	***p value adjusted***	***KI1***	***KI2***	***KI3***	***KI4***	***WT1***	***WT2***	***WT3***	***KI Avg***	***Wt Avg***	***fold change***
380	*Serinc2*	2.192	0.330	5.55E-10	85.3	136.8	87.6	67.5	25.8	20.1	16.0	94.3	20.6	4.6
381	*Gng8*	1.987	0.300	5.81E-10	101.8	92.4	111.4	93.9	11.2	29.1	35.4	99.9	25.2	4.0
382	*Lnx1*	1.027	0.155	6.67E-10	1103.7	938.2	1023.4	1525.7	523.9	538.2	626.4	1147.7	562.8	2.0
383	*Dok5*	2.448	0.370	6.79E-10	65.4	107.2	54.4	110.8	14.6	12.3	19.4	84.5	15.4	5.5
384	*Tbc1d10c*	-1.215	0.184	6.95E-10	140.8	143.3	203.3	134.0	299.5	436.4	347.5	155.3	361.1	0.43
385	*Csl*	-1.005	0.152	7.31E-10	159.8	127.6	136.1	159.3	277.1	333.4	266.3	145.7	292.3	0.50
386	*Ces1d*	-1.734	0.264	9.13E-10	1067.3	1172.9	1323.7	2005.7	4975.6	4690.6	4226.9	1392.4	4631.0	0.30
387	*Gjc2*	1.569	0.239	9.20E-10	101.8	90.6	120.0	102.3	32.5	36.9	35.4	103.7	35.0	3.0
388	*Zfp365*	1.239	0.189	9.20E-10	180.5	200.6	175.2	164.6	67.3	66.0	96.0	180.2	76.4	2.4
389	*Scin*	1.010	0.154	1.04E-09	267.4	236.6	275.6	273.3	127.9	153.3	110.9	263.2	130.7	2.0
390	*Ppl*	-1.264	0.194	1.19E-09	73.7	108.1	91.0	91.8	267.0	205.9	182.9	91.2	218.6	0.42
391	*Runx2*	1.851	0.285	1.35E-09	91.9	96.1	81.7	77.0	18.0	33.6	20.6	86.7	24.0	3.6
392	*AW551984*	2.336	0.362	1.76E-09	85.3	109.1	86.8	221.6	24.7	26.9	22.9	125.7	24.8	5.1
393	*Aspn*	1.509	0.234	1.76E-09	10445.2	10456.6	12747.1	6678.7	3713.5	2988.7	3928.6	10081.9	3543.6	2.8
394	*Tyms*	1.016	0.157	1.76E-09	345.3	307.8	362.4	322.9	125.7	176.8	194.3	334.6	165.6	2.0
395	*Blk*	1.443	0.224	1.93E-09	211.1	162.7	150.6	128.7	52.7	58.2	69.7	163.3	60.2	2.7
396	*Vcan*	1.621	0.252	1.94E-09	3455.2	3220.3	4761.4	4129.6	1025.4	1085.4	1683.7	3891.6	1264.8	3.1
397	*Il1rl2*	1.031	0.160	1.94E-09	207.0	208.0	192.3	207.9	89.8	95.1	114.3	203.8	99.7	2.0
398	*Lgals4*	-1.302	0.202	1.99E-09	246.7	254.2	370.9	231.1	571.0	920.9	549.8	275.7	680.6	0.41
399	*Loxl4*	1.268	0.197	2.07E-09	189.6	159.0	205.9	192.0	78.5	58.2	96.0	186.6	77.6	2.4
400	*Irx2*	-1.076	0.168	2.12E-09	104.3	137.7	142.1	145.6	235.6	308.8	291.5	132.4	278.6	0.48
401	*Poln*	-1.883	0.293	2.15E-09	24.0	34.2	23.8	57.0	114.4	144.3	121.2	34.8	126.6	0.27
402	*Slitrk2*	2.133	0.332	2.21E-09	58.8	73.0	76.6	62.2	12.3	12.3	21.7	67.7	15.5	4.4
403	*Ripor2*	-1.026	0.161	2.56E-09	131.7	160.8	182.9	187.8	290.6	343.5	377.2	165.8	337.1	0.49
404	*Tmem62*	1.017	0.159	2.63E-09	568.8	539.8	441.5	715.3	264.8	309.9	264.0	566.4	279.6	2.0
405	*Scx*	1.240	0.195	3.26E-09	722.8	845.7	472.1	524.4	222.1	288.7	304.0	641.3	271.6	2.4
406	*F830016B08Rik*	-1.033	0.163	3.38E-09	210.3	169.1	149.7	220.5	434.2	378.2	336.0	187.4	382.8	0.49
407	*Zfp385c*	2.077	0.329	4.30E-09	64.6	86.9	54.4	80.2	19.1	17.9	13.7	71.5	16.9	4.2
408	*Gfra1*	-1.364	0.217	4.84E-09	137.4	146.0	158.2	265.9	548.6	434.1	378.3	176.9	453.7	0.39
409	*Pdgfc*	1.180	0.188	5.18E-09	434.7	575.8	332.6	406.2	216.5	156.7	205.7	437.3	193.0	2.3
410	*Ctgf*	2.902	0.462	5.23E-09	11560.5	27101.5	12664.6	9150.7	2170.9	2066.7	1831.1	15119.3	2022.9	7.5
411	*Gli2*	1.354	0.216	5.32E-09	180.5	173.8	204.2	219.5	50.5	76.1	101.7	194.5	76.1	2.6
412	*Robo1*	1.124	0.179	5.32E-09	289.0	239.4	371.8	316.5	132.4	123.1	163.5	304.2	139.6	2.2
413	*Sypl2*	2.643	0.423	6.26E-09	81.1	89.7	90.2	239.5	13.5	15.7	30.9	125.1	20.0	6.3
414	*Opn4*	-1.813	0.291	6.73E-09	22.4	45.3	40.0	55.9	138.0	182.4	107.4	40.9	142.6	0.29
415	*Fam222a*	-1.963	0.315	6.77E-09	13.2	23.1	25.5	31.7	71.8	85.0	114.3	23.4	90.4	0.26
416	*Pdzrn4*	-2.599	0.417	6.77E-09	12.4	2.8	12.8	9.5	46.0	55.9	69.7	9.4	57.2	0.16
417	*Kif19a*	2.093	0.336	6.99E-09	53.8	67.5	57.0	79.1	16.8	14.5	13.7	64.4	15.0	4.3
418	*C1qtnf4*	-1.095	0.176	8.10E-09	91.1	127.6	121.7	150.9	246.8	285.3	251.5	122.8	261.2	0.47
419	*2410004P03Rik*	2.088	0.337	8.52E-09	64.6	66.5	58.7	52.8	11.2	17.9	13.7	60.6	14.3	4.2
420	*Aldh1l1*	2.462	0.399	9.52E-09	342.0	288.4	540.2	793.4	92.0	90.6	84.6	491.0	89.1	5.5
421	*St8sia1*	2.279	0.370	1.08E-08	91.1	140.5	70.6	85.5	7.9	22.4	29.7	96.9	20.0	4.8

	A	B	C	D	E	F	G	H	I	J	K	L	M	N
3	***Gene symbol***	***log2FoldChange***	***lfc SE***	***p value adjusted***	***KI1***	***KI2***	***KI3***	***KI4***	***WT1***	***WT2***	***WT3***	***KI Avg***	***Wt Avg***	***fold change***
422	*Klhl29*	1.842	0.299	1.12E-08	74.5	90.6	78.3	63.3	28.0	19.0	17.1	76.7	21.4	3.6
423	*Slc7a5*	1.076	0.175	1.12E-08	396.6	450.1	336.9	391.4	150.3	164.5	245.7	393.8	186.9	2.1
424	*Aass*	1.534	0.250	1.23E-08	102.7	101.7	141.2	98.1	31.4	38.0	45.7	110.9	38.4	2.9
425	*Tro*	1.319	0.215	1.23E-08	188.0	134.9	239.1	195.2	75.2	80.6	72.0	189.3	75.9	2.5
426	*Pycr1*	1.435	0.234	1.27E-08	115.1	96.1	121.7	135.1	35.9	52.6	41.1	117.0	43.2	2.7
427	*Hyi*	1.172	0.191	1.32E-08	213.6	299.5	188.9	220.5	104.3	94.0	108.6	230.6	102.3	2.3
428	*Dlg2*	1.216	0.199	1.34E-08	172.2	138.6	171.8	188.9	76.3	57.1	83.4	167.9	72.3	2.3
429	*Slc27a6*	2.657	0.434	1.38E-08	49.7	64.7	35.7	68.6	7.9	12.3	5.7	54.7	8.6	6.3
430	*Phkg1*	-1.104	0.181	1.53E-08	168.9	159.0	225.4	277.5	481.3	440.9	413.8	207.7	445.3	0.47
431	*Dhcr24*	1.021	0.168	1.61E-08	307.2	300.4	244.2	377.7	157.1	138.7	157.7	307.4	151.2	2.0
432	*Dync2h1*	1.090	0.180	1.90E-08	247.6	208.0	299.5	249.0	135.8	123.1	94.9	251.0	117.9	2.1
433	*Slc41a2*	1.110	0.183	1.93E-08	163.1	171.0	193.1	157.2	74.0	70.5	93.7	171.1	79.4	2.2
434	*Rhbdl1*	1.388	0.229	2.03E-08	168.1	140.5	150.6	195.2	83.0	42.5	61.7	163.6	62.4	2.6
435	*Ankrd45*	1.243	0.207	2.68E-08	252.5	230.2	176.1	205.7	121.2	73.9	78.9	216.1	91.3	2.4
436	*Mrgprh*	-1.661	0.278	3.24E-08	42.2	39.7	93.6	79.1	234.5	194.7	173.7	63.7	201.0	0.32
437	*Agt*	-1.965	0.330	3.44E-08	13.2	12.9	28.1	24.3	67.3	72.7	89.2	19.6	76.4	0.26
438	*Kif5c*	1.229	0.206	3.52E-08	144.1	155.3	174.4	124.5	72.9	52.6	66.3	149.6	63.9	2.3
439	*Fgf6*	4.619	0.777	3.81E-08	108.5	225.5	68.9	40.1	5.6	4.5	3.4	110.7	4.5	24.6
440	*Cnn1*	2.591	0.436	3.85E-08	474.4	569.4	1256.5	1104.7	109.9	186.9	126.9	851.2	141.2	6.0
441	*Cdkn2b*	1.583	0.267	4.02E-08	96.0	95.2	81.7	68.6	28.0	30.2	27.4	85.4	28.6	3.0
442	*Lypd2*	-2.258	0.384	5.65E-08	8.3	6.5	12.8	20.0	53.9	59.3	54.9	11.9	56.0	0.21
443	*Gabra3*	1.284	0.219	5.87E-08	651.6	442.7	352.2	346.1	151.5	221.6	179.5	448.2	184.2	2.4
444	*Egr3*	2.151	0.369	7.08E-08	53.0	105.4	171.8	117.1	31.4	22.4	21.7	111.8	25.2	4.4
445	*Fndc10*	1.146	0.197	8.18E-08	154.0	137.7	125.9	158.3	74.0	64.9	56.0	144.0	65.0	2.2
446	*Gal3st2c*	-1.657	0.285	8.22E-08	29.0	17.6	39.1	36.9	95.4	110.8	83.4	30.7	96.5	0.32
447	*Angptl3*	-1.367	0.236	8.98E-08	79.5	63.8	88.5	134.0	200.8	288.7	214.9	91.4	234.8	0.39
448	*Rab17*	-1.666	0.288	9.16E-08	26.5	22.2	43.4	54.9	109.9	120.8	116.6	36.7	115.8	0.32
449	*Clcnkb*	1.256	0.219	1.18E-07	114.3	116.5	134.4	127.7	44.9	43.6	66.3	123.2	51.6	2.4
450	*Adam5*	1.969	0.343	1.21E-07	58.8	81.3	90.2	51.7	13.5	23.5	17.1	70.5	18.0	3.9
451	*Hist1h4h*	-1.116	0.195	1.34E-07	112.6	141.4	91.0	160.4	283.8	299.9	234.3	126.4	272.7	0.46
452	*Gabrr2*	-2.046	0.358	1.41E-07	14.9	18.5	10.2	34.8	92.0	73.9	73.2	19.6	79.7	0.25
453	*Hsbp1l1*	-1.318	0.231	1.46E-07	51.3	51.8	70.6	98.1	167.2	177.9	160.0	68.0	168.4	0.40
454	*Adgra1*	3.231	0.567	1.47E-07	33.9	44.4	33.2	29.5	2.2	5.6	3.4	35.3	3.8	9.4
455	*Gsdma*	3.006	0.527	1.47E-07	40.6	37.0	30.6	36.9	2.2	4.5	6.9	36.3	4.5	8.0
456	*Spata33*	-1.147	0.201	1.47E-07	124.2	143.3	177.8	247.9	409.5	374.8	363.5	173.3	382.6	0.45
457	*Dusp4*	1.396	0.245	1.54E-07	154.8	185.8	118.2	115.0	46.0	49.2	68.6	143.5	54.6	2.6
458	*Kcnc1*	3.511	0.619	1.69E-07	71.2	44.4	72.3	16.9	2.2	7.8	3.4	51.2	4.5	11.4
459	*Mfsd7c*	-1.225	0.216	1.70E-07	51.3	46.2	64.7	65.4	111.1	139.9	147.4	56.9	132.8	0.43
460	*Slc26a7*	2.916	0.516	1.90E-07	48.0	32.4	43.4	104.5	4.5	7.8	10.3	57.1	7.5	7.6
461	*Sost*	1.301	0.231	2.07E-07	199.5	291.2	182.1	148.8	90.9	81.7	77.7	205.4	83.4	2.5
462	*Hebp2*	1.030	0.183	2.24E-07	234.3	247.7	186.3	241.6	85.3	129.8	118.9	227.5	111.3	2.0
463	*Lgi1*	-2.198	0.393	2.68E-07	32.3	23.1	17.0	16.9	175.0	61.5	72.0	22.3	102.9	0.22

	A	B	C	D	E	F	G	H	I	J	K	L	M	N
3	***Gene symbol***	***log2FoldChange***	***lfc SE***	***p value adjusted***	***KI1***	***KI2***	***KI3***	***KI4***	***WT1***	***WT2***	***WT3***	***KI Avg***	***Wt Avg***	***fold change***
464	*D430019H16Rik*	1.832	0.328	2.75E-07	122.5	97.1	89.3	64.4	38.1	25.7	14.9	93.3	26.2	3.6
465	*Tmem266*	-1.062	0.192	3.64E-07	73.7	105.4	95.3	125.6	221.0	217.1	185.2	100.0	207.8	0.48
466	*6820408C15Rik*	-1.122	0.203	3.82E-07	85.3	70.2	81.7	98.1	230.0	164.5	152.0	83.8	182.2	0.46
467	*Epyc*	2.463	0.452	5.68E-07	59.6	32.4	74.9	124.5	13.5	10.1	16.0	72.8	13.2	5.5
468	*Cldn1*	2.292	0.422	6.09E-07	53.8	47.1	41.7	33.8	7.9	6.7	12.6	44.1	9.0	4.9
469	*Fhad1*	2.559	0.472	6.48E-07	40.6	35.1	35.7	66.5	11.2	6.7	4.6	44.5	7.5	5.9
470	*Ereg*	2.618	0.484	6.89E-07	31.5	90.6	45.9	53.8	14.6	5.6	6.9	55.4	9.0	6.2
471	*Apod*	1.985	0.368	7.73E-07	962.1	1291.3	840.5	625.7	327.6	148.8	228.6	929.9	235.0	4.0
472	*Syt12*	1.661	0.309	8.57E-07	129.2	156.2	126.8	308.1	46.0	63.8	60.6	180.1	56.8	3.2
473	*Drp2*	1.665	0.311	9.57E-07	82.8	92.4	76.6	86.5	16.8	43.6	19.4	84.6	26.6	3.2
474	*Begain*	1.938	0.363	1.03E-06	65.4	77.6	46.8	76.0	7.9	21.3	22.9	66.5	17.3	3.8
475	*Efnb3*	-1.678	0.314	1.04E-06	465.3	452.0	667.8	983.3	2157.4	1995.1	2007.1	642.1	2053.2	0.31
476	*Irx1*	-1.141	0.214	1.07E-06	92.7	101.7	165.9	86.5	243.5	252.9	244.6	111.7	247.0	0.45
477	*Plcxd2*	2.031	0.381	1.09E-06	86.1	163.6	53.6	71.7	22.4	23.5	22.9	93.8	22.9	4.1
478	*Tmem158*	1.033	0.196	1.52E-06	151.5	191.3	141.2	137.2	83.0	73.9	70.9	155.3	75.9	2.0
479	*Hopx*	-1.443	0.274	1.52E-06	3392.3	3634.4	4792.1	6594.3	12404.9	13338.9	11793.7	4603.3	12512.5	0.37
480	*Myoz1*	2.433	0.463	1.55E-06	41.4	55.5	28.9	45.4	6.7	4.5	12.6	42.8	7.9	5.4
481	*Tubb2b*	1.085	0.206	1.57E-06	113.4	116.5	126.8	125.6	46.0	58.2	66.3	120.6	56.8	2.1
482	*Camk4*	1.549	0.295	1.60E-06	77.0	68.4	107.2	72.8	22.4	24.6	36.6	81.3	27.9	2.9
483	*Shisa6*	-2.503	0.477	1.65E-06	4.1	2.8	11.9	10.6	37.0	49.2	37.7	7.3	41.3	0.18
484	*Klra9*	1.296	0.248	1.76E-06	212.0	136.8	288.4	245.8	107.7	69.4	92.6	220.7	89.9	2.5
485	*Pgpep1l*	2.632	0.503	1.77E-06	33.1	64.7	43.4	26.4	4.5	9.0	6.9	41.9	6.8	6.2
486	*B3galt1*	1.021	0.195	1.82E-06	148.2	150.7	170.1	140.3	94.2	66.0	65.2	152.3	75.1	2.0
487	*Sema5b*	-1.732	0.332	1.89E-06	13.2	15.7	17.9	13.7	52.7	52.6	45.7	15.1	50.3	0.30
488	*Glt8d2*	1.090	0.209	1.94E-06	395.0	385.4	450.0	259.6	230.0	135.4	160.0	372.5	175.1	2.1
489	*Helt*	-1.893	0.363	1.94E-06	9.9	12.9	16.2	13.7	55.0	36.9	54.9	13.2	48.9	0.27
490	*Ccdc83*	1.415	0.272	1.98E-06	63.8	70.2	80.0	74.9	26.9	30.2	24.0	72.2	27.0	2.7
491	*Gnmt*	-1.648	0.317	2.17E-06	15.7	20.3	31.5	36.9	65.1	90.6	88.0	26.1	81.2	0.32
492	*Ccdc74a*	2.272	0.439	2.26E-06	38.1	49.9	34.9	36.9	5.6	12.3	6.9	40.0	8.3	4.8
493	*Tgfb2*	1.801	0.348	2.34E-06	1319.0	1709.0	638.0	1120.5	356.8	307.7	365.8	1196.6	343.4	3.5
494	*Zfp853*	2.311	0.448	2.57E-06	38.9	42.5	27.2	48.5	7.9	9.0	6.9	39.3	7.9	5.0
495	*Drd1*	2.388	0.464	2.76E-06	75.3	73.9	25.5	45.4	9.0	13.4	9.1	55.0	10.5	5.2
496	*Tgfbr3l*	-1.607	0.312	2.76E-06	18.2	13.9	19.6	24.3	56.1	54.8	61.7	19.0	57.5	0.33
497	*Acsm5*	-1.850	0.360	2.84E-06	19.0	18.5	11.9	20.0	89.8	44.8	52.6	17.4	62.4	0.28
498	*Scg3*	2.184	0.429	3.65E-06	29.8	42.5	45.9	39.0	10.1	6.7	9.1	39.3	8.7	4.5
499	*Urah*	-2.752	0.543	3.94E-06	5.0	5.5	3.4	3.2	40.4	23.5	22.9	4.3	28.9	0.15
500	*Msx1*	1.101	0.217	4.06E-06	117.6	166.4	119.1	116.1	63.9	61.5	56.0	129.8	60.5	2.1
501	*Krt18*	1.832	0.362	4.16E-06	67.9	64.7	45.9	111.8	16.8	24.6	19.4	72.6	20.3	3.6
502	*Fras1*	2.952	0.585	4.46E-06	20.7	29.6	45.1	32.7	6.7	2.2	3.4	32.0	4.1	7.7
503	*Armcx6*	1.080	0.215	4.85E-06	113.4	123.9	116.5	153.0	48.2	64.9	66.3	126.7	59.8	2.1
504	*Gal3st3*	-1.141	0.227	4.95E-06	207.0	134.9	299.5	306.0	590.1	525.9	449.2	236.8	521.7	0.45
505	*Edar*	1.788	0.356	4.96E-06	72.0	56.4	51.9	47.5	11.2	24.6	13.7	56.9	16.5	3.4

	A	B	C	D	E	F	G	H	I	J	K	L	M	N
3	***Gene symbol***	***log2FoldChange***	***lfc SE***	***p value adjusted***	***KI1***	***KI2***	***KI3***	***KI4***	***WT1***	***WT2***	***WT3***	***KI Avg***	***Wt Avg***	***fold change***
506	*Aldh1a1*	1.516	0.303	5.25E-06	4384.3	4906.2	3547.5	2496.3	1763.6	1164.8	1091.6	3833.6	1340.0	2.9
507	*Cox6b2*	-1.633	0.326	5.36E-06	32.3	13.9	16.2	26.4	63.9	71.6	70.9	22.2	68.8	0.32
508	*Cdh4*	1.443	0.289	5.66E-06	173.1	174.7	145.5	82.3	63.9	35.8	59.4	143.9	53.1	2.7
509	*Rorb*	1.515	0.303	5.74E-06	58.0	84.1	71.5	57.0	29.2	21.3	20.6	67.6	23.7	2.9
510	*Ism1*	1.110	0.223	6.08E-06	101.0	134.9	96.1	109.7	51.6	52.6	49.1	110.5	51.1	2.2
511	*Mybphl*	3.251	0.654	6.32E-06	18.2	21.3	74.9	71.7	4.5	7.8	2.3	46.5	4.9	9.6
512	*Garem2*	2.470	0.497	6.34E-06	29.8	31.4	44.2	27.4	7.9	5.6	4.6	33.2	6.0	5.5
513	*Efhc1*	1.342	0.271	7.06E-06	87.8	73.9	100.4	72.8	38.1	23.5	37.7	83.7	33.1	2.5
514	*Cda*	1.249	0.254	7.98E-06	135.8	172.8	104.6	134.0	74.0	38.0	60.6	136.8	57.6	2.4
515	*Ankrd55*	1.825	0.371	8.03E-06	36.4	49.0	57.8	48.5	13.5	16.8	10.3	48.0	13.5	3.5
516	*Sox8*	1.535	0.312	8.03E-06	53.0	68.4	58.7	50.6	18.0	22.4	19.4	57.7	19.9	2.9
517	*Neb*	1.274	0.259	8.12E-06	241.8	240.3	269.7	433.6	135.8	73.9	157.7	296.4	122.4	2.4
518	*Fstl3*	1.326	0.270	8.14E-06	123.4	149.7	144.6	221.6	38.1	77.2	75.4	159.8	63.6	2.5
519	*Fam171b*	1.018	0.207	8.32E-06	139.1	119.2	171.0	151.9	60.6	85.0	69.7	145.3	71.8	2.0
520	*Eya2*	1.499	0.305	8.50E-06	269.9	302.2	178.6	113.9	98.7	78.3	52.6	216.2	76.5	2.8
521	*Kank4*	1.025	0.209	8.58E-06	122.5	196.9	161.6	146.7	70.7	77.2	83.4	156.9	77.1	2.0
522	*0610040J01Rik*	-1.555	0.317	8.85E-06	42.2	51.8	36.6	17.9	145.8	87.3	96.0	37.1	109.7	0.34
523	*Ccnjl*	-1.200	0.246	1.02E-05	38.9	37.0	60.4	45.4	121.2	101.8	90.3	45.4	104.4	0.43
524	*Tnni2*	-1.401	0.288	1.03E-05	26.5	31.4	23.8	46.4	99.8	71.6	80.0	32.0	83.8	0.38
525	*Plekhh1*	-1.771	0.364	1.03E-05	27.3	24.0	66.4	29.5	168.3	133.2	76.6	36.8	126.0	0.29
526	*Atcay*	1.323	0.272	1.06E-05	91.1	86.0	103.8	157.2	50.5	38.0	42.3	109.5	43.6	2.5
527	*Cd80*	1.104	0.227	1.09E-05	91.9	98.9	104.6	102.3	57.2	38.0	43.4	99.4	46.2	2.2
528	*Col4a6*	1.280	0.267	1.40E-05	98.5	129.4	208.4	188.9	69.6	57.1	66.3	156.3	64.3	2.4
529	*Cish*	-1.453	0.303	1.44E-05	1088.8	756.1	1363.7	1411.7	2536.6	4239.7	2712.4	1155.1	3162.9	0.37
530	*Angptl7*	1.832	0.382	1.47E-05	709.6	761.6	598.1	567.6	289.5	167.8	98.3	659.2	185.2	3.6
531	*Rad51b*	1.369	0.287	1.64E-05	85.3	53.6	80.0	76.0	33.7	25.7	26.3	73.7	28.6	2.6
532	*Otoa*	3.346	0.702	1.66E-05	30.6	41.6	40.0	9.5	3.4	3.4	2.3	30.4	3.0	10.1
533	*Prss12*	1.891	0.398	1.73E-05	33.9	43.4	41.7	60.1	9.0	15.7	11.4	44.8	12.0	3.7
534	*Id4*	1.524	0.322	1.88E-05	73.7	116.5	56.1	83.4	25.8	22.4	37.7	82.4	28.6	2.9
535	*Dnaic1*	1.251	0.264	1.95E-05	164.8	192.3	132.7	189.9	109.9	57.1	46.9	169.9	71.3	2.4
536	*Nalcn*	1.156	0.245	2.00E-05	143.2	159.0	146.3	96.0	80.8	53.7	49.1	136.1	61.2	2.2
537	*Kcnc2*	-1.350	0.287	2.23E-05	19.9	25.9	34.9	24.3	62.8	69.4	68.6	26.2	66.9	0.39
538	*Cthrc1*	1.471	0.314	2.29E-05	59.6	63.8	95.3	55.9	29.2	21.3	24.0	68.6	24.8	2.8
539	*Vps37d*	1.395	0.298	2.37E-05	54.6	66.5	69.8	53.8	24.7	25.7	19.4	61.2	23.3	2.6
540	*1500015O10Rik*	1.170	0.250	2.38E-05	301.4	197.8	376.0	374.6	170.5	158.9	86.9	312.4	138.8	2.3
541	*Grin2c*	1.781	0.380	2.39E-05	68.7	58.2	94.4	73.9	7.9	20.1	36.6	73.8	21.5	3.4
542	*F2rl1*	1.040	0.222	2.46E-05	151.5	248.6	168.4	142.4	87.5	79.4	92.6	177.8	86.5	2.1
543	*Unc5c*	1.781	0.381	2.49E-05	47.2	52.7	100.4	100.2	29.2	13.4	22.9	75.1	21.8	3.4
544	*Gas2l3*	1.137	0.244	2.56E-05	210.3	261.6	179.5	211.0	62.8	86.2	145.2	215.6	98.0	2.2
545	*Sucnr1*	1.604	0.346	2.87E-05	86.9	108.1	49.3	57.0	30.3	22.4	21.7	75.3	24.8	3.0
546	*Tuba4a*	-1.139	0.245	2.88E-05	11315.4	9068.3	9505.9	16253.6	26439.9	24228.4	25556.8	11535.8	25408.4	0.45
547	*Arhgdig*	1.893	0.408	2.97E-05	64.6	69.3	28.9	61.2	11.2	12.3	21.7	56.0	15.1	3.7

	A	B	C	D	E	F	G	H	I	J	K	L	M	N
3	***Gene symbol***	***log2FoldChange***	***lfc SE***	***p value adjusted***	***KI1***	***KI2***	***KI3***	***KI4***	***WT1***	***WT2***	***WT3***	***KI Avg***	***Wt Avg***	***fold change***
548	*Capn6*	1.238	0.271	3.96E-05	75.3	61.9	86.8	91.8	40.4	32.4	27.4	79.0	33.4	2.4
549	*Myo5c*	-1.040	0.228	4.10E-05	73.7	59.2	96.1	120.3	166.0	200.3	170.3	87.3	178.9	0.49
550	*Slc12a5*	-1.255	0.277	4.68E-05	30.6	34.2	24.7	26.4	77.4	55.9	74.3	29.0	69.2	0.42
551	*Arnt2*	1.522	0.336	4.72E-05	86.9	86.9	98.7	207.9	40.4	51.5	33.1	120.1	41.7	2.9
552	*Penk*	-1.453	0.322	4.96E-05	43.1	73.9	35.7	40.1	193.0	82.8	120.0	48.2	131.9	0.37
553	*Epb41l4b*	-1.138	0.253	5.60E-05	32.3	31.4	40.8	34.8	76.3	67.1	86.9	34.8	76.8	0.45
554	*Cpn2*	-1.531	0.341	5.69E-05	42.2	17.6	48.5	67.5	163.8	116.4	99.4	44.0	126.5	0.35
555	*Klhl33*	-1.190	0.265	5.70E-05	51.3	44.4	77.4	93.9	182.9	152.2	120.0	66.8	151.7	0.44
556	*Zscan18*	1.157	0.258	5.83E-05	146.6	102.6	77.4	129.8	52.7	51.5	49.1	114.1	51.1	2.2
557	*Map3k7cl*	2.630	0.588	5.95E-05	481.9	992.7	185.5	871.5	101.0	109.7	96.0	632.9	102.2	6.2
558	*Rsph1*	1.510	0.339	6.48E-05	47.2	47.1	53.6	40.1	19.1	14.5	16.0	47.0	16.5	2.8
559	*Jsrp1*	1.167	0.262	6.59E-05	77.8	75.8	63.8	69.6	26.9	32.4	36.6	71.8	32.0	2.2
560	*Map7d3*	1.346	0.303	6.63E-05	75.3	61.0	59.5	67.5	26.9	34.7	16.0	65.9	25.9	2.5
561	*Bdh2*	1.291	0.292	7.68E-05	82.0	64.7	95.3	62.2	40.4	30.2	22.9	76.1	31.2	2.4
562	*Fbxl21*	1.990	0.451	7.71E-05	29.8	39.7	26.4	35.9	5.6	10.1	9.1	32.9	8.3	4.0
563	*Sbk2*	-1.308	0.297	8.27E-05	40.6	24.0	47.6	57.0	136.9	92.9	83.4	42.3	104.4	0.41
564	*Tub*	1.626	0.372	9.18E-05	35.6	58.2	63.0	47.5	10.1	21.3	18.3	51.1	16.5	3.1
565	*Chsy3*	1.395	0.319	9.33E-05	52.2	64.7	56.1	44.3	20.2	16.8	25.1	54.3	20.7	2.6
566	*Ndufb4*	-1.064	0.244	9.35E-05	36.4	39.7	33.2	47.5	83.0	87.3	74.3	39.2	81.5	0.48
567	*2210011C24Rik*	-1.014	0.233	9.57E-05	48.9	70.2	40.0	62.2	108.8	116.4	108.6	55.3	111.3	0.50
568	*Dcaf12l1*	-1.190	0.273	9.97E-05	43.9	44.4	65.5	36.9	78.5	108.5	140.6	47.7	109.2	0.44
569	*Chrdl1*	1.121	0.258	0.000102258	96.9	87.8	103.8	154.0	55.0	55.9	41.1	110.6	50.7	2.2
570	*Bmp7*	-1.334	0.307	0.000105671	18.2	17.6	24.7	23.2	56.1	51.5	50.3	20.9	52.6	0.40
571	*2210407C18Rik*	-1.767	0.408	0.000106142	146.6	72.1	155.7	188.9	317.5	646.7	473.2	140.8	479.2	0.29
572	*Slc1a2*	1.542	0.357	0.000114805	94.4	75.8	56.1	40.1	22.4	17.9	28.6	66.6	23.0	2.9
573	*Wdr78*	1.156	0.268	0.000118098	62.9	91.5	78.3	97.1	30.3	35.8	44.6	82.4	36.9	2.2
574	*Itgb8*	1.197	0.278	0.000121008	92.7	73.9	64.7	89.7	23.6	40.3	41.1	80.3	35.0	2.3
575	*Myl4*	2.348	0.547	0.000128645	675.6	411.3	1480.2	1694.5	240.1	214.8	172.6	1065.4	209.2	5.1
576	*Rpl32*	-1.510	0.353	0.000132192	13.2	14.8	12.8	22.2	40.4	49.2	43.4	15.7	44.4	0.35
577	*Chrna2*	-1.822	0.427	0.000144248	29.0	12.0	17.9	12.7	78.5	82.8	29.7	17.9	63.7	0.28
578	*Adgre4*	-1.106	0.260	0.000146647	43.1	61.9	83.4	106.6	148.1	167.8	157.7	73.7	157.9	0.47
579	*Il6*	2.190	0.516	0.000152668	27.3	25.0	27.2	23.2	7.9	4.5	4.6	25.7	5.6	4.6
580	*Sfrp4*	1.762	0.415	0.000157644	31.5	36.0	31.5	33.8	10.1	7.8	11.4	33.2	9.8	3.4
581	*Slc9a2*	-1.068	0.252	0.000162517	45.5	36.0	46.8	51.7	113.3	99.6	69.7	45.0	94.2	0.48
582	*Ms4a4a*	-1.048	0.249	0.000181216	89.4	67.5	116.5	144.5	158.2	254.0	234.3	104.5	215.5	0.48
583	*Tm6sf2*	-1.391	0.332	0.000191935	13.2	23.1	26.4	21.1	52.7	66.0	45.7	21.0	54.8	0.38
584	*Tnc*	1.002	0.239	0.000193076	238.5	171.0	279.9	300.7	88.6	110.8	171.5	247.5	123.6	2.0
585	*Fbxl2*	1.180	0.282	0.000196209	62.9	66.5	72.3	60.1	38.1	23.5	25.1	65.5	28.9	2.3
586	*Fetub*	3.143	0.751	0.000196962	151.5	177.5	60.4	100.2	29.2	9.0	3.4	122.4	13.9	8.8
587	*Klrb1b*	2.011	0.485	0.000228499	31.5	58.2	28.1	27.4	11.2	11.2	4.6	36.3	9.0	4.0
588	*Exd1*	1.704	0.412	0.000241479	33.9	37.9	29.8	30.6	10.1	10.1	10.3	33.1	10.2	3.3
589	*Asphd2*	1.042	0.253	0.000249509	85.3	101.7	82.5	86.5	57.2	41.4	30.9	89.0	43.2	2.1

	A	B	C	D	E	F	G	H	I	J	K	L	M	N
3	***Gene symbol***	***log2FoldChange***	***lfc SE***	***p value adjusted***	***KI1***	***KI2***	***KI3***	***KI4***	***WT1***	***WT2***	***WT3***	***KI Avg***	***Wt Avg***	***fold change***
590	*Shox2*	1.318	0.320	0.000254675	54.6	45.3	63.8	49.6	15.7	23.5	25.1	53.3	21.5	2.5
591	*Pcp4*	-1.556	0.380	0.00027718	18.2	16.6	17.0	47.5	65.1	88.4	62.9	24.8	72.1	0.34
592	*Sln*	3.068	0.749	0.000279156	91.9	53.6	239.9	268.0	20.2	30.2	8.0	163.4	19.5	8.4
593	*Gcgr*	1.530	0.374	0.000289899	40.6	34.2	56.1	55.9	22.4	13.4	12.6	46.7	16.1	2.9
594	*Gdf15*	2.740	0.678	0.000341667	276.6	454.8	118.2	85.5	22.4	42.5	40.0	233.8	35.0	6.7
595	*Spats1*	2.195	0.544	0.000348995	19.0	21.3	28.9	27.4	5.6	3.4	6.9	24.2	5.3	4.6
596	*Rnf115*	1.037	0.257	0.000358185	2558.5	3816.4	1973.7	3670.6	1584.1	1372.9	1436.8	3004.8	1464.6	2.1
597	*Phgdh*	1.055	0.263	0.000380681	68.7	75.8	68.1	74.9	30.3	44.8	28.6	71.9	34.5	2.1
598	*Fgf12*	1.427	0.357	0.000401564	39.7	50.8	68.1	43.3	13.5	24.6	18.3	50.5	18.8	2.7
599	*Fam46b*	1.984	0.497	0.000413165	35.6	45.3	41.7	128.7	7.9	19.0	20.6	62.8	15.8	4.0
600	*Kcnc3*	-1.406	0.353	0.000430311	13.2	14.8	30.6	23.2	46.0	55.9	60.6	20.5	54.2	0.38
601	*Fzd9*	1.131	0.288	0.000518907	53.0	60.1	65.5	51.7	29.2	25.7	24.0	57.6	26.3	2.2
602	*Clec18a*	-1.568	0.399	0.000521728	9.9	12.0	13.6	12.7	34.8	45.9	26.3	12.1	35.6	0.34
603	*Neil2*	1.355	0.345	0.000527113	72.0	66.5	51.0	44.3	14.6	33.6	20.6	58.5	22.9	2.6
604	*Slc1a1*	1.673	0.426	0.000530197	378.4	496.4	204.2	657.3	111.1	158.9	138.3	434.1	136.1	3.2
605	*Fam19a2*	2.188	0.558	0.000538393	29.0	32.4	15.3	26.4	2.2	6.7	8.0	25.8	5.7	4.6
606	*Cyp2ab1*	-1.524	0.389	0.00055093	11.6	14.8	19.6	15.8	26.9	58.2	48.0	15.4	44.4	0.35
607	*Rab3c*	2.711	0.693	0.000556999	28.2	37.9	56.1	5.3	5.6	4.5	4.6	31.9	4.9	6.5
608	*Duoxa1*	-1.551	0.396	0.000558357	14.9	13.9	10.2	8.4	29.2	36.9	38.9	11.9	35.0	0.34
609	*Tnni1*	1.721	0.442	0.000592317	26.5	30.5	60.4	71.7	14.6	13.4	14.9	47.3	14.3	3.3
610	*Flywch2*	1.024	0.263	0.000597097	60.4	72.1	60.4	73.9	32.5	31.3	34.3	66.7	32.7	2.0
611	*Tmem202*	1.232	0.317	0.000610812	43.1	63.8	57.8	65.4	20.2	32.4	20.6	57.5	24.4	2.4
612	*Gfra4*	-1.340	0.348	0.000711765	22.4	29.6	31.5	70.7	84.1	116.4	89.2	38.5	96.6	0.40
613	*Col14a1*	1.112	0.291	0.000787892	2510.5	1833.8	3459.8	2066.9	904.3	1118.9	1401.3	2467.8	1141.5	2.2
614	*Klrk1*	1.241	0.326	0.000826267	50.5	61.9	44.2	42.2	23.6	16.8	22.9	49.7	21.1	2.4
615	*Car9*	1.857	0.488	0.000834167	59.6	18.5	30.6	38.0	11.2	11.2	8.0	36.7	10.1	3.6
616	*Clcn1*	-2.120	0.558	0.000848406	32.3	13.9	48.5	73.9	233.4	148.8	165.7	42.1	182.6	0.23
617	*Stmnd1*	1.880	0.495	0.000858832	35.6	29.6	28.1	16.9	5.6	9.0	8.0	27.5	7.5	3.7
618	*Hist1h2be*	-1.017	0.268	0.000861432	27.3	32.4	40.8	44.3	65.1	82.8	70.9	36.2	72.9	0.50
619	*Kcnj6*	1.870	0.492	0.000861632	58.8	37.0	27.2	98.1	6.7	15.7	22.9	55.3	15.1	3.7
620	*Adcy2*	1.206	0.318	0.000863861	43.1	49.0	60.4	48.5	25.8	21.3	18.3	50.2	21.8	2.3
621	*Xcl1*	1.481	0.390	0.000871053	38.1	59.2	30.6	35.9	14.6	15.7	13.7	40.9	14.7	2.8
622	*Nppb*	1.791	0.472	0.000874871	57423.4	89310.8	28567.8	37907.1	19807.2	11167.0	15227.3	53302.3	15400.5	3.5
623	*Alox8*	2.918	0.771	0.000901709	15.7	9.2	16.2	39.0	2.2	2.2	3.4	20.0	2.6	7.6
624	*Hbegf*	1.363	0.361	0.000935813	1570.7	2688.8	1049.8	2347.6	862.7	674.7	695.0	1914.2	744.1	2.6
625	*Pdk4*	-1.734	0.461	0.000975788	9108.8	8780.9	9464.2	5176.2	19416.8	45384.2	16355.5	8132.5	27052.2	0.30
626														

Table S5 Echocardiographic parameters of *Csrp3* +/+ (WT) and *Csrp3* +/- (HET) hearts (mean +/- SEM)

		Csrp3 +/+	*Csrp3* +/-	
FS	%	42.2 ± 1.7	39.0 ± 2.0	n.s.
LVEDD	mm	3.41 ± 0.06	3.47 ±0.08	n.s.
LVESD	mm	1.97 ± 0.08	2.12 ± 0.10	n.s.
LVAWD	mm	1.10 ± 0.03	1.05 ± 0.04	n.s.
LVPWD	mm	0.89 ± 0.02	0.89 ± 0.04	n.s.
n		14	13	

Table S6 Echocardiographic parameters of *Csrp3* +/-, *Csrp3* KI/- and *Csrp3* -/- hearts (mean ± SEM)

		***Csrp3* +/-**	***Csrp3* KI/-**	***Csrp3* -/-**
n		5	5	5
FS	%	46.8 ± 2.5	12.2 ± 2.2****	12.1 ± 2.1****
LVEDD	mm	3.46 ± 0.09	4.34 ± 0.15**	4.15 ± 0.17*
LVESD	mm	1.84 ± 0.08	3.8 ± 0.2****	3.7 ± 0.2****
LVAWD	mm	1.09 ± 0.03	1.04 ± 0.02	1.03 ± 0.05
LVPWD	mm	0.89 ± 0.02	0.94 ± 0.08	0.93 ± 0.04
HR	bpm	486 ± 4	483 ± 1	476 ± 5
HW/TL	mg/mm	6.5 ± 0.4	8.8 ± 0.2****	8.0 ± 0.1**

* p < 0.05 versus *Csrp3* +/-

** p < 0.01 versus *Csrp3* +/-

**** p < 0.0001 versus *Csrp3* +/-

Table S7 Reagents used for qPCR, Western blotting (WB) and immuno-fluorescence (IF)

a) Taqman Assays for qPCR

(all Applied Biosystems, FAM-MGB unless stated otherwise)

Transcript	***Species***	***Assay ID***	***Comments***
Acta1	Mouse	Mm00808218_g1	
Ankrd1	Mouse	Mm00496512_m1	
Ankrd2	Mouse	Mm00508030_m1	
Bag3	Mouse	Mm00443474_m1	
Bax	Mouse	Mm00432051_m1	
Bcl2	Mouse	Mm00477631_m1	
Casp3	Mouse	Mm01195085_m1	
Col1a1	Mouse	Mm00801666_g1	
Col3a1	Mouse	Mm01254476_m1	
Cryab	Mouse	Mm00515567_m1	(ABcryst)
Csrp3	Mouse	Mm00443379_m1	
Ctgf	Mouse	Mm01192932_g1	
Fhl1	Mouse	Mm04204611_g1	
Fhl2	Mouse	Mm00515781_m1	
Gapdh	Mouse	4352339E	VIC-MGB labelled
Hspa1a	Mouse	Mm01159846_s1	(Hsp70)
Hspa8	Mouse	Mm01731394_gH	(Hsc70)
Hspb1	Mouse	Mm00834384_g1	(Hsp27)
Hspb7	Mouse	Mm04210487_m1	
Lox	Mouse	Mm00495386_m1	
Myh6	Mouse	Mm00440359_m1	
Myh7	Mouse	Mm00600555_m1	
Nppa	Mouse	Mm01255748_g1	
Nppb	Mouse	Mm01255770_g1	
Rcan1	Mouse	Mm00627762_m1	
Tgfb1	Mouse	Mm01178820_m1	
Trp53	Mouse	Mm01731290_g1	
Gapdh	Rat	Rn99999916_s1	VIC-MGB labelled
hrGFP	n/a	ID AIY9YGZ	custom designed
CSRP3	Homo s.	Hs00185787_m1	human-specific

b) Antibodies (Dilutions for Western blotting unless stated.)

Protein	***Type***	***Clone/Cat. No.***	***Source***	***Dilution (WB)***
ABcrystallin	mouse	ab13496	Abcam	1:3000
Alpha-actinin	rabbit	ab68167	Abcam	1:1000
Alpha-actinin	mouse	EA53/ A7811	Sigma	1:500 (IF)
Ankrd1/Carp	rabbit	ab88456	Abcam	1:500
BAG3	rabbit	10599-1-AP	Proteintech	1:2000
Beta-myosin	mouse	NOQ7.5.4D/M8421	Sigma	1:2000
DSCR1 (Rcan1.4)	rabbit	D6694	Sigma	1:250
Fhl1	mouse	ab76912	Abcam	1:200
Fhl2	mouse	K0055-3	MBL	1:500
Gapdh	rabbit	ABS16	Millipore	1:3000
GFP	mouse	11814460001	Roche	1:1000
HA-tag	rat	3F10/11867423001	Roche	1:1000
hrGFP	rabbit	240141	Agilent	1:500
Hsc70	rabbit	ADI-SPA-815-D	Enzo	1:1000
Hsp70	rabbit	4872	CST	1:1000
Hsp27	rabbit	2442	CST	1:1000
HspB7	rabbit	15700-1AP	Proteintech	1:1000
LC3	rabbit	4108	CST	1:300
MLP	mouse	79D2	[1]	1:1000
pan-actin	rabbit	A2066	Sigma	1:1000
PKCalpha	rabbit	2056	CST	1:250
pT638/641PKCα/βII	rabbit	9375	CST	1:300
titin m8	rabbit	m8	[2]	1:50 (IF)
Ubiquitin	rabbit	Z0458	DAKO	1:2000

References Table S7

[1] C. Geier, K. Gehmlich, E. Ehler, S. Hassfeld, A. Perrot, K. Hayess, N. Cardim, K. Wenzel, B. Erdmann, F. Krackhardt, M.G. Posch, K.J. Osterziel, A. Bublak, H. Nagele, T. Scheffold, R. Dietz, K.R. Chien, S. Spuler, D.O. Furst, P. Nurnberg, C. Ozcelik, Beyond the sarcomere: CSRP3 mutations cause hypertrophic cardiomyopathy, Hum Mol Genet 17(18) (2008) 2753-65.

[2] W.M. Obermann, M. Gautel, F. Steiner, P.F. van der Ven, K. Weber, D.O. Furst, The structure of the sarcomeric M band: localization of defined domains of myomesin, M-protein, and the 250-kD carboxy-terminal region of titin by immunoelectron microscopy, J Cell Biol 134(6) (1996) 1441-53.
